# A New Stereoselective Approach to the Substitution of Allyl Hydroxy Group in *para*-Mentha-1,2-diol in the Search for New Antiparkinsonian Agents

**DOI:** 10.3390/molecules28217303

**Published:** 2023-10-27

**Authors:** Alexandra V. Podturkina, Oleg V. Ardashov, Konstantin P. Volcho, Nariman F. Salakhutdinov

**Affiliations:** Department of Medicinal Chemistry, N. N. Vorozhtsov Novosibirsk Institute of Organic Chemistry, Siberian Branch, Russian Academy of Sciences, Lavrentiev Ave. 9, 630090 Novosibirsk, Russia; podturkina@nioch.nsc.ru (A.V.P.); ardashov@nioch.nsc.ru (O.V.A.); anvar@nioch.nsc.ru (N.F.S.)

**Keywords:** monoterpene, nucleophilic substitution, synthetic methods, Prottremine, epoxide

## Abstract

Two approaches to the synthesis of *para*-menthene epoxide ((1*S*,5*S*,6*R*)*-***4**) are developed. The first approach includes a reaction between chlorohydrin **7** and NaH in THF. The second involves the formation of epoxide in the reaction of corresponding diacetate **6** with sodium *tert*-butoxide. One possible mechanism of this reaction is proposed to explain unexpected outcomes in the regio- and stereospecificity of epoxide (1*S*,5*S*,6*R*)*-***4** formation. The epoxide ring in (1*S*,5*S*,6*R*)*-***4** is then opened by various *S*- and *O*-nucleophiles. This series of reactions allows for the stereoselective synthesis of diverse derivatives of the monoterpenoid Prottremine **1**, a compound known for its antiparkinsonian activity, including promising antiparkinsonian properties.

## 1. Introduction

Parkinson’s disease (PD) is a neurodegenerative disorder characterized by the progressive loss of dopamine-producing neurons in the *substantia nigra* region of the brain. The main features of PD include motor symptoms such as tremor, slowness of movement, rigidity, and difficulty in movement [1]. Additionally, patients experience non-motor symptoms such as sleep disorders, pain, fatigue, and psychiatric symptoms [2,3,4]. In 2016, an estimated 6.1 million individuals worldwide received a Parkinson’s disease diagnosis, which was 2.4 times higher than in 1996 [5]. While most PD cases are diagnosed between the ages of 60 and 65, cases of young onset (<50 years) and juvenile cases (<21 years) have also been identified [6].

Presently, there is no cure for PD, and the treatment’s objective is to alleviate the impact of its symptoms. For over 50 years, levodopa has been the gold standard in ameliorating PD motor symptoms [7]. However, challenges such as levodopa-induced dyskinesia and OFF symptoms continue to persist [8]. Approximately 55.9% of patients experience dyskinesia as a result of levodopa therapy [9]. Therefore, the primary goal remains to investigate novel therapeutic agents with innovative mechanisms of action for PD treatment [10,11].

**Figure 1:**
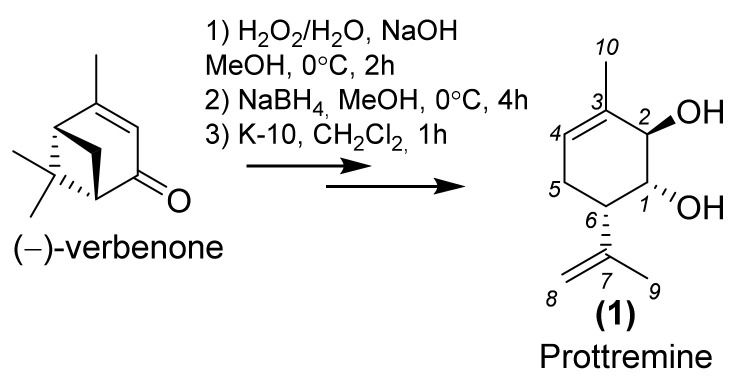
Synthesis of Prottremine (**1**).

Various monoterpenes and their derivatives, including myrcene [12], cytronellol [13,14], perillyl alcohol [15,16], geraniol [17,18], isoborneol [19], limonene [20], and carvacol [21], have demonstrated potent anti-PD activity. Moreover, promising anti-PD activity has been shown for monoterpenoid (1*R*,2*R*,6*S*)-3-methyl-6-(prop-1-en-2-yl)cyclohex-3-en-1,2-diol **1** (Prottremine) (Figure 1), synthesized from verbenone. Notably, Prottremine has exhibited significant antiparkinsonian activity in various models of mice and rats, effectively enhancing markers of locomotor and exploratory activity in animals. Its performance is comparable to that of levodopa, a well-established therapeutic agent [22,23]. Additionally, it is pertinent to highlight the low acute toxicity of the compound (LD_50_ 4250 mg/kg, mice) [24].

**Figure 2:**
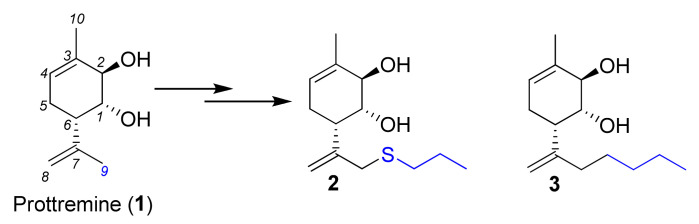
Prottremine derivatives.

Consequently, all eight stereoisomers of diol **1** were synthesized with high optical purity to examine the impact of the configuration of compound **1** on its biological activity. Using the neurotoxin 1-methyl-4-phenyl-1,2,3,6-tetrahydropyridine (MPTP) model of PD [25], it was shown that the absolute configuration of **1** significantly influenced its antiparkinsonian activity [22]. Furthermore, an investigation into the significance of four functional groups (two hydroxyl groups and two double bonds) in Prottremine relating to its antiparkinsonian effect was conducted [26]. The findings indicated that the pronounced antiparkinsonian activity required the presence of all four functional groups in the molecule. This led us to proceed with the synthesis of diverse derivatives of Prottremine, involving modifications at allyl position *9* while retaining the integrity of the four functional groups. Among these derivatives of Prottremine, compounds featuring propylthio **2** and butyl **3** moieties exhibited a notable antiparkinsonian effect. They successfully restored all the parameters of locomotor activity, displaying the same level of efficiency as Prottremine (Figure 2) [27].

**Figure 3:**
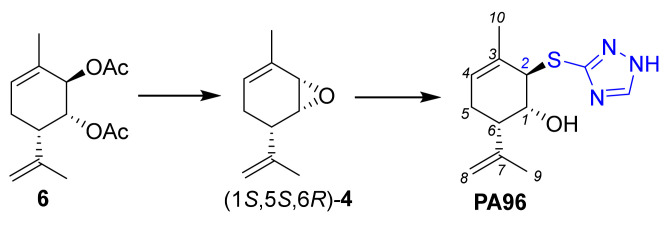
Synthesis of PA96.

Based on the information above, we focused our interest on the insertion of substituents at other allyl positions, without changing the absolute configuration of Prottremine. Recently, we discovered the Prottremine derivative **PA96** (Figure 3) coupled with a 1*H*-1,2,4-triazol-3-ylthio substituent at the *2* allyl position. This derivative exhibited the remarkable alleviation of symptoms of PD in both MPTP-induced and haloperidol-induced models. **PA96** displayed this effect at a dose as low as 1 mg/kg, in contrast to the active dose of Prottremine, which is 20 mg/kg. **PA96** exhibited an ability to safeguard cultured dopamine neurons from spontaneous and toxin-induced death [28]. While our previous publication primarily focused on the pharmacological implications, we briefly introduced the synthesis of **PA96** from epoxide (1*S*,5*S*,6*R*)*-***4** without delving into the details. Here, we discuss in detail this original approach, including stereochemical issues, possible mechanisms, and the scope of the reaction.

## 2. Results and Discussion

### 2.1. Synthesis of Epoxide (1S,5S,6R)-4

In this work, we used commercially available (−)-verbenone with an enantiomeric excess of 58%. Further, if necessary (e.g., in the presence of high biological activity), it will be possible to synthesize individual enantiomers of the target product based on verbenones in high enantiomeric excess. The synthesis of the corresponding verbenones can be performed in three steps based on the respective α-pinenes [29].

We adopted a two-step approach to synthesize the *O*- and *S*-derivatives of diol **1** at allyl position *2*, maintaining the *R*-configuration at this asymmetric center. This approach involves the formation of an epoxide followed by its opening with nucleophiles. Epoxides, particularly those conjugated with an allylic bond, are among the most important intermediate and target compounds in organic synthesis. Numerous methods have been developed for epoxide synthesis; the regioselectivity of their opening by various nucleophiles has been studied in detail. The outcome often depends on such factors as the reaction conditions, the catalysts used, and the structural features of the substrates [30,31,32].

**Figure 4:**
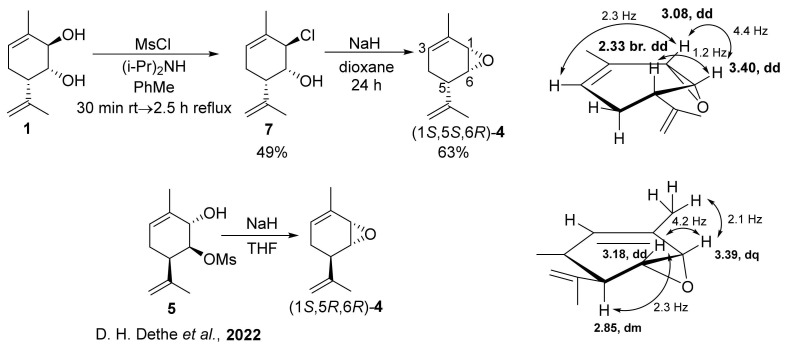
Synthesis of epoxide (1*S*,5*S*,6*R*)*-***4** from chlorohydrin **7** [34].

For our study, the choice of the synthesis method depended on the availability of appropriate intermediates. We recently developed a method for the synthesis of chlorohydrin **7** from diol **1** (yield, 49%) [26] and diacetate **6** [33]. In this work, we showed that the interaction between chlorohydrin **7** and NaH in dioxane made it possible to synthesize epoxide (1*S*,5*S*,6*R*)*-***4** with a preparative yield of 63% (Figure 4). Dethe D. H. et al. [34] also synthesized an epimeric epoxide (1*S*,5*R*,6*R*)*-***4** by treating mesylate **5** with NaH in THF. The NMR spectra of (1*S*,5*R*,6*R*)*-***4** and (1*S*,5*S*,6*R*)*-***4** are quite different and thus easily distinguishable. Along with the noticeable differences in the chemical shifts of the signals, the W-constant = 2.3 Hz is observed in the ^1^H NMR spectrum of compound (1*S*,5*S*,6*R*)-**4** between proton H-1 and proton H-3 of the double bond. In the case of (1*S*,5*R*,6*R*)-**4**, the W-constant of 2.3 Hz is observed with the protons of the methyl group. The constants between protons H-1 and H-6 are approximately the same (4.4 and 4.2 Hz); furthermore, the vicinal constant for (1*S*,5*R*,6*R*)-**4** is higher than that for (1*S*,5*S*,6*R*)-**4** (2.3 Hz vs. 1.2 Hz).

**Figure 5:**
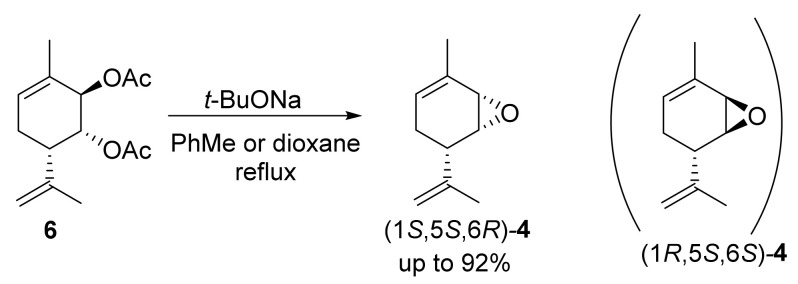
Synthesis of epoxide (1*S*,5*S*,6*R*)*-***4** from diacetate **6**.

However, our findings revealed that the one-pot approach to the synthesis of epoxides using diacetate **6** was more promising than the one using chlorohydrins **7**. This approach included the saponification of one ester group and intramolecular substitution with epoxy ring closure (Figure 5). It is rarely used in the literature; however, it has been applied in steroid chemistry, where the reaction proceeds with the *trans*-diaxial arrangement of acetate groups [35].

When choosing the reaction conditions, we had to consider the fact that, after initial saponification, it was necessary to ensure that saponification did not proceed further, but the epoxide was closed. To address this challenge, it seemed logical to use a high-boiling-point solvent and a spatially hindered base. Indeed, the use of NaH as a base or THF as a solvent resulted in the undesired formation of a notable quantity of diol **1** impurities. Therefore, we chose *t*-BuONa as a base. In the case of a *t*-BuOK, the processing of the reaction mixture deteriorates because of the higher solubility of the base. Considering the selection of solvents, both dioxane and toluene exhibited promising outcomes. However, toluene was found to be a more practical solvent because its azeotropic distillation was sufficient to dry it, while the distillation of dioxane over Na under an atmosphere of argon was required. At the end of the reaction, hexane was added to the suspension to facilitate complete precipitation, and filtration was conducted. In the case of dioxane, a fivefold excess of hexane was required to initiate precipitation, whereas a double excess sufficed for toluene. Further, we optimized the reaction conditions to elaborate a preparative synthesis method for epoxide (1*S*,5*S*,6*R*)*-***4**. Parameters such as the molar ratio of reagents and the reaction time were systematically varied during optimization (Table 1).

As summarized in Table 1, both the reaction duration and the molar ratio of reagents greatly influenced the yield of epoxide (1*S*,5*S*,6*R*)*-***4**. The formation of the mixture of monoacetates **8** and **9** was observed as the amount of *t*-BuONa decreased. The results showed that the 4:1 molar ratio of reagents (*t*-BuONa: diacetate **6**) and the reaction duration of 1.5 h could provide a better yield (81%, entry 1).

**Figure 6:**
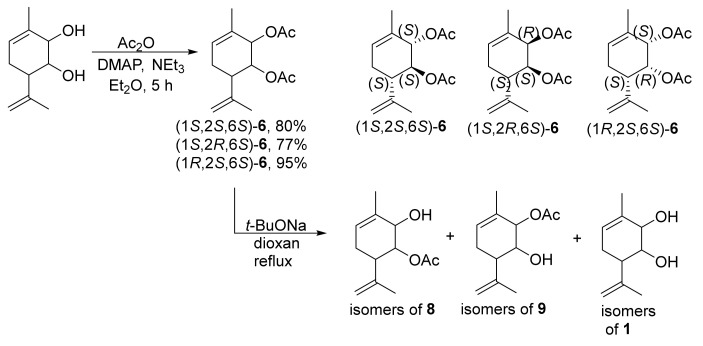
Synthesis of diacetate **6** stereoisomers.

Two key aspects should be highlighted before considering the possible mechanism. First, the *trans*-diaxial arrangement of acetate groups is necessary for the reaction between diacetate **6** and *t*-BuONa to occur. To explore this, we prepared three other stereoisomeric diacetates ((1*S*,2*S*,6*S*)-**6**, (1*S*,2*R*,6*S*)-**6** and (1*R*,2*S*,6*S*)-**6**) from the recently synthesized stereoisomers of diol **1** [22]. The synthesis of the remaining four possible stereoisomers was not required because they were enantiomers of the four already synthesized stereoisomers and thus possessed identical reactivity. After performing the reactions of these two *cis*-diacetates and one *trans*-diacetate with *t*-BuONa, only mixtures of partially and fully saponified products were obtained (Figure 6).

**Figure 7:**
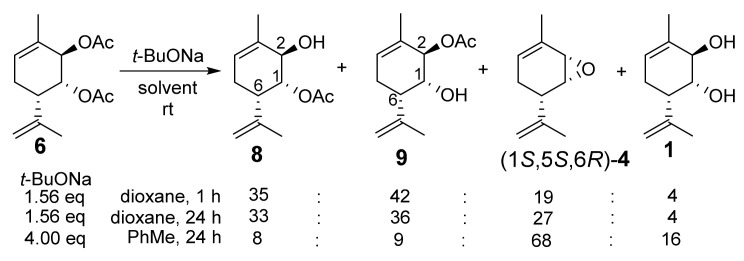
Reaction between diacetate **6** and *t*-BuONa at room temperature.

The second noteworthy aspect, the stereochemistry of epoxide (1*S*,5*S*,6*R*)*-***4**, is more significant. Indeed, why was the individual epoxide (1*S*,5*S*,6*R*)*-***4** formed, rather than the diastereomeric epoxide (1*R*,5*S*,6*R*)*-***4** or a mixture of epoxides (Figure 5)? Generally speaking, the allyl acetate group at position *2* is more spatially accessible, suggesting a straightforward mechanism involving the saponification of one of the acetate groups, followed by the intramolecular substitution of the second acetate group for epoxide ring closure. Therefore, (1*R*,5*S*,6*R*)*-***4** could be the predominant or the only product; however, (1*R*,5*S*,6*R*)*-***4** was not formed at all. Moreover, we carried out the reaction between diacetate **6** and *t*-BuONa at room temperature and with the same ratio of reagents and solvents as that used for the reaction at boiling temperature (1.56 eq *t*-BuONa in the case of dioxane and 4.00 eq *t*-BuONa for toluene, which gave the maximum yield) and isolated a mixture of monoacetates **8** and **9**, diol **1**, and epoxide (1*S*,5*S*,6*R*)-**4** at a molar ratio of 35:42:19:4 for dioxane after 1 h and 33:36:27:4 after 24 h, respectively. The same reaction in toluene for 24 h gave a mixture of monoacetates **8** and **9**, diol **1**, and epoxide (1*S*,5*S*,6*R*)-**4** at a molar ratio of 8:9:68:16, respectively (Figure 7). Note that monoacetates differ strongly in their ^1^H NMR spectra. For compound **8**, the H-1e signal is dd; it has interaction constants with the H-2e (2.8 Hz) and H-6a (1.7 Hz) protons and is located in the characteristic region of esters (5.12 ppm). Meanwhile, for compound **9**, the signal of the H-1e proton (ddd) lies in the region of alcohols (3.91 ppm), it has constants similar to those of the H-2e (2.6 Hz) and H-6a (1.7 Hz) protons, and it splits into an OH group with a constant of 4.0 Hz. The corresponding H-2e signals are less informative; they are multiplets with small constants. For compound **8**, they appear at 3.80–3.84 ppm, and for product **9**, they appear at 5.10–5.12 ppm (copies of ^1^H NMR spectra can be found in the Supplementary Materials).

**Figure 8:**
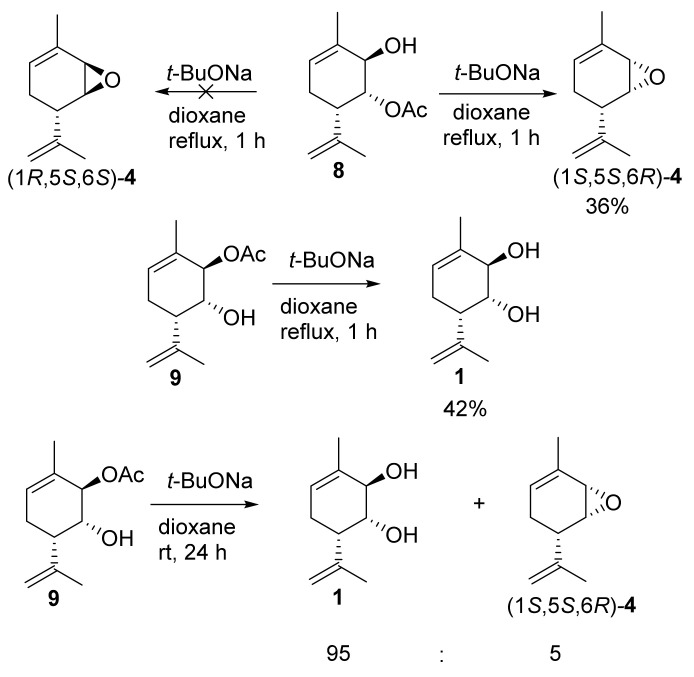
Reaction between diacetate **8** and **9** *t*-BuONa.

Finally, the reaction of individual monoacetates **8** or **9** with *t*-BuONa at a high temperature showed that only monoacetate **8** with a hydroxyl group at the *2* allyl position gave epoxide (1*S*,5*S*,6*R*)*-***4**. On the other hand, the regioisomeric monoacetate **9** simply underwent further saponification to form diol **1**, which was obtained in a 42% preparative yield. When this reaction was carried out at room temperature, a mixture of the complete saponification product, diol **1**, and a small amount of epoxide (1*S*,5*S*,6*R*)-**4** was formed at a 95:5 ratio (Figure 8).

**Figure 9:**
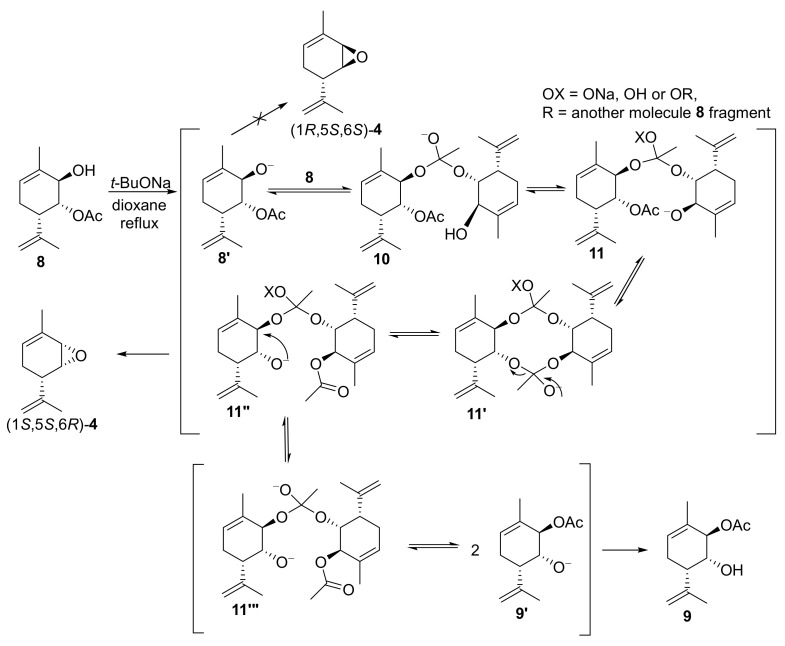
Proposed mechanism for the reaction between diacetate **6** and *t*-BuONa.

In order to interpret the obtained experimental data, we suggest that the reaction between diacetate **6** and *t*-BuONa starts with the saponification of the spatially more accessible allyl acetate group at position *2*. However, the obtained alkoxide anion at molecule **8′** did not substitute the second acetate group. Instead, it appeared to give rise to a more complex leaving group engaged in an interaction with another molecule of monoacetate **8** or its alkoxide anion **8′** reacting with the acetate group. The anion derived from the carbonyl group could either undergo protonation or transform into some orthoester derivatives (denoted as R in the intermediate **11**). The second hydroxyl anion received immediately or after deprotonation interacted with the second acetate group. However, by this point, acetate transfer had already taken place. Consequently, intramolecular substitution occurred upon the formation of alkoxide anion **11″**, leading to a complex leaving group, eventually resulting in the formation of epoxide (1*S*,5*S*,6*R*)*-***4**. This suggests that the reaction presumably occurred through the formation of some intermolecular complex, potentially at least a dimeric one, which is quite acceptable for low-polarity toluene (Figure 9).

The experimental data obtained at different temperatures can simultaneously be explained using this mechanism. Indeed, at the boiling point of dioxane or toluene, diol **1** is formed from monoacetate **9**, only epoxide (1*S*,5*S*,6*R*)-**4** is formed from monoacetate **8**, and only epoxide (1*S*,5*S*,6*R*)-**4** is formed from diacetate **6**. In other words, in the case of diacetate **6**, the formation of monoacetate **9** and its hydrolysis product diol **1** is either not observed at all or is insignificant. However, the formation of epoxide (1*S*,5*S*,6*R*)-**4** in a small amount is also observed at room temperature, and the formation of monoacetates **8** and **9** occurs at a close ratio, even with the slight predominance of monoacetate **9**. In addition, diol **1** is formed from monoacetate **9** at room temperature, with only traces of epoxide (1*S*,5*S*,6*R*)-**4**, which is presumably formed by transesterification to monoacetates **8** and further cyclization. The formation of diastereomeric epoxide (1*R*,5*S*,6*S*)-**4** was not observed in any of the experiments, i.e., direct cyclization from monoacetate **9** did not occur. We assume that the formation of anion **9′** does not occur in the case of sterically hindered *t*-BuONa directly by the hydrolysis of diacetate **6**. Most likely, anion **8′** is initially formed and then dimerizes; the transformations already described above occur in the dimer, and the corresponding monomeric anion **9′** is finally formed as a result of the cleavage of dianion **11″′**. Subsequent treatment with water allowed us to obtain regioisomeric monoacetate **9**. Therefore, by varying the temperature, we can influence the ratio of the reaction rates. At high temperatures, the formation of epoxide (1*S*,5*S*,6*R*)-**4** based on the dimeric anion **11″** occurs much more rapidly; however, at room temperature, the rate of this process decreases greatly, equilibrium is attained faster between the monoacetate anions **8′** and **9′**, and complete hydrolysis occurs. Note that monoacetate **9**, diacetate **6**, and diol **1** are separated by column chromatography, whereas epoxide (1*S*,5*S*,6*R*)-**4** is converted to diol **1** on SiO_2_. Hence, if the target compound is epoxide (1*S*,5*S*,6*R*)-**4**, no aqueous treatment is performed, and both excess *t*-BuONa and all the salt-like anionic particles remaining in the reaction mixture, additionally precipitated with hexane, are separated by filtration. Next, the careful distillation of the solvent is required because epoxide (1*S*,5*S*,6*R*)-**4** is also volatile. At this stage, it became clear that although the yield in dioxane is higher than that in toluene, in addition to the larger amount of hexane that needs to be added when processing the reaction mixture, it is also necessary to leave larger amounts of solvent in the product to avoid its loss during distillation. If epoxide (1*S*,5*S*,6*R*)-**4** needs to contain no traces of solvent, the distillation losses will be lower for toluene. If dioxane does not interfere with the subsequent steps, it may be preferable to use dioxane. In our case, toluene was also chosen because subsequent interaction with alcohols was carried out in toluene; therefore, it was possible to do so without distilling the solvent off at all.

**Figure 10:**
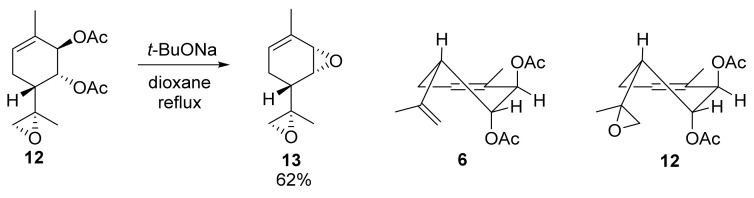
Reaction with diacetate epoxydiol **12** and *t*-BuONa.

The method employed for epoxide synthesis from diacetate accommodated substituents at the 7,8 bond. In particular, we demonstrated the viability of this reaction with diacetate epoxydiol **12** [36], characterized by the *trans*-diaxial arrangement of acetate groups. As a result, we synthesized diepoxide **13** with a 62% yield (Figure 10).

### 2.2. Reaction of Epoxide (1S,5S,6R)-4 with Nucleophiles

The next stage of our work involved the synthesis of various *O*- and *S*-containing derivatives based on epoxide (1*S*,5*S*,6*R*)*-***4**. To create ethers, we carried out the reaction between epoxide (1*S*,5*S*,6*R*)*-***4** and different primary alcohols in the presence of *p*-TsOH × H_2_O in toluene. Relative acid reagents are often used, including camphorsulfonic acid (CSA) [37,38,39] and pyridinium *p*-toluenesulfonate (PPTS) [40,41,42,43]. It is known that *p*-TsOH can be used to activate epoxides upon opening with amines [44,44,45,46]. Because of the volatility of epoxide (1*S*,5*S*,6*R*)*-***4**, toluene was not completely evaporated at the previous stage; therefore, we report the yields of the products for two stages in terms of diacetate **6**. Unfortunately, this method proved unsuitable for the synthesis of derivatives containing secondary and tertiary alcohols. Furthermore, a moderate yield was observed for the ethers of primary alcohols. This was due to the competing opening of the epoxide, along with the displacement of the double bond through the mechanism of allyl nucleophilic substitution S_N_2′, and the side reaction of alcohol insertion to the double bond of the isopropenyl group. To reduce the role of this side process, the reaction was carried out for 1 h. In the case of the reaction between epoxide (1*S*,5*S*,6*R*)*-***4** and some primary alcohols (methanol and ethanol), both the target products **14** (33%) and **16** (26%) and the isomeric compounds **15** (10%) and **17** (16%) were obtained.

**Figure 11:**
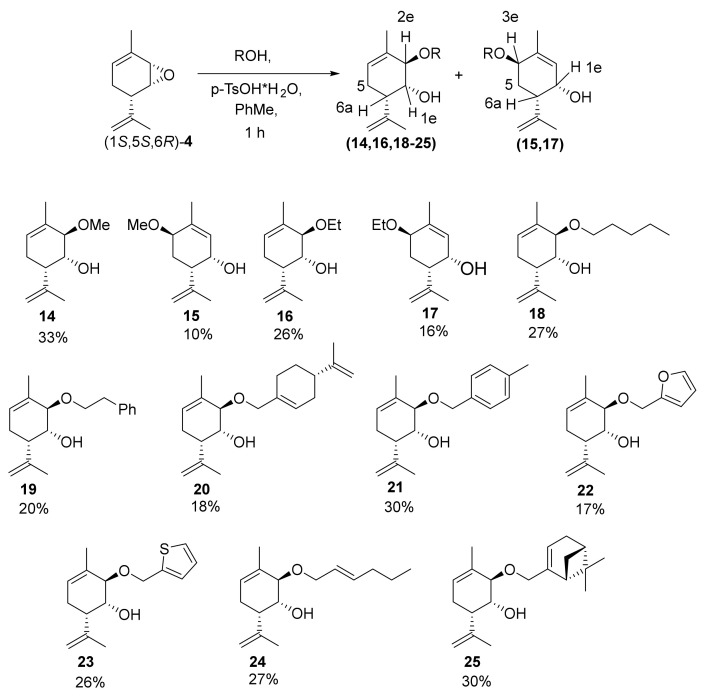
Synthesis of Prottremine derivatives with *O*-substituents.

Further, the formation of an isomeric ether with the rearrangement of the double bond was observed in a small amount, and the target compounds were synthesized with preparative yields via a reaction with primary alcohols: *n*-AmOH (**18**, 27%), β-phenylethanol (**19**, 20%), (−)-perillyl alcohol (**20**, 18%), *para*-tolylmethanol (**21**, 30%), furfural (**22**, 17%), thiophene-2-carbinol (**23**, 26%), 2-hexanol (**24**, 27%), and (−)-myrtenol (**25**, 30%); the yields are given for the two steps calculated with reference to diacetate **6** (Figure 11). No formation of products at the non-allylic first position was observed. Proton H-6a occupied an axial position in all the obtained products, as evidenced by the constant of the vicinal spin–spin interaction with proton 5a, which was 11–12 Hz. In addition, for all the products, a small interaction constant for the H-1 and H-6a protons was observed (<2.2 Hz). This indicated that the H-1e proton had an equatorial position. Furthermore, for the main products **14**, **16**, **18**–**25**, the interaction constants of protons H-1e and H-2e were less than 2.2 Hz. The fact that proton H-2 occupies an equatorial position is evidenced by the W-constant with methyl group 10 and the corresponding cross-peak in the HMBC spectra. For isomeric products **15** and **17**, the H-4 proton occupies an equatorial position, as evidenced by the small constants of the spin–spin interaction with the H-5a (3.7 Hz) and H-5e (2.0 Hz) protons.

**Figure 12:**
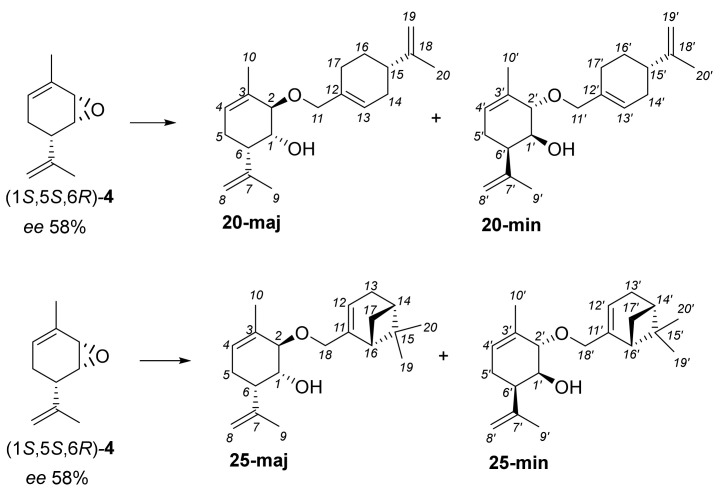
Synthesis of Prottremine derivatives with perillyl alcohol and myrtenol.

Since we used (1*S*,5*S*,6*R*)-**4**, synthesized from (−)-verbenone with an enantiomeric excess of 58%, products **20** and **25** were isolated as mixtures of two diastereomers in the case of perillyl alcohol and myrtenol containing asymmetric centers (Figure 12).

**Figure 13:**
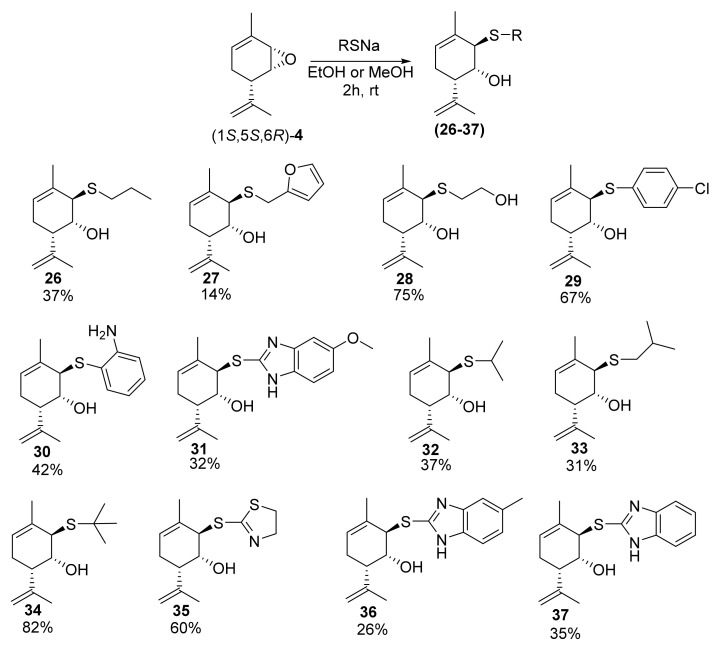
Synthesis of Prottremine derivatives with S-substituents.

Further, we carried out the nucleophilic substitution reaction with epoxide (1*S*,5*S*,6*R*)*-***4** and different thiols. Thiolate anion is a stronger nucleophile and does not require activation using Lewis or Brønsted acids to open the epoxide cycle and synthesize *S*-derivatives, unlike for *O*-derivatives. Compound **26** was synthesized via a reaction between compound (1*S*,5*S*,6*R*)*-***4** and *n*-PrSNa (prepared in situ from *n*-PrSH and NaOH or *t*-BuONa) in ethanol with a 44% yield. In a similar way, we prepared *S*-derivatives with furan-2-ylmethylthiol (**27**, 14%), thioethanol (**28**, 75%), *p*-chlorothiophenol (**29**, 67%), *o*-aminothiophenol (**30**, 42%), 5-methoxybenzimidazolylthiol (**31**, 32%), isopropylthiol (**32**, 37%), isobutylthiol (**33**, 31%), *tert*-butylthiol (**34**, 82%), 4,5-dihydrothiazol-2-ylthiol (**35**, 60%), 5-methyl-1*H*-benzo[*d*]imidazol-2-ylthiol (**36**, 26%), and 1*H*-benzo[*d*]imidazol-2-ylthiol (**37**, 35%) (Figure 13).

**Figure 14:**
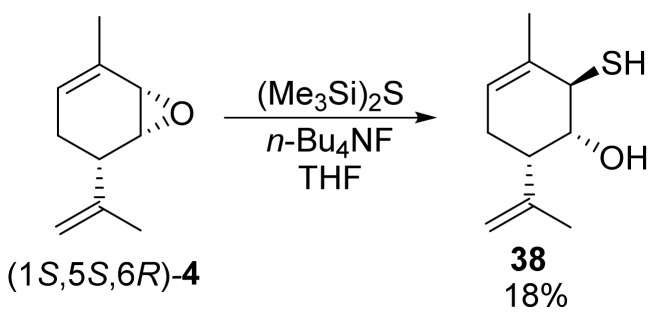
Synthesis of Prottremine derivative with SH group.

At the same time, the derivative with the SH moiety is more difficult to synthesize than those with SR moieties. Using technical-grade Na_2_S × 10H_2_O in dioxane or DMSO, we obtained a mixture containing diol **1** as the main hydrolysis product. Replacement with NaSH × H_2_O also did not provide satisfactory results, since, according to the ^1^H NMR of the reaction mixture, the reagent reacted not only at the epoxide cycle but also at the isopropenyl double bond, leading to the formation of a complex mixture of products with significant resinification. As a result, we managed to synthesize the desired compound **38** using the reagent system (Me_3_Si)_2_S/*n*-Bu_4_N^+^F^−^ in THF [47]. The reaction proceeded for 30 min and the yield of compound **38** was 18% after purification by column chromatography (Figure 14).

## 3. Materials and Methods

### General Methods and Materials

All the commercially available compounds and solvents were of reagent grade and were used without further treatment, unless otherwise noted. General information: (−)-myrtenol >97% (*ee* 95%), (−)-perillyl alcohol 90% (*ee* 95%). Column chromatography (CC): silica gel (SiO_2_; 60–200 µ; Macherey-Nagel); hexane/EtOAc 100:0 → 0:100 and CHCl_3_/EtOH 100:0 → 0:100. GC/MS (purity control and product analysis: Agilent 7890A (Agilent Technologies, Santa Clara, CA, USA) with an Agilent 5975C quadrupole mass spectrometer as a detector, HP-5MS quartz column, 30,000 × 0.25 mm, He (1 atm) as a carrier gas. Optical rotation: polAAr 3005 spectrometer (Optical Activity LTD, Huntingdon, UK, CHCl_3_, EtOH soln.). HR-MS: DFS-Thermo Scientific spectrometer in full scan mode (15–500 *m/z*, 70 eV electron-impact ionization, direct sample introduction) (Thermo Fisher Scientific, Waltham, MA, USA) and Agilent 7200 Accurate Mass Q-TOF GC/MS (70 eV, electron-impact ionization (Agilent Technologies, Santa Clara, CA, USA)). The melting points were determined using a Mettler Toledo FP900 Thermosystem (Greifensee, Switzerland). ^1^H and ^13^C NMR: Bruker Avance-III 600 (Bruker Corporation, Karlsruhe, Germany) apparatus at 600.30 MHz (^1^H) and 150.95 MHz (^13^C), Bruker Avance 400 (Bruker Corporation, Karlsruhe, Germany) apparatus at 400.13 MHz (^1^H) and 100.61 MHz (^13^C), and Bruker DRX 500 (Bruker Corporation, Karlsruhe, Germany) apparatus at 500.13 MHz (^1^H) and 125.76 MHz (^13^C) in CDCl_3_ and d^6^-DMSO; chemical shifts d in ppm rel. to residual CHCl_3_ (d(H) 7.24, d (C) 76.90 ppm), chemical shifts d in ppm rel. to residual DMSO (d(H) 2.50, d (C) 39.84 ppm), J in Hz. Structure determination: by analyzing the ^1^H NMR spectra, including ^1^H-^1^H double resonance spectra and ^1^H-^1^H 2D homonuclear correlation (COSY, NOESY); J-modulated ^13^C NMR spectra (JMOD); and ^13^C-^1^H 2D heteronuclear correlation with one bond and long-range spin–spin coupling constants (C-H COSY, ^1^J(C,H) = 135 Hz; HSQC, ^1^J(C,H) = 145 Hz; HMBC, ^2,3^J(C,H) = 7 Hz). All the target compounds reported in this paper have at least 95% purity. (1*R*,2*R*,6*S*)-3-Methyl-6-(prop-1-en-2-yl)cyclohex-3-ene-1,2-diol **1** was synthesized from (−)-verbenone (*Aldrich*) according to [48]. (1*R*,2*R*,6*S*)-3-Methyl-6-(prop-1-en-2-yl)cyclohex-3-ene-1,2-diyl diacetate **6** was synthesized according to [33]. Isomeric 3-methyl-6-(prop-1-en-2-yl)cyclohex-3-ene-1,2-diacetates ((1*R*,2*S*,6*S*)-**6**, (1*S*,2*R*,6*S*)-**6** and (1*S*,2*S*,6*S*)-**6**) and (1*R*,2*R*,6*R*)-3-methyl-6-((*S*)-2-methyloxiran-2-yl)cyclohex-3-ene-1,2-diyl diacetate **12** were synthesized in a similar way from the corresponding isomers of diol **1** [22] or their epoxyderivatives [49]. Compound **13** and monoacetates **8** and **9** were synthesized a similar way to the preparation method for epoxide (1*S*,5*S*,6*R*)*-***4** in dioxane. Partial saponification was observed in the reaction between (1*S*,2*S*,6*S*)-**6** and *t*-BuONa. For other isomers of diacetate, complete or partial saponification was observed but only the product of complete saponification was isolated in individual form. ^1^H and ^13^C NMR of the reaction mixture of monoacetate **8** and **9** are described in [49]. Compound **12** is described in [36].

The numeration of atoms in the compounds is given to assign the signals in the NMR spectra only and does not coincide with that for the names according to the nomenclature of compounds.

General procedure of (1*S*,5*S*,6*R*)-2-methyl-5-(prop-1-en-2-yl)-7-oxabicyclo[4.1.0]hept-2-ene ((1*S*,5*S*,6*R*)*-***4**) synthesis.

The mixture of sodium *tert*-butoxide (800.7 mg, 8.33 mmol) and 10 mL toluene was warmed to the boiling point of the solvent in a glycerol bath. Then, (1*R*,2*R*,6*S*)-3-methyl-6-(prop-1-en-2-yl)cyclohex-3-ene-1,2-diacetate **6** (525.6 mg, 2.08 mmol) dissolved in 5 mL toluene was added dropwise to the reaction mixture. The mixture was stirred at reflux at 110 °C for 2 h. The reaction mixture was then cooled down, and 30 mL hexane was added. After the end of precipitation, the solution was filtered off from the precipitate and evaporated in vacuo. The yield of epoxide(1*S*,5*S*,6*R*)*-***4** was 249 mg (1.64 mmol, 80%).

The suspension of sodium *tert*-butoxide (622.9 g, 6.48 mmol) in 30 mL of dry dioxane (distilled over Na in an atmosphere of argon) was warmed to the boiling point of the solvent. Next, (1*R*,2*R*,6*S*)-3-methyl-6-(prop-1-en-2-yl)cyclohex-3-ene-1,2-diacetate **6** (1.049 g, 4.15 mmol) dissolved in 30 mL of dioxane was added dropwise to the reaction mixture. The mixture was stirred at reflux at 110 °C for 1 h. Afterwards, the reaction mixture was cooled down, and 350 mL of hexane was added. After the end of precipitation, the solution was filtered off from the precipitate and evaporated in vacuo. The yield of epoxide (1*S*,5*S*,6*R*)*-***4** was 574 mg (3.82 mmol, 92%).

(1*S*,5*S*,6*R*)-2-Methyl-5-(prop-1-en-2-yl)-7-oxabicyclo[4.1.0]hept-2-ene *(1S*,*5S*,*6R)*-**4** is described in [28].

(1*R*,2*S*,6*S*)-3-Methyl-6-(prop-1-en-2-yl)cyclohex-3-ene-1,2-diyl diacetate ((1*R*,2*S*,6*S*)-**6**).

Yield: 80%, ^1^H NMR (500 MHz, CDCl_3_): δ = 5.63–5.66 (m, 1H, H-4), 5.53 (dd, 1H, J_1,2_ = 4.0 Hz, J_1,6a_ = 1.2 Hz, H-1), 5.53–5.48 (m, 1H, H-2), 4.84–4.81 (m, 1H, H-8′), 4.72 (br. S, 1H, H-8), 2.39 (br. D, 1H, J_6a,5a_ = 12.7 Hz, H-6a), 2.38–2.31 (m, 1H, H-5a), 2.01 and 2.00 (2s, 6H, 3H-12 and 3H-14), 2.06–1.96 (m, 1H, H-5e), 1.74 (br. S, 3H, H-9), 1.61 (m, 3H, H-10); ^13^C NMR (125 MHz, CDCl_3_): δ = 170.7 and 170.5 (2s, 2C, C-11 and C-13), 144.1 (s, C-7), 129.6 (s, C-3), 125.2 (d, C-4), 111.4 (t, C-8), 71.8 (d, C-2), 68.1 (d, C-1), 42.8 (d, C-6), 25.3 (t, C-5), 22.0 (q, C-9), 20.7 and 20.7 (2q, 2C, C-12 and C-14), 18.5 (q, C-10).

(1*S*,2*R*,6*S*)-3-Methyl-6-(prop-1-en-2-yl)cyclohex-3-ene-1,2-diyl diacetate ((1*S*,2*R*,6*S*)-**6**).

Yield: 77%, ^1^H NMR (500 MHz, CDCl_3_): δ = 5.62–5.66 (m; 1H, H-4), 5.45 (br. D; 1H, J_2e,1a_ = 3.9 Hz, H-2e), 5.05 (dd; 1H, J_1a,6a_ = 12.0 Hz, J_1a,2e_ = 3.9 Hz, H-1a), 4.81–4.78 (m; 1H, all J < 2.0 Hz, H-8′), 4.78–4.76 (m; 1H, all J < 2.0 Hz, H-8), 2.72 (ddd; 1H, J_6a,1a_ = 12.0 Hz, J_6a,5a_ = 11.0 Hz, J_6a,5e_ = 5.8 Hz, H-6a), 2.22 (dm; 1H, ^2^J = 18.1 Hz, H-5e), 2.09 (s, 3H, H-14), 2.15–2.06 (m, 1H, H-5a), 1.94 (s, 3H, H-12), 1.67–1.64 (m, 3H, H-10), 1.64–1.62 (m, 3H, H-9); ^13^C-NMR (125 MHz, CDCl_3_): δ = 170.7 (s, C-13), 170.3 (s, C-11), 144.3 (s, C-7), 130.0 (s, C-3), 127.0 (d, C-4), 112.8 (t, C-8), 71.2 (d, C-1), 69.3 (d, C-2), 40.3 (d, C-6), 30.8 (t, C-5), 20.8 (q, C-14), 20.7 (q, C-12), 20.2 (q, C-10), 19.1 (q, C-9).

(1*S*,2*S*,6*S*)-3-Methyl-6-(prop-1-en-2-yl)cyclohex-3-ene-1,2-diyl diacetate ((1*S*,2*S*,6*S*)-**6**).

Yield: 95%, ^1^H NMR (500 MHz, CDCl_3_): δ = 5.58–5.50 (m, 2H, H-2a and H-4), 5.17 (dd, 1H, J_1a,6a_ = 12.0 Hz, J_1a,2a_ = 7.8 Hz, H-1a), 4.79–4.75 (m, 2H, 2H-8), 2.61 (ddd, 1H, J_6a,1a_ = 12.0 Hz, J_6a,5a_ = 11.2 Hz, J_6a,5e_ = 5.6 Hz, H-6a), 2.19 (ddm, 1H, ^2^J = 18.1 Hz, J_5a,6a_ = 11.2 Hz, H-5a), 2.14–2.05 (m, 1H, H-5e), 2.02 (s, 3H, H-14), 1.95 (s, 3H, H-12), 1.66–1.63 (m, 3H, H-9), 1.60–1.56 (m, 3H, H-10); ^13^C NMR (125 MHz, CDCl_3_): δ = 170.8 (s, C-13), 170.3 (s, C-11), 143.7 (s, C-7), 131.2 (s, C-3), 124.7 (d, C-4), 113.5 (t, C-8), 75.4 (d, C-2), 73.5 (d, C-1), 46.3 (d, C-6), 29.8 (t, C-5), 20.7 (q, C-14), 20.7 (q, C-12), 18.8 (q, C-9), 18.3 (q, C-10).

(1*S*,5*S*,6*S*)-6-Hydroxy-2-methyl-5-(prop-1-en-2-yl)cyclohex-2-enyl acetate ((1*S*,5*S*,6*S*)-**9**) and (1*S*,2*S*,6*S*)-2-Hydroxy-3-methyl-6-(prop-1-en-2-yl)cyclohex-3-enyl acetate ((1*S*,2*S*,6*S*)-**8**) (2:1).

Total yield: 23% (1*S*,5*S*,6*S*)-9. ^1^H NMR (500 MHz, CDCl_3_): δ = 5.54–5.50 (m, 1H, H-4), 5.46–5.40 (m, 1H, H-2a), 4.93–4.91 (m, 1H, H-8′), 4.89–4.87 (m, 1H, H-8), 3.75 (dd, 1H, J_1a,6a_ = 11.4 Hz, J_1a,2a_ = 7.7 Hz, H-1a), 2.27 (ddd, 1H, J_6a,1a_ = 11.4 Hz, J_6a,5a_ = 11.1 Hz, J_6a,5e_ = 5.7 Hz, H-6a), 2.13 (s, 3H, H-12), 2.23–2.07 (m, 2H, 2H-5), 1.73 (m, 3H, all J < 2.0 Hz, H-9), 1.62–1.59 (m, 3H, H-10); ^13^C NMR (125 MHz, CDCl_3_): δ = 171.8 (s, C-11), 144.6 (s, C-7), 131.4 (s, C-3), 124.9 (d, C-4), 114.0 (t, C-8), 78.6 (d, C-2), 72.2 (d, C-1), 48.8 (d, C-6), 29.8 (t, C-5), 20.9 (q, C-12), 18.5 (2q, 2C, C-9, and C-10).

(1*S*,5*S*,6*S*)-8. ^1^H NMR (500 MHz, CDCl_3_): δ = 5.46–5.40 (m, 1H, H-4), 4.98 (dd, 1H, J_1a,6a_ = 11.4 Hz, J_1a,2a_ = 7.7 Hz, H-1a), 4.80–4.78 (m, 1H, H-8′), 4.78–4.76 (m, 1H, H-8), 4.09 (br. d, 1H, J_2a,1a_ = 6.7 Hz, H-2a), 2.27 (ddd, 1H, J_6a,1a_ = 11.4 Hz, J_6a,5a_ = 11.1 Hz, J_6a,5e_ = 5.7 Hz, H-6a), 2.23–2.07 (m, 2H, 2H-5), 2.05 (s, 3H, H-12), 1.74 (m, 3H, all J < 2.0 Hz, H-9), 1.67–1.65 (m, 3H, H-10); ^13^C NMR (125 MHz, CDCl_3_): δ = 171.9 (s, C-11), 144.4 (s, C-7), 134.4 (s, C-3), 122.5 (d, C-4), 113.7 (t, C-8), 77.5 (d, C-1), 74.7 (d, C-2), 46.0 (d, C-6), 30.0 (t, C-5), 20.9 (q, C-12), 19.0 (q, C-9), 18.6 (q, C-10).

(1*S*,5*R*,6*R*)-2-Methyl-5-((*S*)-2-methyloxiran-2-yl)-7-oxabicyclo[4.1.0]hept-2-ene (**13**).

Yield: 62%, ^1^H NMR (500 MHz, CDCl_3_): δ = 5.61 (dm, 1H, J_4,5e_ = 7.0 Hz, H-4), 3.39 (dm, J_1,2_ = 4.4 Hz, H-1), 3.08 (dd, J_2,1_ = 4.4 Hz, J_2,4_ = 2.3 Hz, H-2), 2.84 (br. d, 1H, ^2^J = 4.7 Hz, H-8′), 2.63 (br. d, 1H, ^2^J = 4.7 Hz, H-8), 2.05 (dm, 1H, ^2^J = 16.1 Hz, H-5e), 1.94 (dm, 1H, ^2^J = 16.1 Hz, H-5a), 1.89–1.85 (m, 3H, H-10), 1.81 (br. dd, J(6a,5a) = 11.9 Hz, J_6a,5e_ = 6.2 Hz, H-6a), 1.37 (s, 3H, H-9); ^13^C NMR (125 MHz, CDCl_3_): δ = 130.5 (s, C-3), 124.7 (d, C-4), 58.4 (s, C-7), 55.5 (d, C-1), 52.7 (t, C-8), 50.9 (d, C-2), 39.2 (d, C-6), 23.4 (t, C-5), 21.4 (q, C-10), 19.5 (q, C-9).

Interaction of (1*S*,5*S*,6*R*)-2-methyl-5-(prop-1-en-2-yl)-7-oxabicyclo[4.1.0]hept-2-ene (1*S*,5*S*,6*R*)-**4** with alcohols. General procedure.

0.03 mmol *p*-Toluenesulfonic acid (*p*-TsOH × H_2_O) was added to a solution of (1*S*,5*S*,6*R*)-2-methyl-5-(prop-1-en-2-yl)-7-oxabicyclo[4.1.0]hept-2-ene (1*S*,5*S*,6*R*)-**4** (0.7 mmol) and appropriate alcohol (0.75 mmol) in toluene. The reaction mixture was stirred for 1 h at RT. Then, *p*-TsOH × H_2_O was filtered off, and the solvent was evaporated in vacuo. The residue was separated by column chromatography on silica gel (20 g) using 0–100% EtOAc gradient in hexane as an eluent.

(1*R*,2*R*,6*S*)-2-Methoxy-3-methyl-6-(prop-1-en-2-yl)cyclohex-3-enol (**14**).

Yield: 33%, ${\left[\alpha \right]}_{D}^{26.0}$-26.8 (*c* 0.38, CHCl_3_), ^1^H NMR (500 MHz, CDCl_3_): δ = 5.65–5.61 (m, 1H, H-4), 4.96 (m, 1H, all J < 2.2 Hz, H-8′), 4.84 (br. s, 1H, H-8), 4.04–4.01 (m, 1H, H-1e), 3.48 (s, 3H, H-11), 3.39 (m, 1H, all J < 3.0 Hz, H-2e), 2.36 (br. dd, J_6a,5a_ 11.6, J_6a,5e_ = 5.2 Hz, H-6a), 2.16 (ddm, 1H, ^2^J = 17.8 Hz, J_5a,6a_ = 11.6 Hz, H-5a), 1.95 (dddq, 1H, ^2^J = 17.8 Hz, J_5e,4_ = J_5e,6a_ = 5.2 Hz, J_5e,10_ = 1.5 Hz, H-5e), 1.81 (br. s, 3H, H-9), 1.78 (m, 3H, all J ≤ 2.5 Hz, H-10); ^13^C NMR (125 MHz, CDCl_3_): δ = 145.9 (s, C-7), 130.5 (s, C-3), 125.2 (d, C-4), 111.1 (t, C-8), 81.2 (d, C-2), 66.9 (d, C-1), 58.4 (q, C-11), 40.0 (d, C-6), 24.1 (t, C-5), 22.5 (q, C-9), 20.9 (q, C-10); HRMS: 182.1299 (*M^+^*, C_11_H_18_O_2_^+^; calc. 182.1302).

(1*S*,4*R*,6*S*)-4-Methoxy-3-methyl-6-(prop-1-en-2-yl)cyclohex-2-enol (**15**)

Yield: 10%, ${\left[\alpha \right]}_{D}^{26.0}$+167 (*c* 0.22, CHCl_3_), ^1^H NMR (500MHz, CDCl_3_): δ = 5.76 (dq, 1H, J_2,1_ = 5.4 Hz, J_2,10_ = 1.5 Hz, H-2), 5.02 (m, 1H, all J < 2.2 Hz, H-8′), 4.79 (m, 1H, all J < 2.2 Hz, H-8), 4.09–4.06 (m, 1H, H-1), 3.51 (dd, 1H, J_4e,5a_ = 3.7 Hz, J_4e,5e_ = 2.0 Hz, H-4e), 3.37 (s, 3H, H-11), 2.44 (dm, 1H, J_6a,5a_ = 13.0 Hz, H-6a), 1.86 (dddd, 1H, ^2^J = 13.9 Hz, J_5e,6a_ = 2.8 Hz, J_5e,4e_ = 2.0 Hz, J_5e,1e_ = 0.6 Hz, H-5e), 1.81 (br. s, 3H, H-9), 1.80 (m, 3H, all J ≤ 2.5 Hz, H-10), 1.82–1.74 (m, 1H, H-5a); ^13^C NMR (125 MHz, CDCl_3_): δ = 146.0 (s, C-7), 137.8 (s, C-3), 125.8 (d, C-2), 111.9 (t, C-8), 77.5 (d, C-4), 63.2 (d, C-1), 57.5 (q, C-11), 40.3 (d, C-6), 24.4 (t, C-5), 22.5 (q, C-9), 21.0 (q, C-10); HR-MS: 182.1300 (*M^+^*, C_11_H_18_O_2_^+^; calc. 182.1302).

(1*R*,2*R*,6*S*)-2-Ethoxy-3-methyl-6-(prop-1-en-2-yl)cyclohex-3-enol (**16**)

Yield: 26%, ${\left[\alpha \right]}_{D}^{26.0}$-37.8 (*c* 0.23, CHCl_3_), ^1^H NMR (CDCl_3_, 600 MHz) *δ*: 1.22 (t, 3H, J_12,11_ = 7.0 Hz, H-12), 1.53 (br.s, 1H, -OH), 1.76–1.78 (m, 3H, H-10), 1.81 (br.s, 3H, H-9), 1.95 (dm, 1H, ^2^J = 17.8 Hz, H-5e), 2.12–2.20 (ddm, 1H, ^2^J = 17.8 Hz, J_5a,6a_ = 11.7 Hz, H-5a), 2.39 (br. dd, 1H, J_6a,5a_ = 11.7 Hz, J_6a,5e_ = 5.3 Hz, H-6a), 3.47–3.49 (m, 1H, H-2), 3.58 (dq, 1H, ^2^J = 9.5 Hz, J_11,12_ = 7.0 Hz, H-11), 3.74 (dq, 1H, ^2^J = 9.5 Hz, J_11′,12_ = 7.0 Hz, H-11′), 3.97–4.00 (m, 1H, H-1e), 4.84 (br. s, 1H, H-8), 4.94–4.96 (m, 1H, H-8′), 5.61–5.65 (m, 1H, H-4); ^13C NMR (CDCl^_3_, 150 MHz) *δ*: 67.6 (d, C-1), 79.3 (d, C-2), 130.5 (s, C-3), 125.1 (d, C-4), 24.0 (t, C-5), 40.0 (d, C-6), 146.0 (s, C-7), 111.0 (t, C-8), 22.5 (q, C-9), 20.9 (q, C-10), 66.1 (t, C-11), 15.7 (q, C-12); HR-MS: 196.1455 (*M^+^*, C_12_H_20_O_2_^+^; calc. 196.1458).

(1*S*,4*R*,6*S*)-4-Ethoxy-3-methyl-6-(prop-1-en-2-yl)cyclohex-2-enol (**17**)

Yield: 16%,${\left[\alpha \right]}_{D}^{26.0}$+78.8 (*c* 0.19, CHCl_3_), ^1^H NMR (600 MHz, CDCl_3_): δ = 5.77 (dq, 1H, J_2,1_ = 5.3 Hz, J_2,10_ = 1.6 Hz, H-2), 5.03–5.00 (m, 1H, H-8′), 4.79 (br. s, 1H, H-8), 4.08 (br. dd, J_1,2_ = 5.3 Hz, J_1,6a_ = 3.8 Hz, H-1), 3.65 (dq, 1H, ^2^J = 9.3 Hz, J_11′,12_ = 7.0 Hz, H-11′), 3.63–3.60 (m, 1H, H-4e), 3.41 (dq, 1H, ^2^J = 9.3 Hz, J_11,12_ = 7.0 Hz, H-11), 2.48 (dm, 1H, J_6a,5a_ = 12.0 Hz, H-6a), 1.81 (br.s, 3H, H-9), 1.80 (br.s, 3H, H-10), 1.85–1.75 (m, 2H, 2H-5), 1.19 (t, 3H, J_12,11_ = 7.0 Hz, H-12); ^13^C NMR (150 MHz, CDCl_3_): δ = 146.1 (s, C-7), 137.9 (s, C-3), 125.7 (d, C-2), 111.9 (t, C-8), 75.6 (d, C-4), 65.1 (t, C-11), 63.2 (d, C-1), 40.3 (d, C-6), 25.3 (t, C-5), 22.5 (q, C-9), 21.0 (q, C-10), 15.5 (q, C-12); HR-MS: 196.1454 (*M^+^*, C_12_H_20_O_2_^+^; calc. 196.1458).

(1*R*,2*R*,6*S*)-3-Methyl-2-(pentyloxy)-6-(prop-1-en-2-yl)cyclohex-3-enol (**18**)

Yield: 27%, ${\left[\alpha \right]}_{D}^{25.0}$-5.2 (*c* 0.31, CHCl_3_), ^1^H NMR (600 MHz, CDCl_3_): δ = 5.66–5.62 (m, 1H, H-4), 4.98–4.95 (m, 1H, H-8′), 4.84 (br. s, 1H, H-8), 4.01–3.98 (m, 1H, H-1), 3.66 (dt, 1H, ^2^J = 9.3 Hz, J_11′,12_ = 6.3 Hz, H-11′), 3.54 (dt, 1H, ^2^J = 9.3 Hz, J_11,12_ = 6.3 Hz, H-11), 3.48–3.46 (m, 1H, H-2), 2.40 (br. dd, 1H, J_6a,5a_ = 12.7 Hz, J_6a,5e_ = 5.2 Hz, H-6a), 2.13–2.22 (m, 1H, H-5a), 1.95 (dm, 1H, ^2^J = 17.8 Hz, H-5e), 1.81 (br.s, 3H, H-9), 1.79–1.77 (m, 3H, H-10), 1.63–1.54 (m, 2H, 2H-12), 1.48 (br.d, 1H, J_1-OH,H-1_ = 3.7 Hz, 1-OH), 1.39–1.26 (m, 4H, 2H-13, 2H-14), 0.88 (t, 3H, J_15,14_ = 7.1 Hz, H-15); ^13C NMR (150 MHz, CDCl^_3_): δ = 146.0 (s, C-7), 130.6 (s, C-3), 125.1 (d, C-4), 111.1 (t, C-8), 79.5 (d, C-2), 71.0 (t, C-11), 67.4 (d, C-1), 40.0 (d, C-6), 29.8 (t, C-12), 28.3 (t, C-13), 24.0 (t, C-5), 22.5 (q, C-9), 22.4 (t, C-14), 21.0 (q, C-10), 13.9 (q, C-15); HR-MS: 238.1926 (*M^+^*, C_15_H_26_O_2_^+^; calc. 238.1927).

(1*R*,2*R*,6*S*)-3-Methyl-2-phenethoxy-6-(prop-1-en-2-yl)cyclohex-3-enol (**19**)

Yield: 20%, ${\left[\alpha \right]}_{D}^{25.0}$-24.5 (*c* 0.98, CHCl_3_), ^1^H NMR (600 MHz, CDCl_3_): δ = 7.29–7.24 (m, 2H, H-15, H-17), 7.24–7.21 (m, 2H, H-14, H-18), 7.18 (tm, 1H, J_16,15_ = J_16,17_ = 7.2 Hz, H-16), 5.65–5.61 (m, 1H, H-4), 4.93–4.95 (m, 1H, H-8′), 4.81 (br. s, 1H, H-8), 3.93–3.90 (m, 1H, H-1), 3.88 (dt, 1H, ^2^J = 9.4 Hz, J_11,12_ = 7.2 Hz, H-11′), 3.80 (dt, 1H, ^2^J = 9.4 Hz, J_11,12_ = 6.8 Hz, H-11), 3.51–3.48 (m, 1H, H-2), 2.93–2.87 (m, 2H, 2H-12), 2.34 (br. dd, 1H, J_6a,5a_ = 11.7 Hz, J_6a,5e_ = 5.3 Hz, H-6a), 2.20–2.11 (m, 1H, H-5a), 1.94 (dm, 1H, ^2^J = 17.8 Hz, H-5e), 1.76–1.73 (m, 3H, H-10), 1.71 (br.s, 3H, H-9), 1.47 (d, 1H, J_1-OH,H-1_ = 4.0 Hz, 1-OH); ^13^C NMR (150 MHz, CDCl_3_): δ = 145.9 (s, C-7), 138.8 (s, C-13), 130.4 (s, C-3), 128.8 (2d, C-14, C-18), 128.2 (2d, C-15, C-17), 126.1 (d, C-16), 125.2 (d, C-4), 111.0 (t, C-8), 79.8 (d, C-2), 71.8 (t, C-11), 67.4 (d, C-1), 39.9 (d, C-6), 36.7 (t, C-12), 24.0 (t, C-5), 22.4 (q, C-9), 20.9 (q, C-10); HR-MS: 272.1774 (*M^+^*, C_18_H_24_O_2_^+^; calc. 272.1771).

(1*R*,2*R*,6*S*)-3-Methyl-6-(prop-1-en-2-yl)-2-(((*S*)-4-(prop-1-en-2-yl)cyclohex-1-enyl)methoxy)cyclohex-3-enol (**20**)

Yield: 22%, **20-maj** and **20-min** ^1^H NMR (500 MHz, CDCl_3_): δ = 5.76–5.71 (m, 1H, H-13), 5.66–5.61 (m, 1H, H-4), 4.98–4.94 (m, 1H, H-8′), 4.86–4.83 (m, 1H, H-8), 4.73–4.66 (m, 2H, H-19), 3.54–3.50 (m, 1H, H-2), 2.43 (br. dd, 1H, J_6a,5a_ = 11.7 Hz, J_6a,5e_ = 5.0 Hz, H-6a), 2.25–2.04 (m, 5H, H-5a, H-14′, H-15, 2H-17), 2.02–1.90 (m, 2H, H-5e, H-14), 1.88–1.80 (m, 4H, 3H-9, H-16′), 1.78–1.75 (m, 3H, H-10), 1.73–1.70 (m, 3H, H-20), 1.52–1.41 (m, 1H, H-16); **20-maj** ^13^C NMR (125 MHz, CDCl_3_): δ = 149.7 (s, C-18), 146.0 (s, C-7), 134.7 (s, C-12), 130.5 (s, C-3), 125.3 (d, C-13), 125.3 (d, C-4), 111.1 (t, C-8), 108.5 (t, C-19), 78.6 (d, C-2), 75.1 (t, C-11), 67.4 (d, C-1), 40.9 (d, C-15), 40.0 (d, C-6), 30.4 (t, C-14), 27.4 (t, C-16), 26.6 (t, C-17), 24.1 (t, C-5), 22.5 (q, C-9), 21.1 (q, C-10), 20.7 (q, C-20); **20-min**. Some different signals: ^13^C NMR (125 MHz, CDCl_3_): δ = 125.1 (d, C-13′), 79.0 (d, C-2′), 75.6 (t, C-11′), 67.5 (d, C-1′), 40.9 (d, C-15′), 40.0 (d, C-6′), 26.7 (t, C-17′), 24.1 (t, C-5′); HR-MS: 302.2228 (*M^+^*, C_20_H_30_O_2_^+^; calc. 302.2240).

(1*R*,2*R*,6*S*)-3-Methyl-2-(4-methylbenzyloxy)-6-(prop-1-en-2-yl)cyclohex-3-enol (**21**)

Yield: 30%, ${\left[\alpha \right]}_{D}^{25.0}$-31.1 (*c* 0.80, CHCl_3_), ^1^H NMR (600 MHz, CDCl_3_): δ = 7.24 (dm, 2H, J_13,14_ = J_17,16_ = 7.8 Hz, H-13, H-17), 7.14 (dm, 2H, J_14,13_ = J_16,17_ = 7.8 Hz, H-14, H-16), 5.66–5.62 (m, 1H, H-4), 4.98–4.95 (m, 1H, H-8′), 4.84 (br. s, 1H, H-8), 4.67 (d, 1H, ^2^J = 11.5 Hz, H-11′), 4.58 (d, 1H, ^2^J = 11.5 Hz, H-11), 3.64–3.61 (m, 1H, H-2), 2.45 (br. dd, 1H, J_6a,5a_ = 11.7 Hz, J_6a,5e_ = 5.2 Hz, H-6a), 2.32 (s, 3H, H-18), 2.22–2.13 (m, 1H, H-5a), 1.96 (dm, 1H, ^2^J = 17.8 Hz, H-5e), 1.79 (br.s, 3H, H-9), 1.73–1.71 (m, 3H, H-10), 1.48 (d, 1H, J_1-OH,H-1_ = 4.0 Hz, 1-OH); ^13^C NMR (150 MHz, CDCl_3_): δ = 146.0 (s, C-7), 137.4 (s, C-15), 135.3 (s, C-12), 130.4 (s, C-3), 129.0 (2d, C-14, C-16), 128.1 (2d, C-13, C-17), 125.3 (d, C-4), 111.1 (t, C-8), 79.0 (d, C-2), 72.7 (t, C-11), 67.4 (d, C-1), 40.0 (d, C-6), 24.1 (t, C-5), 22.5 (q, C-9), 21.1 (q, C-18), 20.9 (q, C-10); HR-MS: 272.1766 (*M^+^*, C_15_H_26_O_2_^+^; calc. 272.1771).

(1*R*,2*R*,6*S*)-2-(Furan-2-ylmethoxy)-3-methyl-6-(prop-1-en-2-yl)cyclohex-3-enol (**22**)

Yield: 17%, ${\left[\alpha \right]}_{D}^{25.0}$-31.0 (*c* 0.64, CHCl_3_), ^1^H NMR (500 MHz, CDCl_3_): δ = 7.39 (dd, 1H, J_15,14_ = 1.8, J_15,13_ = 1.0 Hz, H-15), 6.33 (br. d, 1H, J_13,14_ = 3.2 Hz, H-13), 6.32 (dd, 1H, J_14,13_ = 3.2 Hz, J_14,15_ = 1.8 Hz, H-14), 5.64–5.61 (m, 1H, H-4), 4.97–4.94 (m, 1H, H-8′), 4.85–4.82 (br. s, 1H, H-8), 4.61 (d, 1H, ^2^J = 13.0 Hz, H-11′), 4.57 (d, 1H, ^2^J = 13.0 Hz, H-11), 3.96–3.92 (m, 1H, H-1), 3.65–3.62 (m, 1H, H-2), 2.41 (br. dd, 1H, J_6a,5a_ = 11.7 Hz, J_6a,5e_ = 5.3 Hz, H-6a), 2.21–2.11 (m, 1H, H-5a), 1.95 (dm, 1H, ^2^J = 17.8 Hz, H-5e), 1.80 (br.s, 3H, H-9), 1.68–1.64 (m, 3H, H-10), 1.50 (br. s, 1-OH); ^13^C NMR (125 MHz, CDCl_3_): δ = 151.9 (s, C-12), 145.9 (s, C-7), 142.6 (d, C-15), 130.3 (s, C-3), 125.5 (d, C-4), 111.0 (t, C-8), 110.2 (d, C-14), 109.3 (d, C-13), 78.7 (d, C-2), 67.6 (d, C-1), 64.4 (t, C-11), 39.9 (d, C-6), 24.1 (t, C-5), 22.4 (q, C-9), 20.6 (q, C-10); HR-MS: 248.1402 (*M^+^*, C_15_H_20_O_3_^+^; calc. 248.1407).

(1*R*,2*R*,6*S*)-3-Methyl-6-(prop-1-en-2-yl)-2-(thiophen-2-ylmethoxy)cyclohex-3-enol (**23**).

Yield: 26%, ${\left[\alpha \right]}_{D}^{25.0}$-26.2 (*c* 0.9, CHCl_3_), ^1^H NMR (500 MHz, CDCl_3_): δ = 7.27 (dd, 1H, J_15,14_ = 5.1 Hz, J_15,13_ = 1.2 Hz, H-15), 7.02 (br. d, 1H, J_13,14_ = 3.4 Hz, H-13), 6.95 (dd, 1H, J_14,15_ = 5.1 Hz, J_14,13_ = 3.4 Hz, H-14), 5.67–5.63 (m, 1H, H-4), 4.98–4.95 (m, 1H, H-8′), 4.85 (d, 1H, ^2^J = 12.5 Hz, H-11′), 4.84 (br. s, 1H, H-8), 4.79 (d, 1H, ^2^J = 12.5 Hz, H-11), 4.02–3.99 (m, 1H, H-1), 3.68–3.66 (m, 1H, H-2), 2.45 (br. dd, 1H, J_6a,5a_ = 11.7 Hz, J_6a,5e_ = 5.3 Hz, H-6a), 2.22–2.14 (m, 1H, H-5a), 1.97 (dm, 1H, ^2^J = 17.8 Hz, H-5e), 1.80 (br. s, 3H, H-9), 1.73–1.70 (m, 3H, H-10), 1.51 (br. d, 1H, J_1-OH,H-1_ = 3.5 Hz, 1-OH); ^13^C NMR (125 MHz, CDCl_3_): δ = 145.9 (s, C-7), 141.2 (s, C-12), 130.2 (s, C-3), 125.9 (d, C-15), 125.6 (d, C-4), 126.5 (d, C-14), 126.4 (d, C-13), 111.1 (t, C-8), 78.8 (d, C-2), 67.5 (d, C-1), 67.0 (t, C-11), 39.9 (d, C-6), 24.1 (t, C-5), 22.5 (q, C-9), 20.8 (q, C-10); HR-MS: 264.1173 (*M^+^*, C_15_H_20_O_2_S^+^; calc. 264.1179).

(1*R*,2*R*,6*S*)-2-((*E*)-Hex-2-enyloxy)-3-methyl-6-(prop-1-en-2-yl)cyclohex-3-enol (**24**).

Yield: 27%, ${\left[\alpha \right]}_{D}^{26.0}$-36.0 (*c* 0.1, CHCl_3_), ^1^H NMR (600 MHz, CDCl_3_): δ = 5.70 (dm, 1H, J_13,12_ = 15.4 Hz, H-13), 5.65–5.61 (m, 1H, H-4), 5.57 (dm, 1H, J_12,13_ = 15.4 Hz, H-12), 4.97–4.94 (m, 1H, H-8′), 4.84 (br. s, 1H, H-8), 4.13 (ddm, 1H, ^2^J = 11.8 Hz, J_11′,12_ = 6.0 Hz, H-11′), 4.01 (ddm, 1H, ^2^J = 11.8 Hz, J_11,12_ = 6.8 Hz, H-11), 4.01–3.97 (m, 1H, H-1), 3.55–3.53 (m, 1H, H-2), 2.41 (br. dd, 1H, J_6a,5a_ = 11.6 Hz, J_6a,5e_ = 5.2 Hz, H-6a), 2.20–2.12 (m, 1H, H-5a), 2.04–1.98 (m, 2H, 2H-14), 1.95 (dm, 1H, ^2^J = 17.8 Hz, H-5e), 1.81 (br. s, 3H, H-9), 1.77–1.74 (m, 3H, H-10), 1.48 (br.d, 1H, J_1-OH,H-1_ = 4.1 Hz, 1-OH), 1.43–1.35 (m, 2H, 2H-15), 0.88 (t, 3H, J_16,15_ = 7.4 Hz, H-16); ^13^C NMR (150 MHz, CDCl_3_): δ = 146.1 (s, C-7), 134.8 (d, C-13), 130.6 (s, C-3), 126.7 (d, C-12), 125.2 (d, C-4), 111.0 (t, C-8), 78.4 (d, C-2), 71.4 (t, C-11), 67.7 (d, C-1), 40.0 (d, C-6), 34.2 (t, C-14), 24.1 (t, C-5), 22.5 (q, C-9), 22.1 (t, C-15), 20.9 (q, C-10), 13.6 (q, C-16); HR-MS: 250.1934 (*M^+^*, C_16_H_26_O_2_^+^; calc. 250.1927).

(1*R*,2*R*,6*S*)-2-(((1*R*,5*S*)-6,6-Dimethylbicyclo[3.1.1]hept-2-en-2-yl)methoxy)-3-methyl-6-(prop-1-en-2-yl)cyclohex-3-enol (**25**).

Yield: 30%, **25-maj** ^1^H NMR (600 MHz, CDCl_3_): δ = 5.66–5.60 (m, 1H, H-4), 5.53–5.49 (m, 1H, H-13), 4.97–4.94 (m,1H, H-8”), 4.85–4.83 (m, 1H, H-8), 4.01–3.94 (m, 3H, 2H-11, H-1), 3.56–3.54 (m, 1H, H-2), 2.44–2.35 (m, 2H, H-18*sin*, H-6a), 2.34–2.26 (m, 1H, H-14′), 2.24–2.12 (m, 3H, H-5a, H-14, H-17), 2.10–2.05 (m, 1H, H-15), 1.99–1.91 (m, 1H, H-5e), 1.82 (s, 3H, H-9), 1.73–1.76 (m, 3H, H-10), 1.45 (d, 1H, J_1-OH,H-1_ = 4.0 Hz, 1-OH), 1.27 (s, 3H, H-19), 1.21 (d, 1H, ^2^J = 8.7 Hz, H-18*anti*), 0.81 (s, 3H, H-20); ^13^C NMR (150 MHz, CDCl_3_): δ = 146.1 (s, C-7), 145.4 (s, C-12), 130.8 (s, C-3), 125.2 (d, C-4), 120.5 (d, C-13), 111.0 (t, C-8), 77.9 (d, C-2), 73.1 (t, C-11), 67.2 (d, C-1), 43.5 (d, C-17), 40.7 (d, C-15), 40.0 (d, C-6), 37.9 (s, C-16), 31.5 (t, C-18), 31.2 (t, C-14), 26.1 (q, C-19), 24.2 (t, C-5), 22.5 (q, C-9), 21.1 (q, C-10), 20.9 (q, C-20); HR-MS: 302.2242 (*M^+^*, C_20_H_30_O_2_^+^; calc. 302.2240).

**25-min** ^1^H NMR (600 MHz, CDCl_3_): δ = 5.66–5.60 (m, 1H, H-4′), 5.53–5.49 (m, 1H, H-13′), 4.97–4.94 (m, 1H, H-8′), 4.84–4.82 (m, 1H, H-8′), 4.01–3.94 (m, 3H, 2H-11′, H-1′), 3.53–3.51 (m, 1H, H-2′), 2.44–2.35 (m, 2H, H-18′*sin*, H-6a), 2.34–2.26 (m, 1H, H-14′), 2.24–2.12 (m, 3H, H-5′a, H-14′, H-17′), 2.10–2.05 (m, 1H, H-15′), 1.99–1.91 (m, 1H, H-5′e), 1.81 (s, 3H, H-9′), 1.79–1.77 (m, 3H, H-10′), 1.45 (d, 1H, J_1-OH,H-1_ = 4.0 Hz, 1-OH), 1.27 (s, 3H, H-19′), 1.20 (d, 1H, ^2^J = 8.7 Hz, H-18′*anti*), 0.82 (s, 3H, H-20′); ^13^C NMR (150 MHz, CDCl_3_): δ = 146.1 (s, C-7′), 145.7 (s, C-12′), 130.9 (s, C-3′), 125.0 (d, C-4′), 119.6 (d, C-13′), 111.0 (t, C-8′), 79.5 (d, C-2′), 74.1 (t, C-11′), 67.8 (d, C-1′), 43.6 (d, C-17′), 40.8 (d, C-15′), 40.0 (d, C-6′), 37.1 (s, C-16′), 31.4 (t, C-18′), 31.2 (t, C-14′), 26.2 (q, C-19′), 24.2 (t, C-5′), 22.5 (q, C-9′), 21.2 (q, C-10′), 21.0 (q, C-20′); HR-MS: 302.2242 (*M^+^*, C_20_H_30_O_2_^+^; calc. 302.2240).

Reaction of (1*S*,5*S*,6*R*)-2-methyl-5-(prop-1-en-2-yl)-7-oxabicyclo [4.1.0]hept-2-ene (1*S*,5*S*,6*R*)-**4** with thiols. General procedure.

An appropriate thiol (0.98 mmol) was added to sodium *tert*-butoxide or NaOH (0.98 mmol) dissolved in 10 mL methanol. After 5 min, a solution of (1*S*,5*S*,6*R*)-2-methyl-5-(prop-1-en-2-yl)-7-oxabicyclo[4.1.0]hept-2-ene (0.8 mmol) (1*S*,5*S*,6*R*)*-***4** in CH_3_OH (10 mL) was added to the mixture under stirring. The reaction mixture was stirred for 2 h at RT. The solution was then distilled off and the residue was extracted with ethyl acetate (3×10 mL). The organic phase was dried over Na_2_SO_4_, filtered, and evaporated in vacuo. The residue was separated by column chromatography on silica gel (20 g) using 0–100% EtOAc gradient in hexane as an eluent or 0–100% EtOH gradient in CHCl_3_.

(1*R*,2*R*,6*S*)-3-Methyl-6-(prop-1-en-2-yl)-2-(propylthio)cyclohex-3-enol (**26**)

Yield: 37%, ^1^H NMR (600 MHz, CDCl_3_): δ = 5.54–5.50 (m, 1H, H-4), 4.95 (m, 1H, one of H-8), 4.83 (s, 1H, one of H-8), 4.02 (m, 1H, H-1e), 3.08–3.05 (m, 1H, H-2e), 2.67–2.61 (m, 1H, H-6a), 2.61–2.58 (m, 1H, H-11′), 2.54–2.48 (m, 1H, H-11), 2.22–2.14 (br.d, 1H, J_5a,5e_ = 17.8 Hz, H-5a), 1.97 (d.m, 1H, J_5e,5a_ = 17.8 Hz, H-5e), 1.82 (s, 3H, H-9), 1.83 (s, 3H, H-7), 1.74 (br.s, 1H, -OH), 1.70 -1.56 (m, 2H, H-12), 0.98 (t, 3H, J = 7.3Hz, H-13); ^13^C NMR (150 MHz, CDCl_3_): δ = 146.1 (s, C-7), 129.8 (s, C-3), 123.7 (d, C-4), 110.9 (t, C-8), 71.0 (d, C-1), 51.4 (d, C-2), 38.8 (d, C-6), 34.9 (t, C-11), 24.0 (t, C-5), 23.3 (t, C-12), 22.3 (q, C-9), 22.0 (q, C-10), 13.3 (q, C-13); HR-MS: 226.1390 (*M^+^*, C_13_H_22_O_1_^32^S_1_^+^; calc. 226.1386).

(1*R*,2*R*,6*S*)-2-(Furan-2-ylmethylthio)-3-methyl-6-(prop-1-en-2-yl)cyclohex-3-enol (**27**)

Yield: 14%, ^1^H NMR(500 MHz, CDCl_3_): δ = 7.35 (dd, 1H, J_15,14_ = 1.9 Hz, J_15,13_ = 0.7 Hz, H-15), 6.30 (dd, 1H, J_14,13_ = 3.2 Hz, J_14,15_ = 1.9 Hz, H-14), 6.22 (dd, 1H, J_13,14_ = 3.2 Hz, J_13,15_ = 0.7 Hz, H-13), 5.56–5.51 (m, 1H, H-4), 4.93 –4.85 (m, 1H, H-8′), 4.82 (br. s, 1H, H-8), 3.88–3.86 (m, 1H, H-1e), 3.82 (d, 1H, ^2^J = 14.9 Hz, H-11′), 3.75 (d, 1H, ^2^J = 14.9 Hz, H-11), 3.14–3.11 (m, 1H, H-2e), 2.59 (br. dd, 1H, J_6a,5a_ = 11.9 Hz, J_6a,5e_ = 5.6 Hz, H-6a), 2.18 (ddm, 1H, ^2^J = 17.8 Hz, J_5a,6a_ = 11.9 Hz, H-5a), 1.97 (dm, 1H, ^2^J = 17.8 Hz, H-5e), 1.78 (br. s, 3H, H-9),1.72–1.75 (m, 3H, H-10); ^13^C NMR (125 MHz, CDCl_3_): δ = 151.7 (s, C-12), 146.0 (s, C-7), 142.0 (d, C-15), 129.3 (s, C-3), 124.4 (d, C-4), 110.9 (t, C-8), 110.4 (d, C-14), 107.5 (d, C-13), 70.6 (d, C-1), 51.4 (d, C-2), 38.9 (d, C-6), 29.4 (t, C-11), 27.7 (q, C-10), 24.0 (t, C-5), 22.3 (q, C-9).

(1*R*,2*R*,6*S*)-2-(2-Hydroxyethylthio)-3-methyl-6-(prop-1-en-2-yl)cyclohex-3-enol (**28**)

Yield: 75%,${\left[\alpha \right]}_{D}^{24}$-37.8(*c* 0.73, CHCl_3_), ^1^H NMR (600 MHz, CDCl_3_): δ = 5.56 (br.s, 1H, H-4); 4.96 (s, 1H, one of H-8), 4.84 (s, 1H, one of H-8), 4.03 (br.s, 1H, H-1), 3.76 (t, 2H, J = 6.14 Hz, H-12), 3.12 (br.s, 1H, H-2), 2.82 (dd, 1H, J_11,12_ = 13.8 Hz, J_11,11′_ =6.3 Hz, H-11′), 2.76 (dd, 1H, J_11,12_ = 13.8 Hz, J_11,11′_ = 6.3 Hz, H-11), 2.65–2.61 (dd, 1H, J_6a,1a_ = 11.75 Hz, J_6a,5_ = 5.5 Hz, H-4), 2.01 (br.s, 2H, H-11), 2.03–1.95 (m, 2H, H-5), 1.83 (s, 3H, H-10), 1.82 (s, 3H, H-9).^13^C NMR (150 MHz, CDCl_3_): δ = 145.8 (s, C-7), 129.2 (s, C-3), 124.5 (d, C-4), 111.2 (t, C-8), 71.0 (d, C-1), 61.0 (t, C-12), 51.3 (d, C-2), 38.7 (d, C-6), 35.8 (t, C-11), 24.0 (t, C-5), 22.3 (q, C-9), 22.0 (q, C-10); HR-MS: 228.1182 (*M^+^*, C_12_H_20_O_2_^32^S_1_^+^; calc. 228.1179).

(1*R*,2*R*,6*S*)-2-(4-Chlorophenylthio)-3-methyl-6-(prop-1-en-2-yl)cyclohex-3-enol (**29**).

Yield: 67%, ${\left[\alpha \right]}_{D}^{24}$-42.9 (*c* 3.86, CHCl_3_), ^1^H NMR (600 MHz, CDCl_3_): δ = 7.36 (br. d, 2H, J_12,13_ = 8.5 Hz, 2H-12), 7.26 (br. d, 2H, J_13,12_ = 8.5 Hz, 2H-13), 5.63–5.66 (m, 1H, H-4), 4.94–4.96 (m, 1H, H-8′), 4.82 (br. s, 1H, H-8), 3.97 (br. s, 1H, H-1e), 3.57 (br. s, 1H, H-2e), 2.69 (br. dd, 1H, J_6a,5a_ = 12.0 Hz, J_6a,5e_ = 5.5 Hz, H-6a), 2.19–2.26 (m, 1H, H-5a), 2.01 (dm, 1H, ^2^J = 17.8 Hz, H-5e), 1.90–1.92 (m, 3H, H-10), 1.77 (br. s, 3H, H-9); ^13^C NMR (150 MHz, CDCl_3_): *δ* = 145.6 (s, C-7), 134.2 (s, C-11), 133.1 (s, C-14), 132.4 (d, 2C-12), 129.1 (d, 2C-13), 128.4 (s, C-3), 125.5 (d, C-4), 111.2 (t, C-8), 69.7 (d, C-1), 54.7 (d, C-2), 38.7 (d, C-6), 24.0 (t, C-5), 22.4 (q, C-9), 22.1 (q, C-10). HR-MS: 294.0834 (*M^+^*, C_16_H_19_O_1_^35^Cl_1_^32^S_1_^+^; calc. 294.0840).

(1*R*,2*R*,6*S*)-2-(2-Aminophenylthio)-3-methyl-6-(prop-1-en-2-yl)cyclohex-3-enol (**30**)

Yield: 42%, ${\left[\alpha \right]}_{D}^{24}$+63.6 (*c* = 2.25 in CHCl_3_), ^1^H NMR (500 MHz, CDCl_3_): δ = 7.38 (dd, 1H, J_16,15_ = 7.7 Hz, J_16,14_ = 1.6 Hz, H-16), 7.12 (ddd, 1H, J_14,13_ = 8.0 Hz, J_14,15_ = 7.3 Hz, J_14,16_ = 1.6 Hz, H-14), 6.71 (dd, 1H, J_13,14_ = 8.0 Hz, J_13,15_ = 1.3 Hz, H-13), 6.67 (ddd, 1H, J_15,16_ = 7.7 Hz, J_15,14_ = 7.3 Hz, J_15,13_ = 1.3 Hz, H-15), 5.66–5.62 (m, 1H, H-4), 4.96–4.94 (m, 1H, H-8′), 4.83 (m, 1H, all J < 2.0 Hz, H-8), 3.97 (dd, 1H, J_1,2_ = 2.3 Hz, J_1,6a_ = 1.6 Hz, H-1e), 3.42–3.44 (m, 1H, H-2e), 2.84 (br. dd, 1H, J_6a,5a_ = 12.0 Hz, J_6a,5e_ = 5.4 Hz, H-6a), 2.25 (ddm, 1H, ^2^J = 17.9 Hz, J_5a,6a_ = 12.0 Hz, H-5a), 2.02 (dm, 1H, ^2^J = 17.9 Hz, H-5e), 1.98–1.95 (m, 3H, H-10), 1.84 (m, 3H, all J < 2.0 Hz, H-9); ^13^C NMR (125 MHz, CDCl_3_): δ = 22.1 (q, C-10), 22.4 (q, C-9), 24.3 (t, C-5), 38.4 (d, C-6), 54.3 (d, C-2), 69.6 (d, C-1), 111.0 (t, C-8), 114.9 (d, C-13), 117.1 (s, C-11), 118.4 (d, C-15), 125.4 (d, C-4), 128.9 (s, C-3), 130.2 (d, C-14), 136.7 (d, C-16), 146.0 (s, C-7), 148.4 (s, C-12).

(1*R*,2*R*,6*S*)-2-(6-Methoxy-1*H*-benzo[*d*]imidazol-2-ylthio)-3-methyl-6-(prop-1-en-2-yl)cyclohex-3-enol (**31**)

Yield: 32%, m.p. 84 °C with decomposition, ${\left[\alpha \right]}_{D}^{27}$+24.7 (*c* = 0.38 in EtOH), ^1^H NMR (600 MHz, CD_3_OD:CDCl_3_ (1:10 *v*/*v*)): δ = 1.71 (br. s, 3H, H-9), 1.80–1.83 (m, 3H, H-10), 2.02 (dm, 1H, ^2^J = 17.6 Hz, H-5e), 2.24–2.34 (m, 1H, H-5a), 2.59 (br. dd, 1H, J_6a,5a_ = 11.7 Hz, J_6a,5e_ = 5.3 Hz, H-6a), 3.76 (s, 3H, H-21), 4.30–4.34 (m, 2H, H-1e and H-2e), 4.80 (br. s, 1H, one of H-8), 4.84–4.87 (m, one of 1H, H-8′), 5.65–5.69 (m, 1H, H-4), 6.78 (dd, 1H, J_17,18_ = 8.8 Hz, J_17,15_ = 2.4 Hz, H-17), 6.96 (br. s, 1H, H-15), 7.35 (d, 1H, J_18,17_ = 8.8 Hz, H-18); ^13^C NMR (150 MHz, CDCl_3_): δ = 21.9 (q, C-10), 22.2 (q, C-9), 24.6 (t, C-5), 39.2 (d, C-6), 53.2 (d, C-2), 55.7 (q, C-21), 71.0 (d, C-1), 97.4 (d, C-15), 111.2 (t, C-8), 111.6 (d, C-17), 115.0 (d, C-18), 126.9 (d, C-4), 127.7 (s, C-3), 139.4 (s, C-19), 139.4 (s, C-14),145.7 (s, C-7), 148.5 (s, C-12), 156.3 (s, C-16); HR-MS: 330.1394 (*M^+^*, C_18_H_22_O_2_N_2_^32^S_1_^+^; calc. 330.1397).

(1*R*,2*R*,6*S*)-2-(Isopropylthio)-3-methyl-6-(prop-1-en-2-yl)cyclohex-3-enol (**32**)

Yield: 37%, ${\left[\alpha \right]}_{D}^{24}$-81.3(*c* = 0.77 in CHCl_3_). ^1^H NMR (600 MHz, CDCl_3_): δ = 1.27 (d, 3H, J = 6.8 Hz, H-13), 1.33 (d, 3H, J = 6.8 Hz, H-12), 1.82 (s, 3H, H-9), 1.83 (s, 3H, H-10), 1.94–2.01 (d.m, 1H, J_5e,5a_ = 17.9 Hz, H-5e), 2.13–2.21 (m,1H, H-5a), 2.65 (dd, 1H, J_6a,1a_ = 12 Hz, J_6a,5e_ = 5.6 Hz, H-6a), 3.01 (hept, 1H, J = 6.8 Hz, H-11), 3.09 (br.s, 1H, H-2e), 4.01–4.03 (m, 1H, H-1e), 4.83 (s, 1H, one of H-8), 4.95 (m, 1H, one of H-8), 5.49–5.52 (m, 1H, H-4); ^13^C NMR (150 MHz, CDCl_3_): δ = 21.9 (q, C-10), 22.3 (q, C-9), 23.6 (q, C-12), 23.9 (t, C-5), 24.0 (q, C-13), 36.3 (d, C-11), 38.8 (d, C-6), 49.8 (d, C-2), 71.6 (d, C-1), 110.8 (t, C-8), 130.0 (s, C-3), 146.1 (s, C-7). HR-MS: 226.1384 (*M^+^*, C_13_H_22_O_1_^32^S_1_^+^; calc. 226.1386).

**(**1*R*,2*R*,6*S*)-2-(Isobutylthio)-3-methyl-6-(prop-1-en-2-yl)cyclohex-3-enol (**33**)

Yield: 31%, ${\left[\alpha \right]}_{D}^{24}$+0.98(*c* = 0.41 in CHCl_3_), ^1^H-NMR (600 MHz, CDCl_3_): δ = 0.98 (d, 3H, J = 0.66 Hz, H-13), 0.99 (d, 3H, J = 6.7 Hz, H-14), 1.80 (1H, hept, J_12,13_ = J_12,14_ = 6.92 Hz, H-12), 1.82 (s, 3H, H-9), 1.83–1.84 (m, 3H, H-10), 1.97 (dm, 1H, J_5e,5a_ = 17.9 Hz, H-5e), 2.14–2.22 (m, 1H, CH-5a), 2.40 (dd, 1H, J_11,11′_ = 12.7 Hz, J_11,12_ = 7.5 Hz, H-11), 2.54 (dd, 1H, J_11′,11_ = 12.7 Hz, J_11′,12_ = 6.4 Hz, H-11′), 2.67 (dd, 1H, J_6a,5a_ = 11.9 Hz, J_6a,5e_ = 5.5 Hz, H-6a), 3.05 (br.s, 1H, H-2e), 4.01 (t, 1H, J = 2 Hz, H-1e), 4.84 (s, 1H, one of H-8), 4.96 (m, 1H, one of H-8), 5.51–5.54 (m, 1H, H-4). ^13^C NMR (150 MHz, CDCl_3_): δ = 21.7 (q, C-14), 22.0 (q, C-13), 22.0 (q, C-10), 22.4 (q, C-9), 24.0 (t, C-5), 28.9 (d, C-12), 38.7 (d, C-6), 42.0 (t, C-11), 51.8 (d, C-2), 70.9 (d, C-1), 110.9 (t, C-8), 123.7 (d, C-4), 129.8 (s, C-3), 146.0 (s, C-7). HR-MS: 193.1138 (*M^+^*-C_3_H_7_, C_11_H_17_O_1_^32^S_1_^+^; calc. 197.32).

(1*R*,2*R*,6*S*)-2-(*Tert*-butylthio)-3-methyl-6-(prop-1-en-2-yl)cyclohex-3-enol (**34**)

Yield: 82%, ${\left[\alpha \right]}_{D}^{24}$-94.73(*c* = 2.01 in CHCl_3_), ^1^H NMR (600 MHz, CDCl_3_): δ = 1.35 (s, 9H, 3H-12, 3H-13, 3H-14), 1.81 (s, 3H, H-9), 1.82 (br.s, 3H, H-10), 1.95 (dm, 1H, J_5a,5e_ = 17.9 Hz, J_5e,6a_ = 5.5 Hz, H-5e), 2.15 (dm, 1H, J_3a,3e_ = 17.9 Hz, J_3a,4a_ = 12 Hz, H-5a), 2.55 (dd, 1H, J_6a,5a_ = 12 Hz, J_6a,5e_ = 5.5 Hz, H-6a), 3.04 (br.s, 1H, H-2e), 4.02 (s, 1H, CH-1e), 4.82 (s, 1H, one of H-8), 4.93 (br.s, 1H, one of H-8), 5.46–5.49 (m, 1H, H-4); ^13^C NMR (150 MHz, CDCl_3_): δ = 21.8 (q, C-10), 22.3 (q, C-9), 23.7 (t, C-5) 31.1 (q, C-12, C-13, C-14), 38.8 (d, C-6), 43.9 (s, C-11), 47.8 (d, C-2), 72.9 (d, C-1), 110.7 (t, C-8), 123.2 (d, C-4), 130.2 (s, C-3), 146.2 (s, C-7). HR-MS: 239.1470 (*M^+^*, C_14_H_24_O_1_^32^S_1_^+^; calc. 240.1542).

(1*R*,2*R*,6*S*)-2-(4,5-Dihydrothiazol-2-ylthio)-3-methyl-6-(prop-1-en-2-yl)cyclohex-3-enol (**35**)

Yield: 60%, ${\left[\alpha \right]}_{D}^{24}$-31.1 (*c* = 0.27 in CHCl_3_), ^1^H NMR (600 MHz, CDCl_3_): δ = 1.77 (s, 3H, H-9), 1.79 (s, 3H, H-10), 1.95–2.02 (dm, 1H, J_5e,5a_ = 17.7 Hz, H-5e), 2.21–2.30 (dm, 1H, J_5e,5a_ = 17.7 Hz, J_5a,6a_ = 12 Hz, H-5a), 2.44 (dd, 1H, J_6a,5a_ = 12 Hz, J_6a,5e_ = 5.2 Hz, H-6a), 2.86 (br.s, 1H, -OH), 3.36 (t, 2H, J_13,14_ = 8 Hz, H-14), 4.14–4.26 (m, 4H, 2H-13, H-1, H-2), 4.83 (s, 1H, one of H-8), 4.89 (s, 1H, one of H-8), 5.63–5.67 (m, 1H, H-4); ^13^C NMR (150 MHz, CDCl_3_): δ = 21.8 (q, C-10), 22.2 (q, C-9), 24.4 (t, C-5), 35.2 (t, C-14), 39.5 (d, C-6), 52.5 (d, C-2), 64.0 (t, C-13), 70.5 (d, C-1), 111.0 (t, C-8), 126.7 (d, C-4), 127.4 (s, C-3), 145.8 (s, C-7), 165.2 (s, C-11). HR-MS: 269.0900 (*M^+^*, C_13_H_19_O_1_N_1_^32^S_2_^+^; calc. 269.0903).

(1*R*,2*R*,6*S*)-3-Methyl-2-(5-methyl-1*H*-benzo[*d*]imidazol-2-ylthio)-6-(prop-1-yl)cyclohex-3-enol (**36**)

Yield: 26%, m.p. 168.7 °C with decomposition, ${\left[\alpha \right]}_{D}^{25.5}$+7.2 (*c* = 0.36 in EtOH), ^1^H NMR (400 MHz, d^6^-DMSO): δ = 1.7 (s, 3H, H-7), 1.81 (s, 3H, H-10), 1.88–1.98 (m, 1H, one of H-5), 2.20–2.3 (m, 1H, one of H-5), 2.37 (s, 3H, H-10′), 2.40–2.46 (m, 1H, H-6), 4.05 (br.s, 1H, H-2), 4.28 (s, 1H, H-1), 4.76 (s, 1H, one of H-9), 4.78 (s, 1H, one of H-9), 5.03 (d, 1H, J = 4.86 Hz, -OH), 5.62–5.65 (m, 1H, H-4), 6.96–7.03 (m, 1H, H-6′), 7.14–7.26 (m, 1H, H-4′), 7.31–7.43 (m, 1H, H-7′); ^13^C NMR (100MHz, d^6^-DMSO): *δ* = 21.2 (q, C-10′), 21.9 (q, C-10, C-7), 24.6 (t, C-5), 39.4 (d, C-6), 52.9 (d, C-1), 70.1 (d, C-2), 110.4 (d, C-7′), 110.8 (t, C-9), 117.1 (d, C-6′), 122.6 (d, C-4′), 126.6 (d, C-4), 128.1 (s, C-8), 133.5 (s, C-5′), 141.8 (s, C-8′), 146.1 (s, C-3), 148.6 (s, C-9′), 167.8 (s, C-2′); HR-MS: 314.1452 (*M^+^*, C_18_H_22_O_1_N_2_^32^S_1_^+^; calc. 314.1447).

(1*R*,2*R*,6*S*)-2-(1*H*-Benzo[*d*]imidazol-2-ylthio)-3-methyl-6-(prop-1-yl)cyclohex-3-enol (**37**)

Yield: 35%, m.p. 166 °C with decomposition, ${\left[\alpha \right]}_{D}^{25.5}$+18.1 (*c* = 0.41 in EtOH), ^1^H NMR (400 MHz, d^6^-DMSO): δ = 1.71 (br.s, 3H, H-10), 1.82 (br.s, 3H, H-7), 1.9–1.98 (m, 1H, one of H-5), 2.21–2.31 (m, 1H, one of H-5), 2.43 (dd, 1H, J_1_ = 11.56 Hz, J_2_ = 4.49 Hz, H-6), 4.05–4.09 (m, 1H, H-2), 4.33 (br.s, 1H, H-2), 4.76 (br.s, 1H, one of H-9), 4.79 (br.s, 1H, one of H-9), 5.04 (d, 1H, J = 5.07 Hz, -OH), 5.63–5.67 (m, 1H, H-4), 7.09–7.16 (m, 2H, H-5′, H-4′), 7.35–7.39 (m, 1H, H-8′), 7.51–7.55 (m, 1H, H-9′); ^13^C NMR (100 Hz, d^6^-DMSO): δ = 21.8 (q, C-10), 21.8 (q, C-7), 24.7 (t, C-5), 39.4 (d, C-6), 52.8 (d, C-2), 70.1 (d, C-1), 110.8 (t, C-9), 121.2 (d, C-7′), 121.8 (d, C-4′), 122.3 (d, C-5′, C-6′), 126.6 (d, C-4), 127.9 (s, C-3), 135.4 (s, C-8′), 143.7 (s, C-9′), 149.4 (s, C-8), 168.1 (s, C-2′). HR-MS: 300.1292 (*M^+^*, C_17_H_20_O_1_N_2_^32^S_1_^+^; calc. 300.1291).

**(**1*R*,2*R*,6*S*)-2-Mercapto-3-methyl-6-(prop-1-en-2-yl)cyclohex-3-enol (**38**)

Epoxide(1*S*,5*S*,6*R*)*-***4** (286.4 mg, 1.91 mmol) was dissolved in dry THF (1.5 mL); then, (Me_3_Si)_2_S (0.50 mL) was added under argon. After this, a solution of n-Bu_4_NF × 3H_2_O (777.0 mg) in THF (4.5 mL) was added for 5 min. The reaction mixture was stirred for 15 min; then, a solution of citric acid (50%, 1.5 mL) was added. The reaction mixture was extracted with Et_2_O (15 mL), and the organic layer was washed with 20% citric acid solution (2 × 5 mL) and dried over Na_2_SO_4_. Then, the desiccant was filtered off, and the solvent was evaporated in vacuo. The residue was separated by column chromatography on silica gel (20 g), using 0–100% EtOAc gradient in hexane as an eluent, to give (1*R*,2*R*,6*S*)-2-mercapto-3-methyl-6-(prop-1-en-2-yl)cyclohex-3-enol (38, 63.3 mg, 0.34 mmol, 18%).

Yield: 18%, ${\left[\alpha \right]}_{D}^{28.8}$-35.6 (*c* = 0.57 in CHCl_3_), ^1^H NMR (500 MHz, CDCl_3_): δ = 1.65 (d, 1H, J_SH,2e_ = 8.7 Hz, 2-SH), 1.81–1.85 (m, 6H, H-9 and H-10), 2.00 (dm, 1H, ^2^J = 17.9 Hz, H-5e), 2.23 (ddm, 1H, ^2^J = 17.8 Hz, J_5a,6a_ = 12.0 Hz, H-5a), 2.64 (br. dd, J_6a,5a_ = 12.0 Hz, J_6a,5e_ = 5.3 Hz, H-6a), 3.29 (dm, 1H, J_2e,SH_ = 8.7 Hz, H-2e), 4.02 (dd, 1H, J_1e,2e_ = 2.4 Hz, J_1e,6a_ 1.8 Hz, H-1e), 4.83–4.86 (m, 1H, all J < 1.7 Hz, H-8), 4.96–4.99 (m, 1H, all J < 1.7 Hz, H-8′), 5.45–5.49 (m, 1H, H-4); ^13^C NMR (125 MHz, CDCl_3_): δ = 21.9 and 22.3 (2q, C-9 and C-10), 24.0 (t, C-5), 37.9 (d, C-6), 44.3 (d, C-2), 73.3 (d, C-1), 111.3 (t, C-8), 122.8 (d, C-4), 131.9 (s, C-3), 145.6 (s, C-7). HR-MS: 184.0914 (*M^+^*, C_10_H_16_OS^+^; calc. 184.0916).

## 4. Conclusions

We have elaborated different methods for the synthesis of epoxide *(1S*,*5S*,*6R)-***4** conjugated with an allylic bond from diacetate with the trans-diaxial arrangement of acetate groups and sodium *tert*-butoxide, with a good yield in toluene (81%), dioxane (92%), or chlorohydrin, and NaH in dioxane with a moderate yield (63%). The configuration of acetyl groups was shown to play an important role in the formation of the target product. Based on the reaction between sodium *tert*-butoxide and monoacetates, the mechanism of the reaction was proposed. Subsequently, a nucleophilic substitution reaction between the resulting epoxide and different nucleophiles was carried out to synthesize Prottremine derivatives without changing the absolute configuration of Prottremine.

## Figures and Tables

**Table 1:** Optimization of the conditions for epoxide (1*S*,5*S*,6*R*)*-***4**.

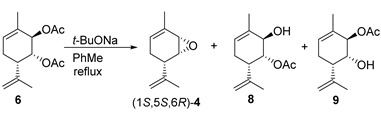				
Entry	Molar Ratio *t*-BuONa: Diacetate 6	Time, h	Yield of (*1S*,*5S*,*6R*)-4, %	Yield of 8,9, %
1	4:1	1.5	81	-
2	4:1	0.5	38	-
3	3:1	1	71	-
4	2:1	1	49	7

## Data Availability

Data are available from corresponding author upon reasonable request.

## Supplementary Materials

The following supporting information can be downloaded at: https://www.mdpi.com/article/10.3390/molecules28217303/s1, Figure S1–S63: The ^1^H and ^13^C NMR spectra of (*1S*,*5S*,*6R*)-**4**, (1*S*,2*S*,6*S*)-**6**, (1*S*,2*S*,6*S*)-**8**, (1*S*,2*S*,6*S*)-**9**, spectra of reaction of **6** and *t*-BuONa in dioxane rt 1 h and 24 h, in toluene rt 24h, **8**, **9**, **13**–**38**.
