# Supplementary material for: A New Stereoselective Approach to the Substitution of Allyl Hydroxy Group in para-Mentha-1,2-diol in the Search for New Antiparkinsonian Agents
Source: Molecules. 2023 Oct 27;28(21):7303. doi: 10.3390/molecules28217303 (PMC10650740; doi:10.3390/molecules28217303)
Supplement: Supplementary file 1 [file molecules-28-07303-s001.zip › molecules-2636538-supplementary.pdf]

Supplementary material

A New Stereoselective Approach to the Substitution of Allyl Hydroxy Group in *para*-Mentha-1,2-diol in the Search for New Antiparkinsonian Agents

Alexandra V. Podturkina, Oleg V. Ardashov, Konstantin P. Volcho * and Nariman F. Salakhutdinov

Department of Medicinal Chemistry, N. N. Vorozhtsov Novosibirsk Institute of Organic Chemistry,
Siberian Branch, Russian Academy of Sciences, Lavrentiev Ave. 9, 630090 Novosibirsk, Russia; podturkina@nioch.nsc.ru (A.V.P.); ardashov@nioch.nsc.ru (O.V.A.); anvar@nioch.nsc.ru (N.F.S.)
* Correspondence: volcho@nioch.nsc.ru

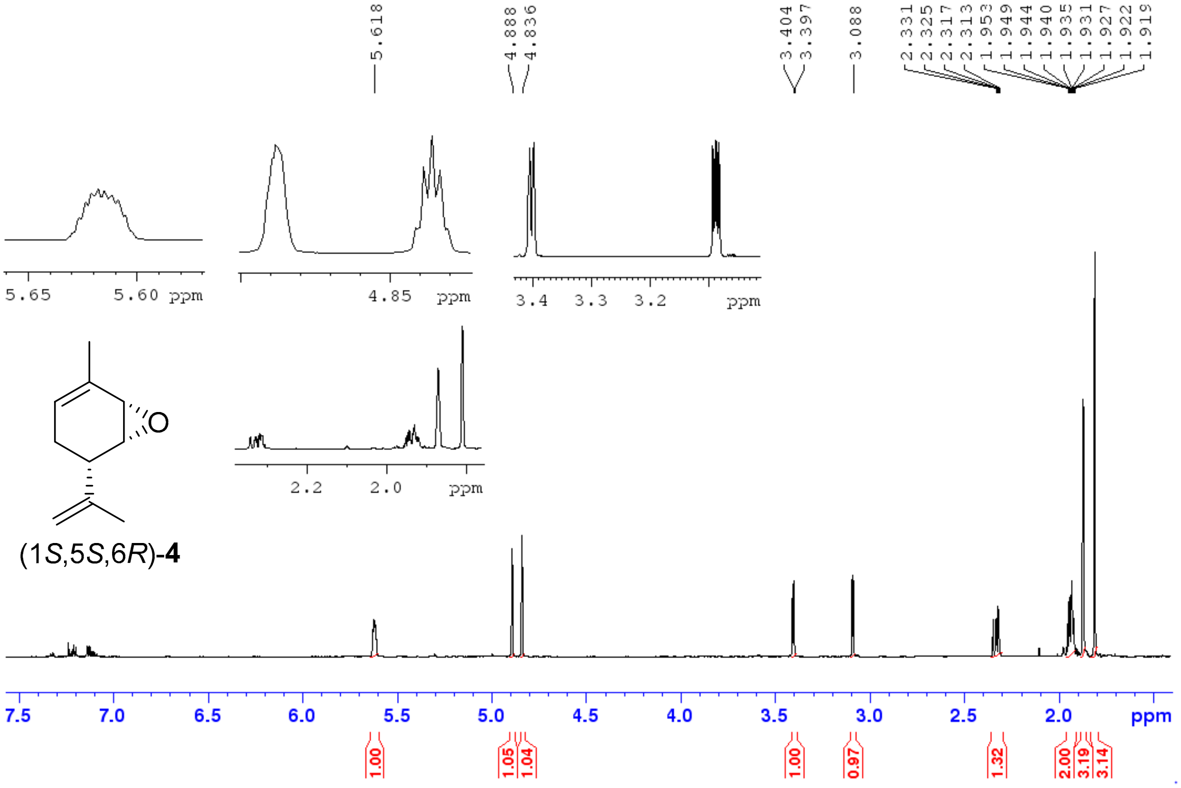

Figure S1: ^{1}H (600 MHz, CDCl$_3$) spectrum of (1*S*,5*S*,6*R*)-**4**

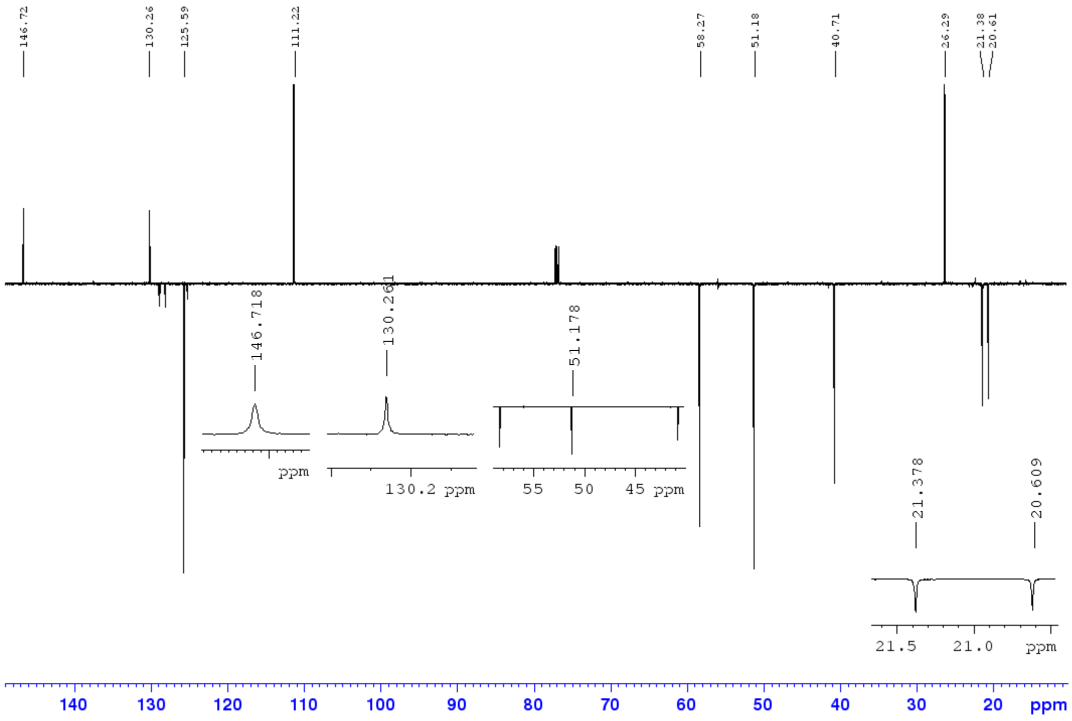

Figure S2: ^{13}C (150 MHz, $CDCl_3$) spectrum of (1*S*,5*S*,6*R*)-**4**

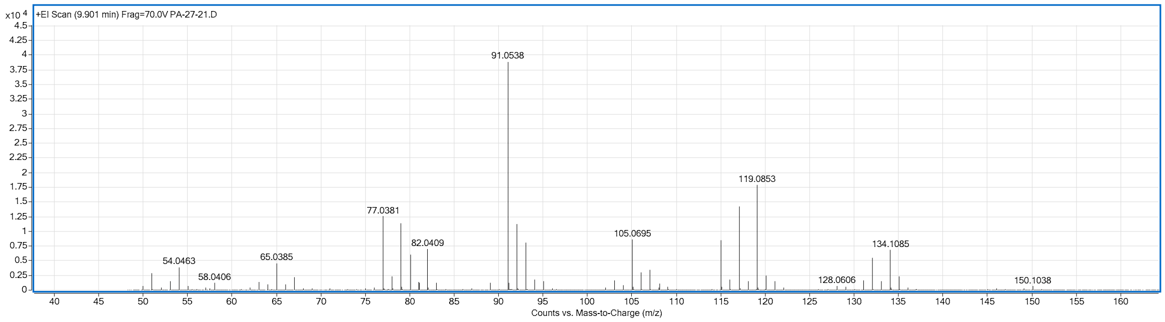

Figure S3: HR-MS spectrum of (1*S*,5*S*,6*R*)-**4**

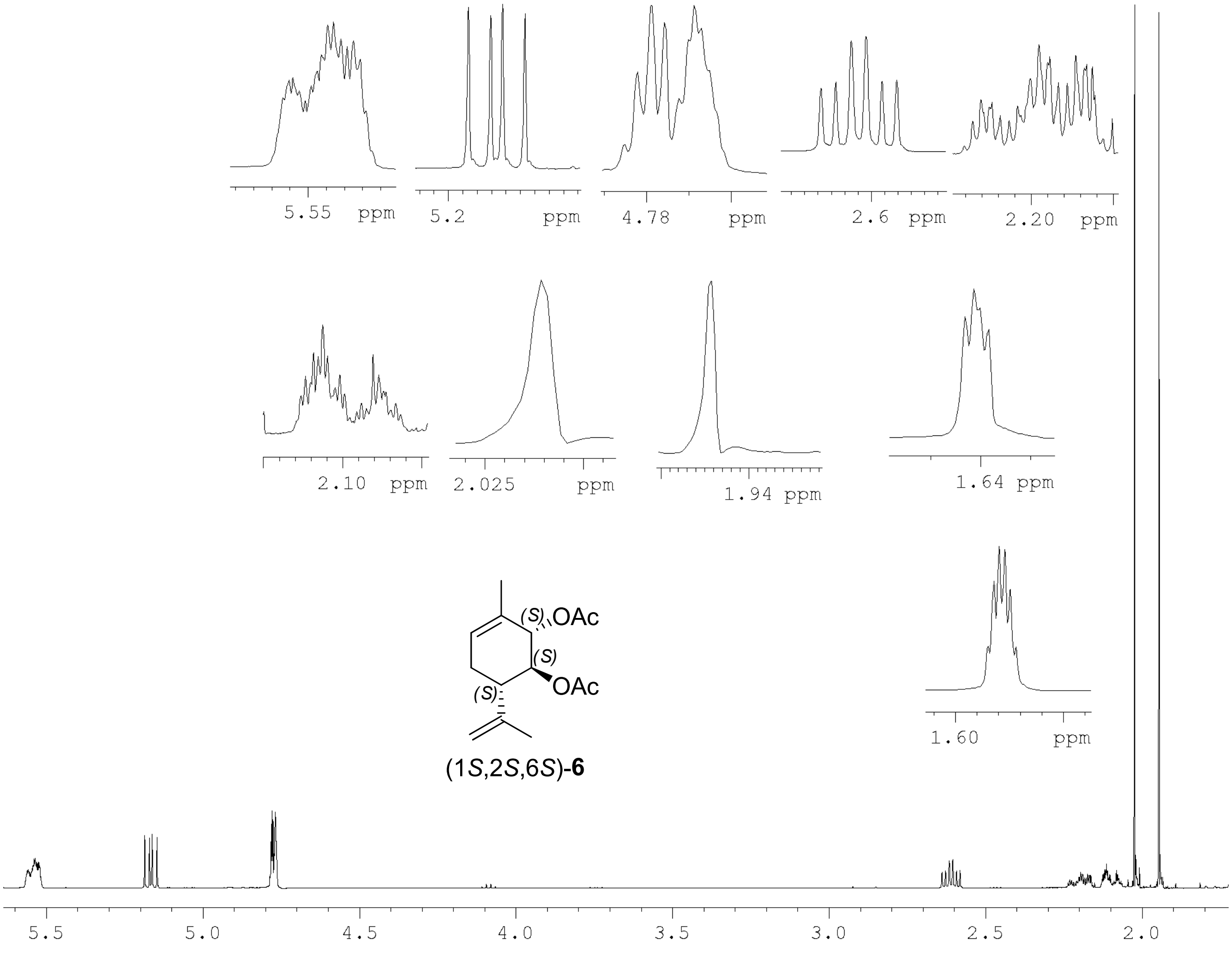

Figure S4: ^{1}H (500 MHz, $CDCl_3$) spectrum of (1*S*,2*S*,6*S*)-**6**.

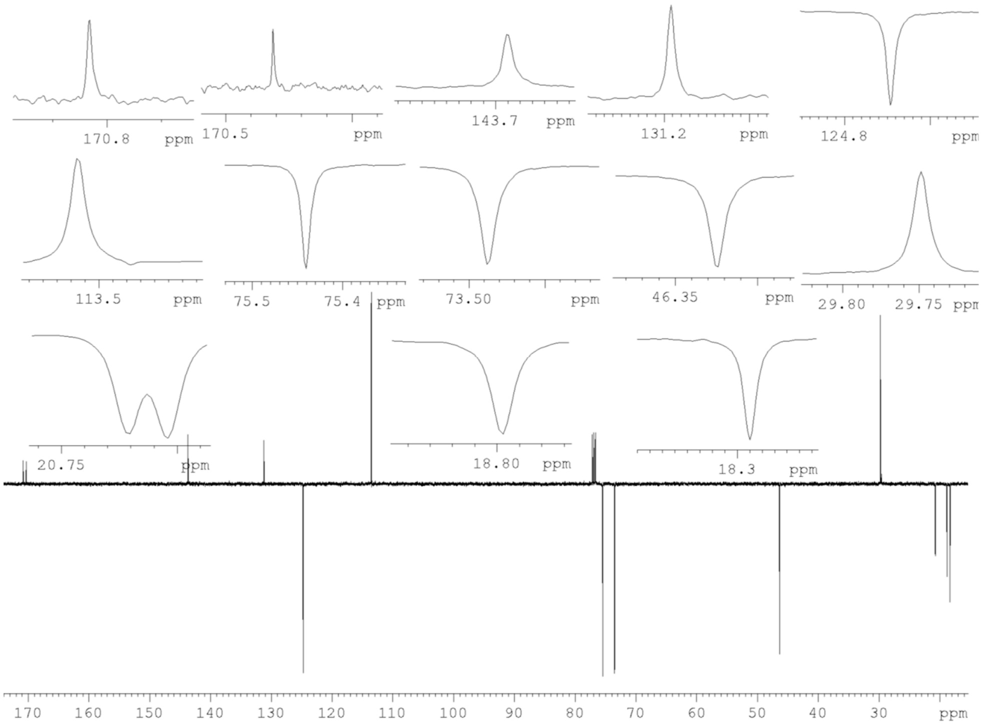

Figure S5: ^{13}C (125 MHz, $CDCl_3$) spectrum of (1*S*,2*S*,6*S*)-**6**.

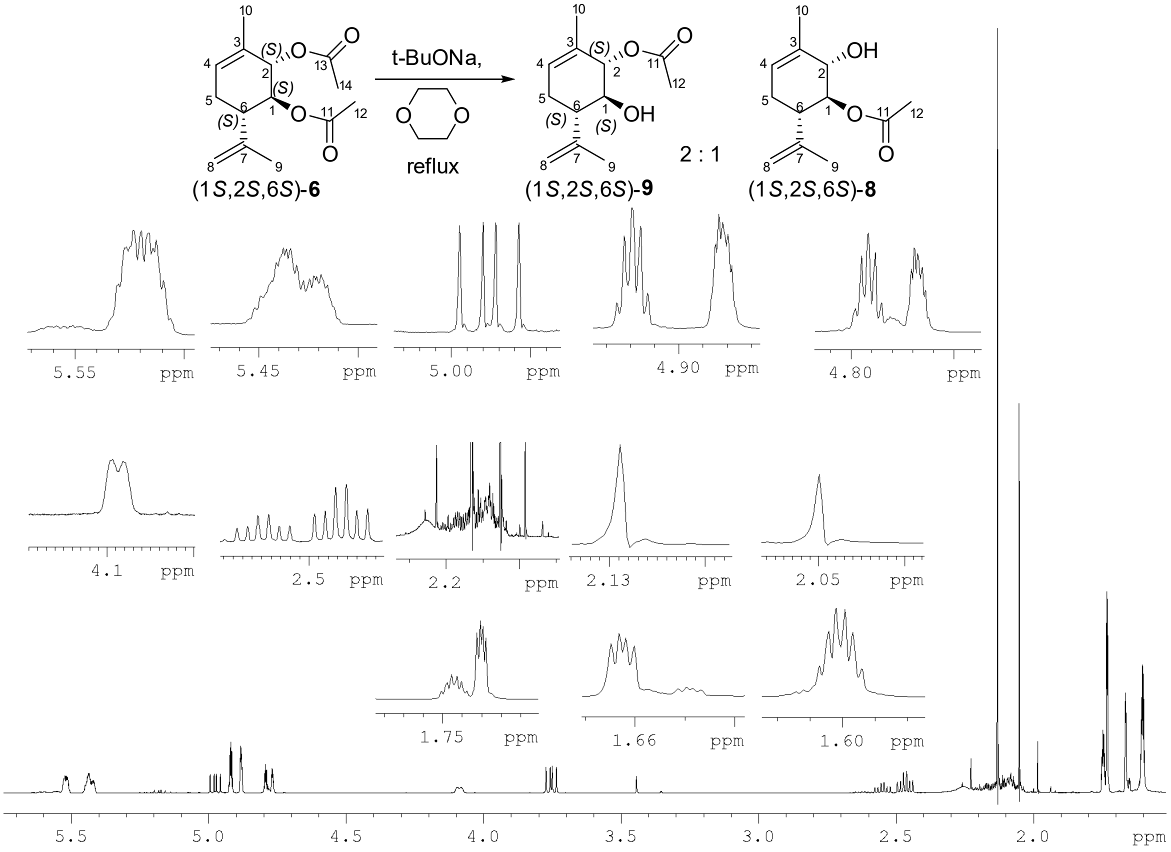

Figure S6: ^{1}H (500 MHz, $CDCl_3$) spectrum of (1*S*,2*S*,6*S*)-**8** and (1*S*,2*S*,6*S*)-**9**.

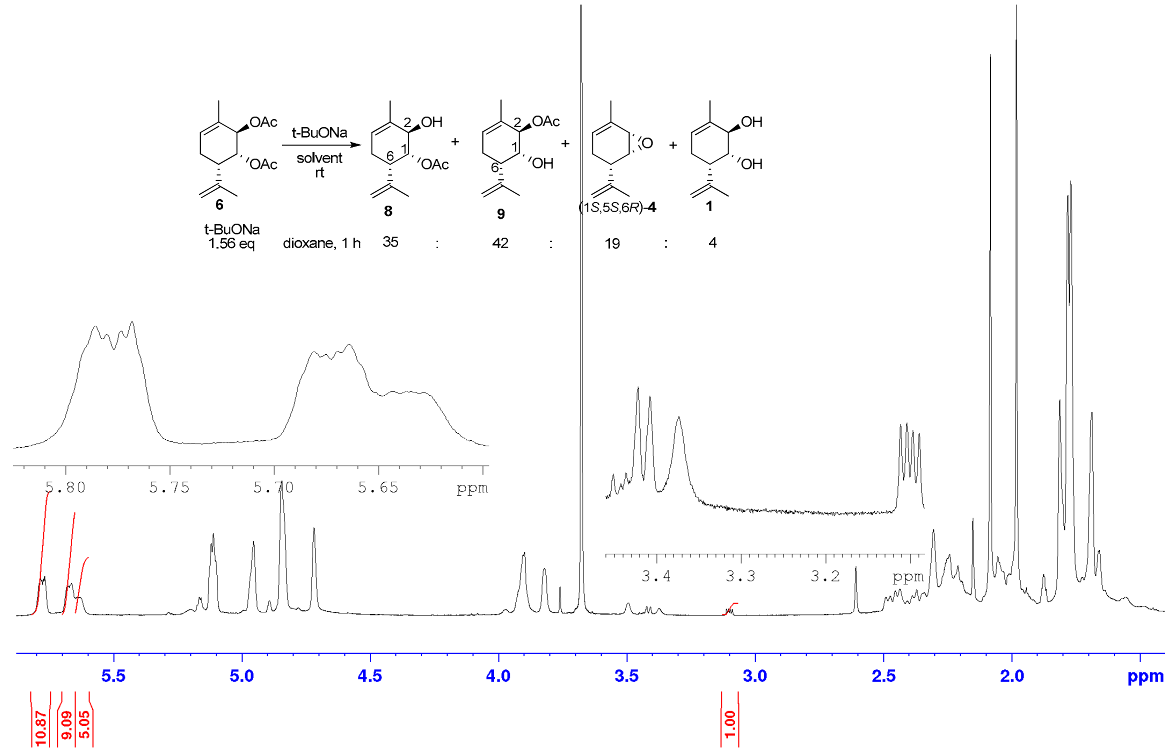

Figure S7: ^{1}H (400 MHz, $CDCl_3$) spectrum of reaction **6** and t-BuONa in dioxane, rt, 1h.

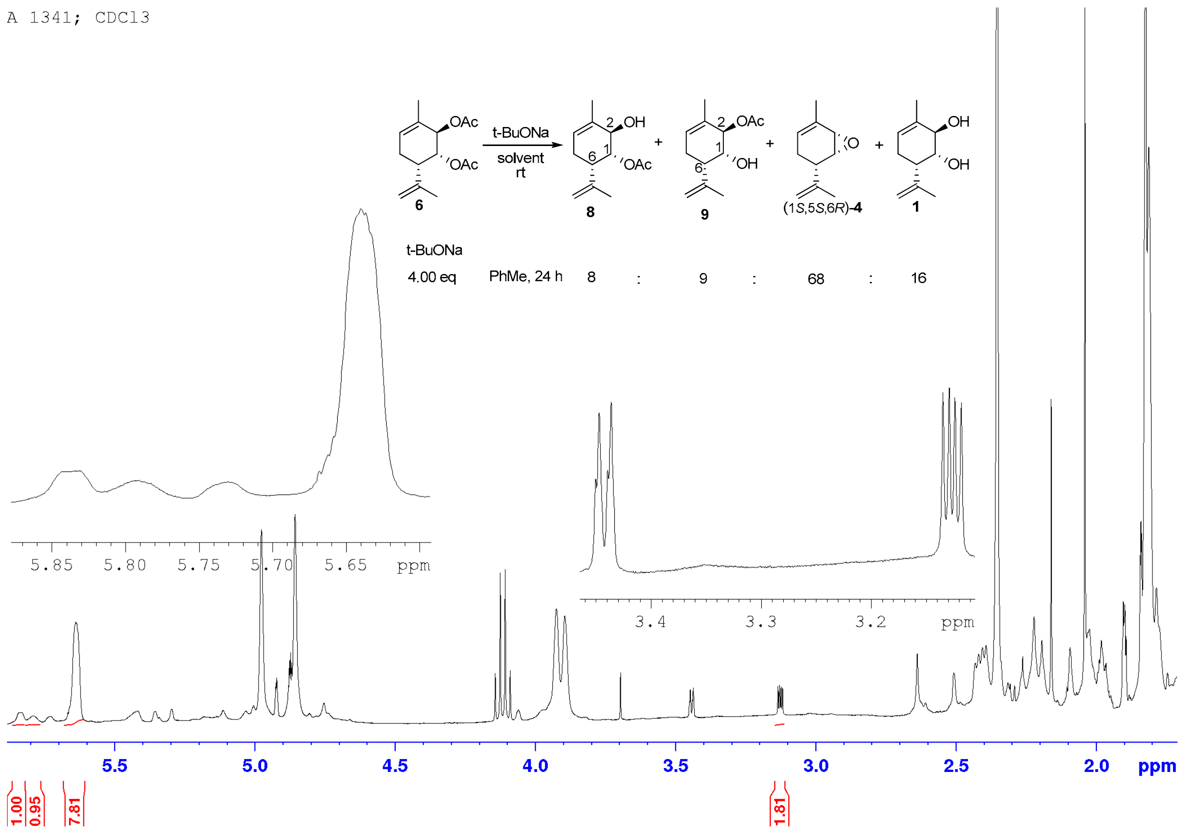

Figure S8: ^{1}H (400 MHz, $CDCl_3$) spectrum of reaction **6** and t-BuONa in toulene, rt, 24h.

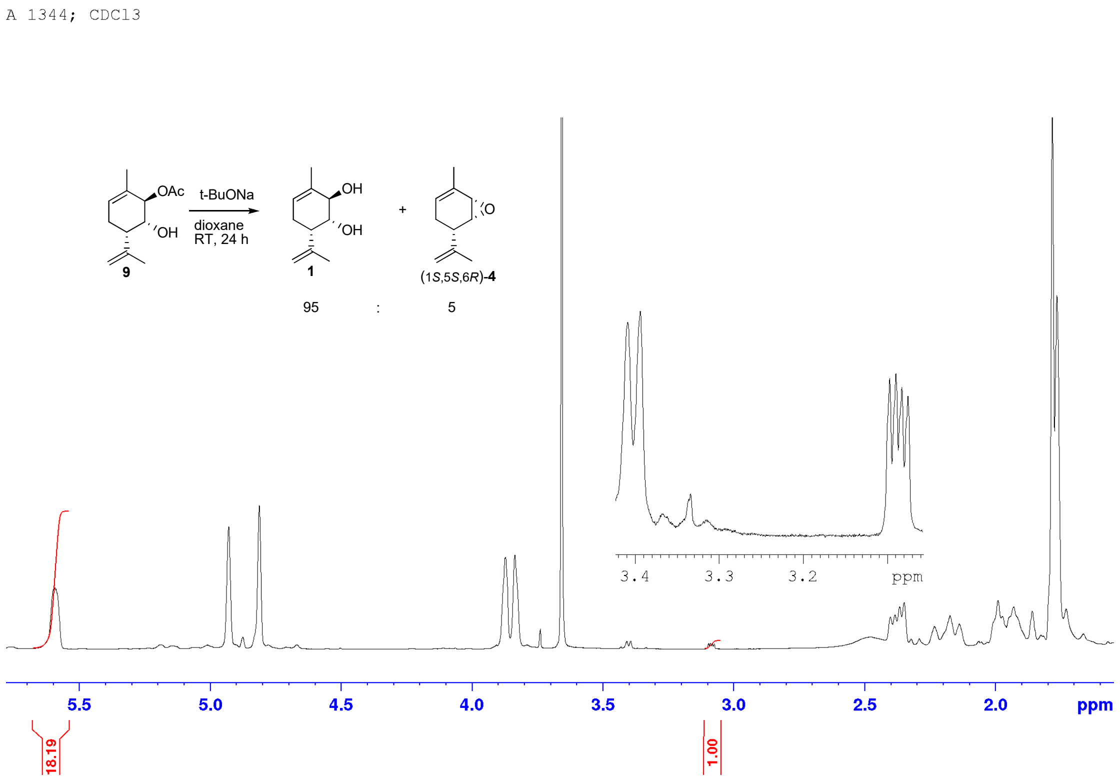

Figure S9: ^{1}H (400 MHz, $CDCl_3$) spectrum of reaction **6** and t-BuONa in dioxane, rt, 24h.

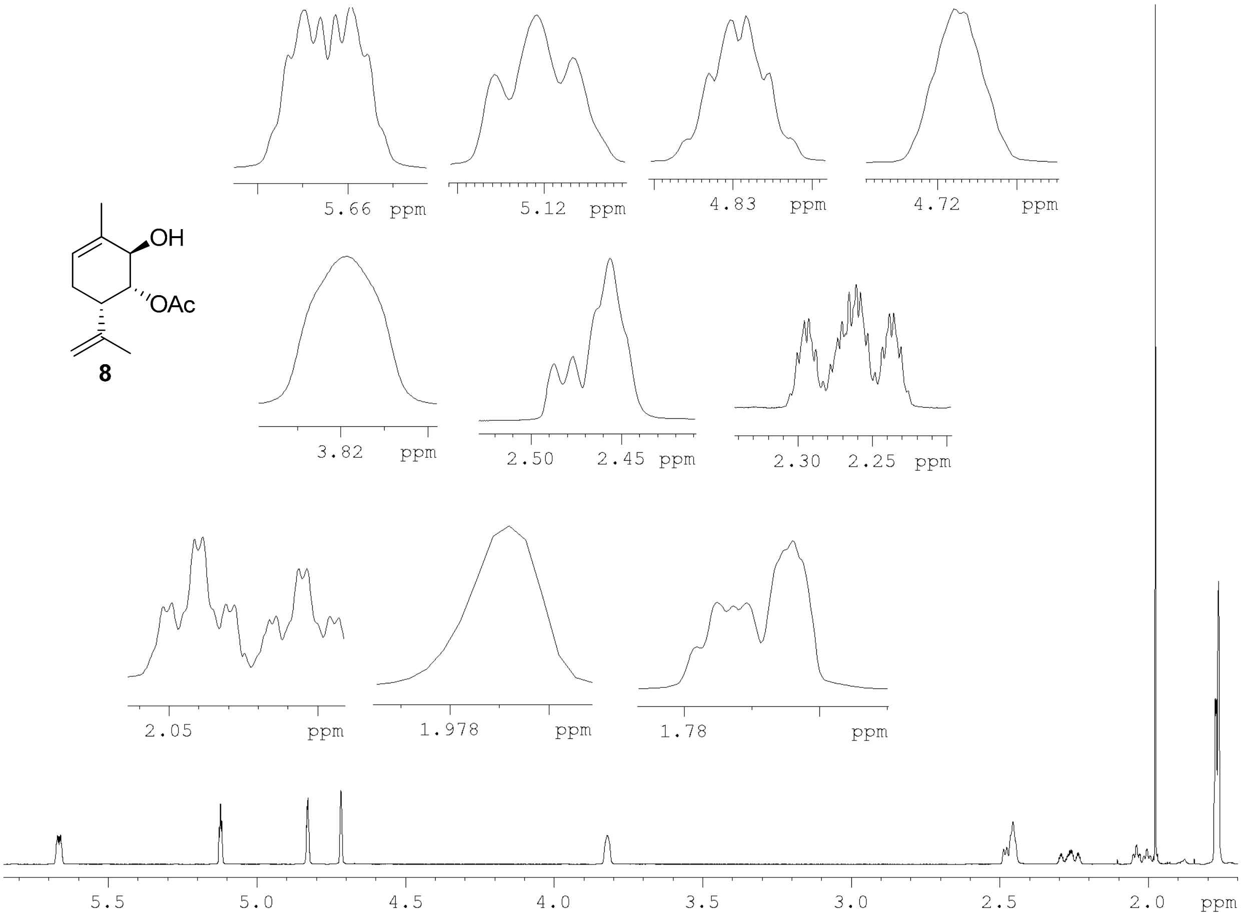

Figure S10: ^{1}H (400 MHz, $CDCl_3$) spectrum of **8**.

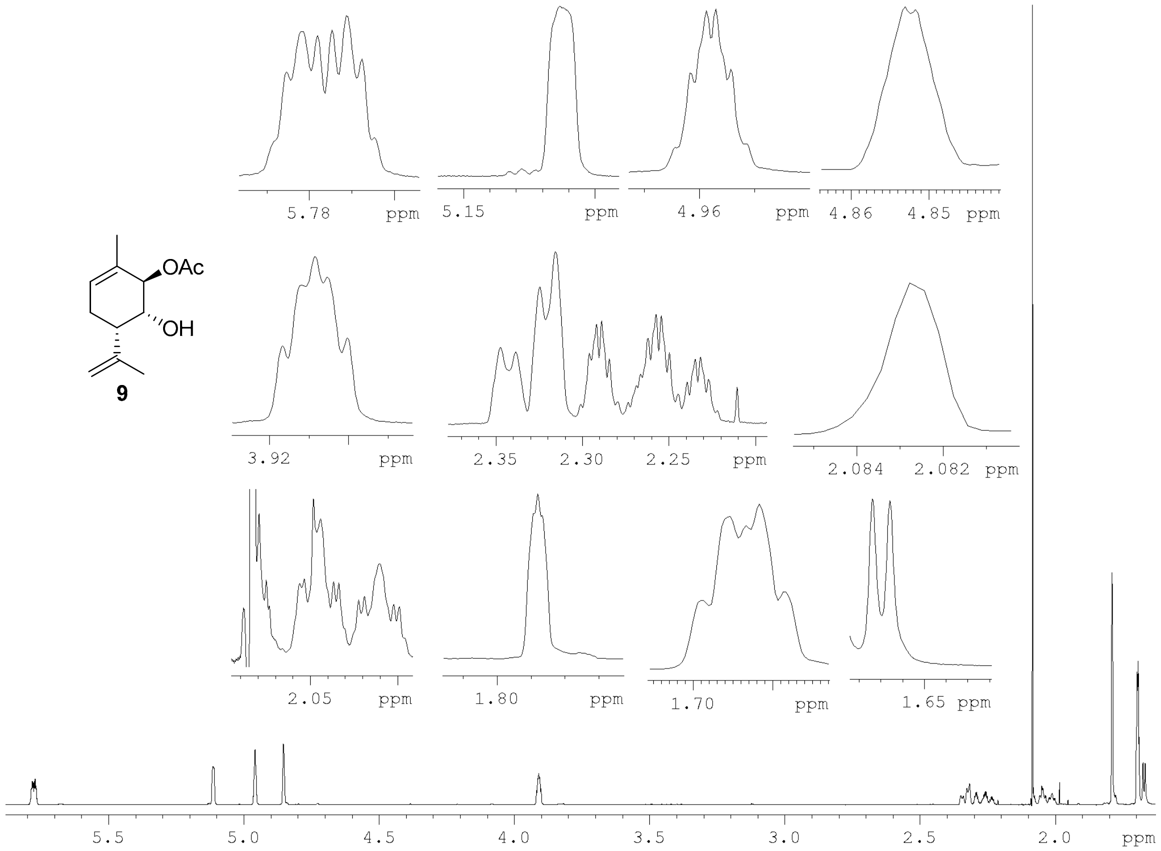

Figure S11: ^{1}H (400 MHz, $CDCl_3$) spectrum of **9**.

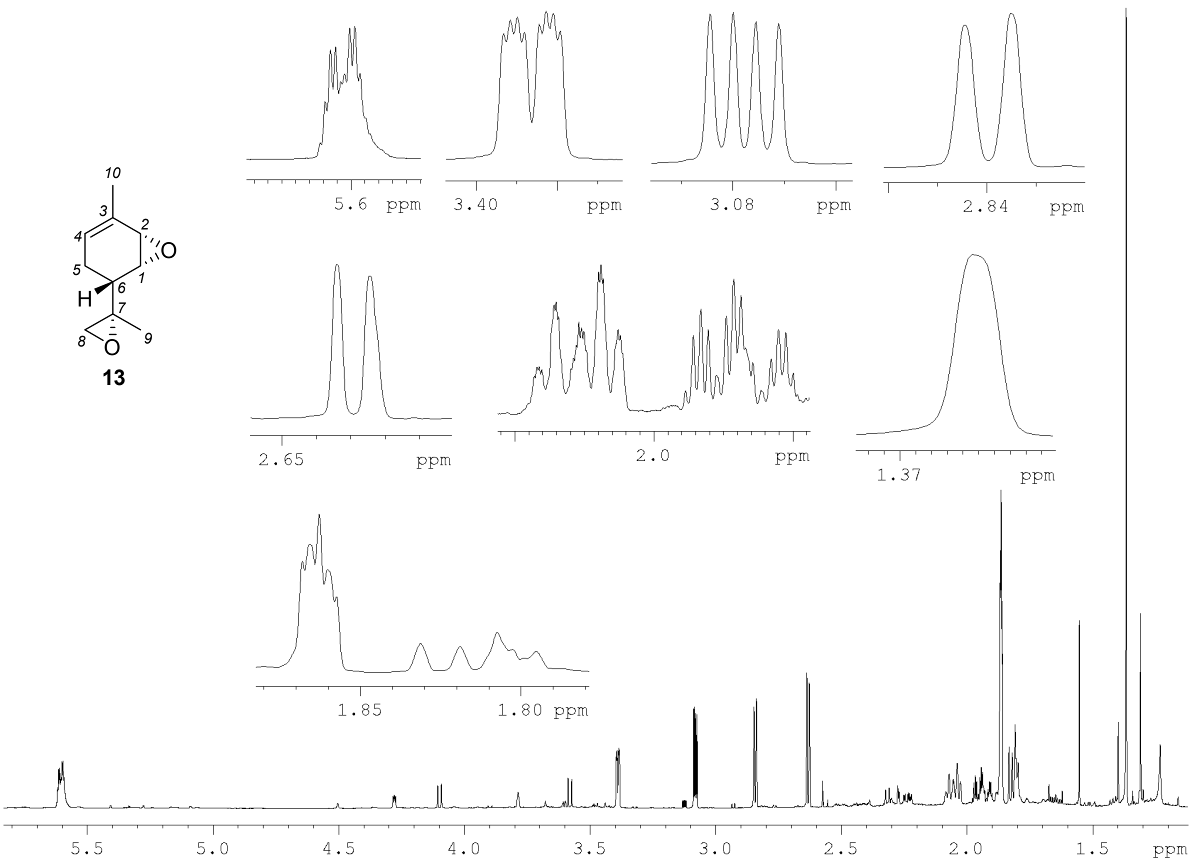

Figure S12: ^{1}H (500 MHz, $CDCl_3$) spectrum of **13**.

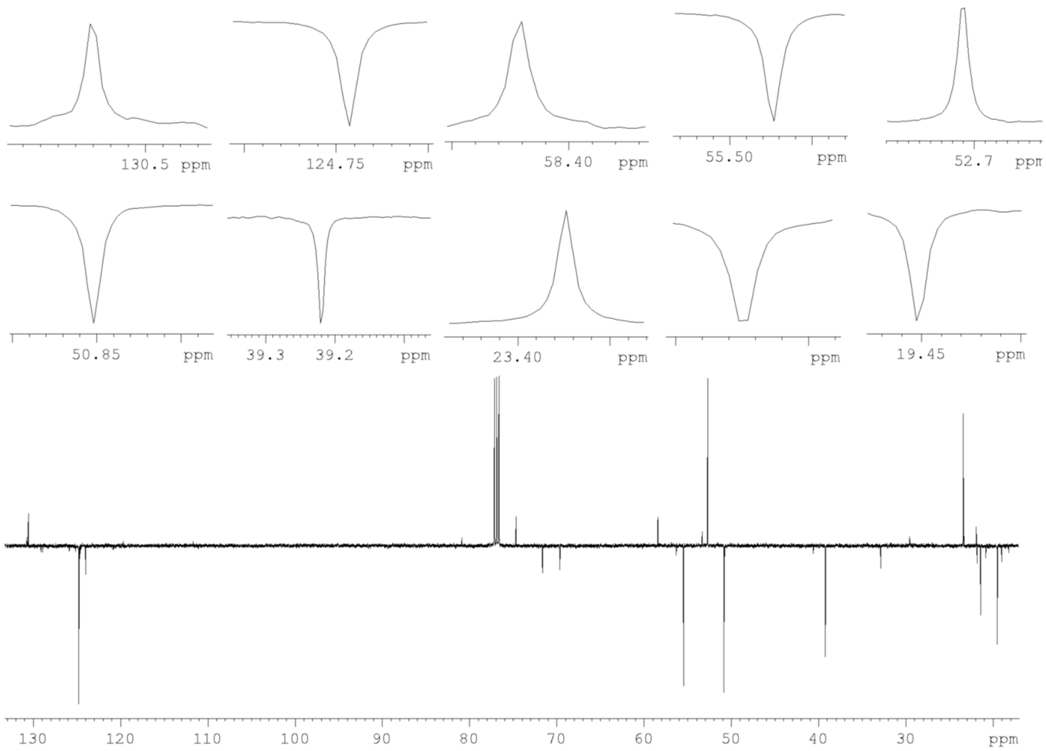

Figure S13: ^{13}C (125 MHz, $CDCl_3$) spectrum of **13**.

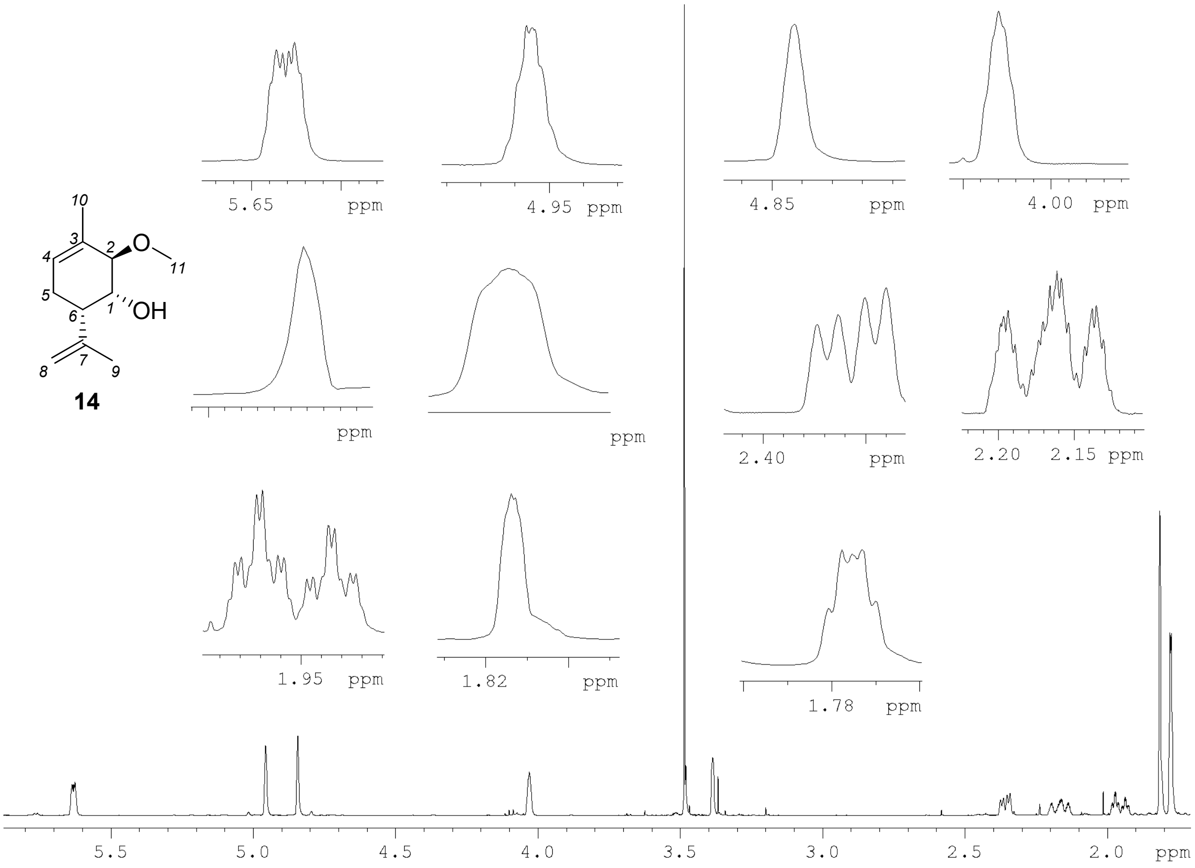

Figure S14: ^{1}H (500 MHz, $CDCl_3$) spectrum of **14**.

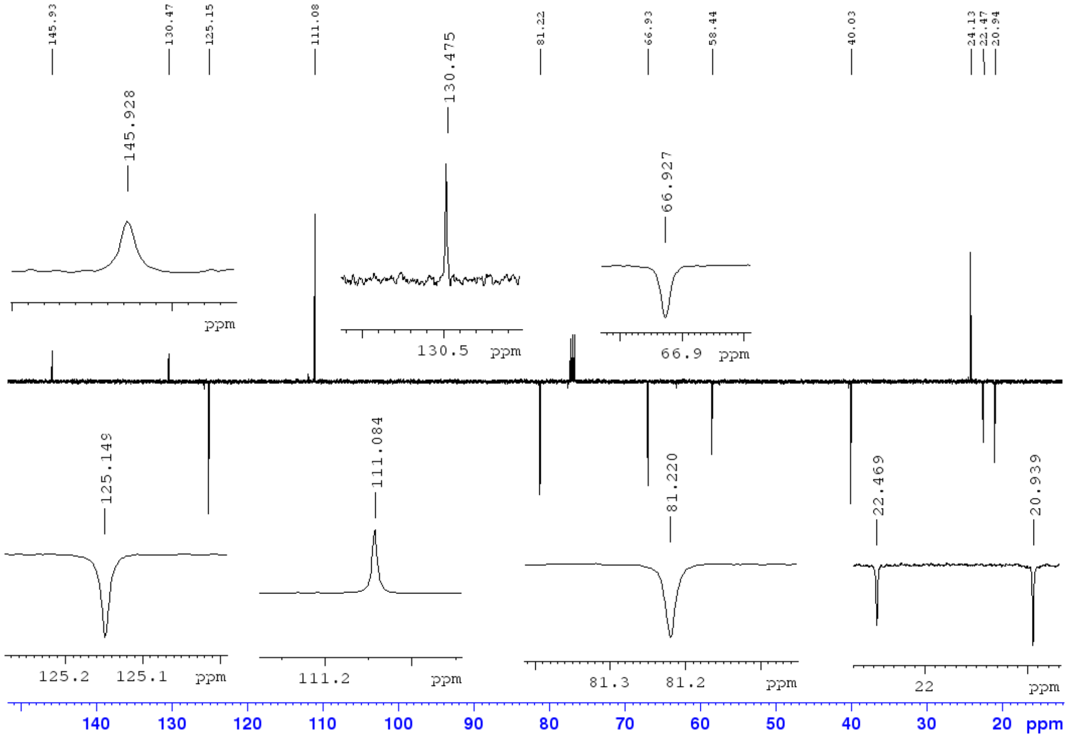

Figure S15: ^{13}C (125 MHz, $CDCl_3$) spectrum of **14**.

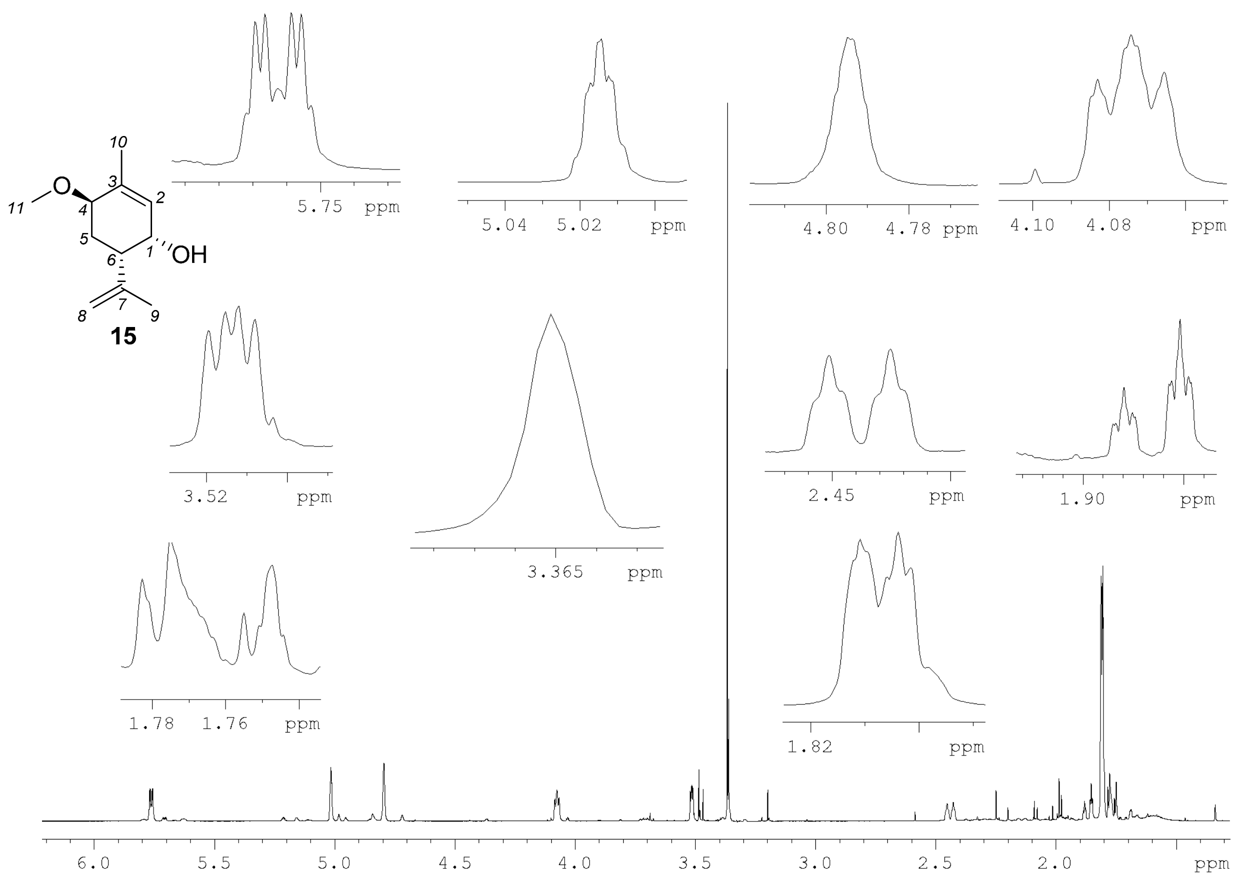

Figure S16: ^{1}H (500 MHz, $CDCl_3$) spectrum of **15**.

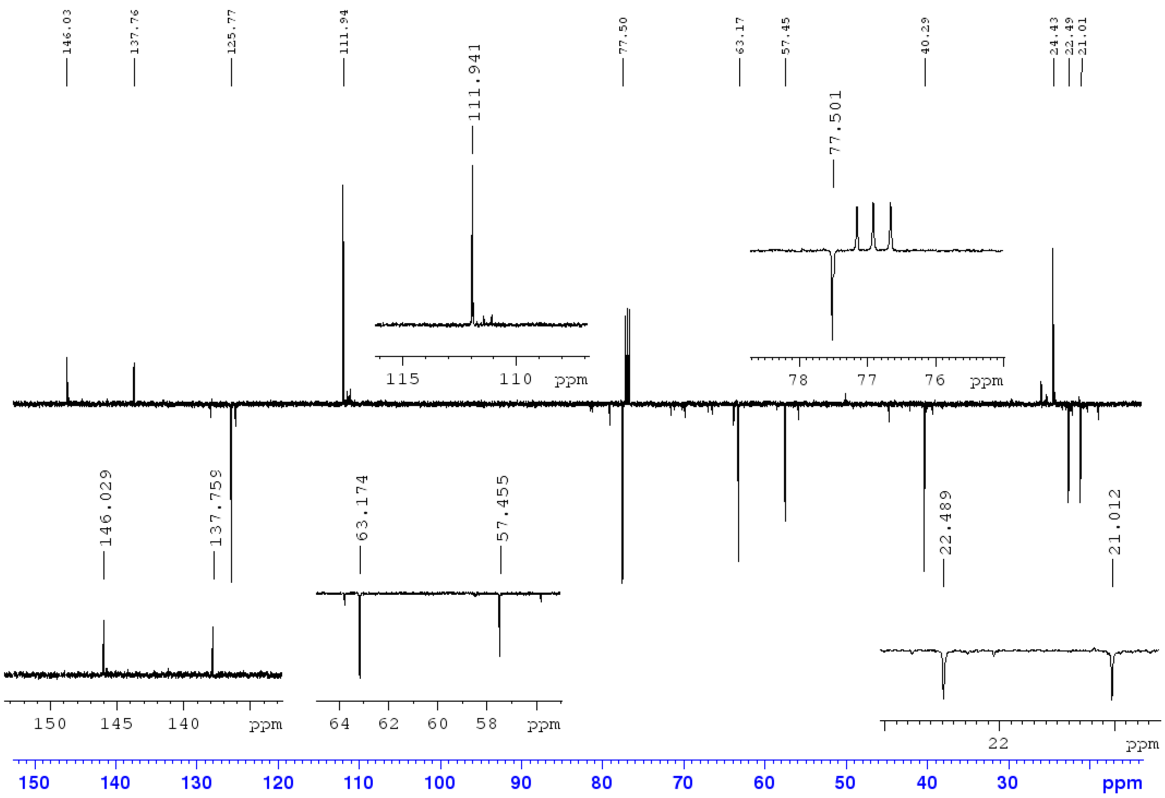

Figure S17: ^{13}C (125 MHz, $CDCl_3$) spectrum of **15**.

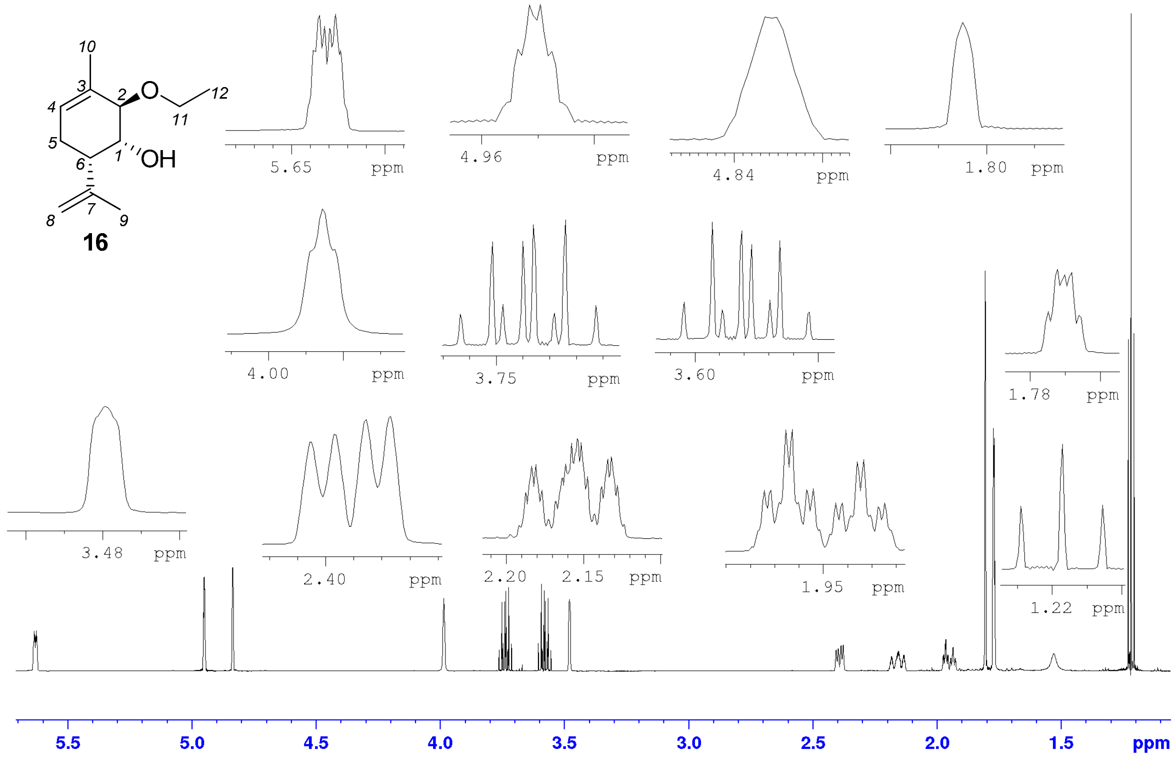

Figure S18: ^{1}H (600 MHz, $CDCl_3$) spectrum of **16**.

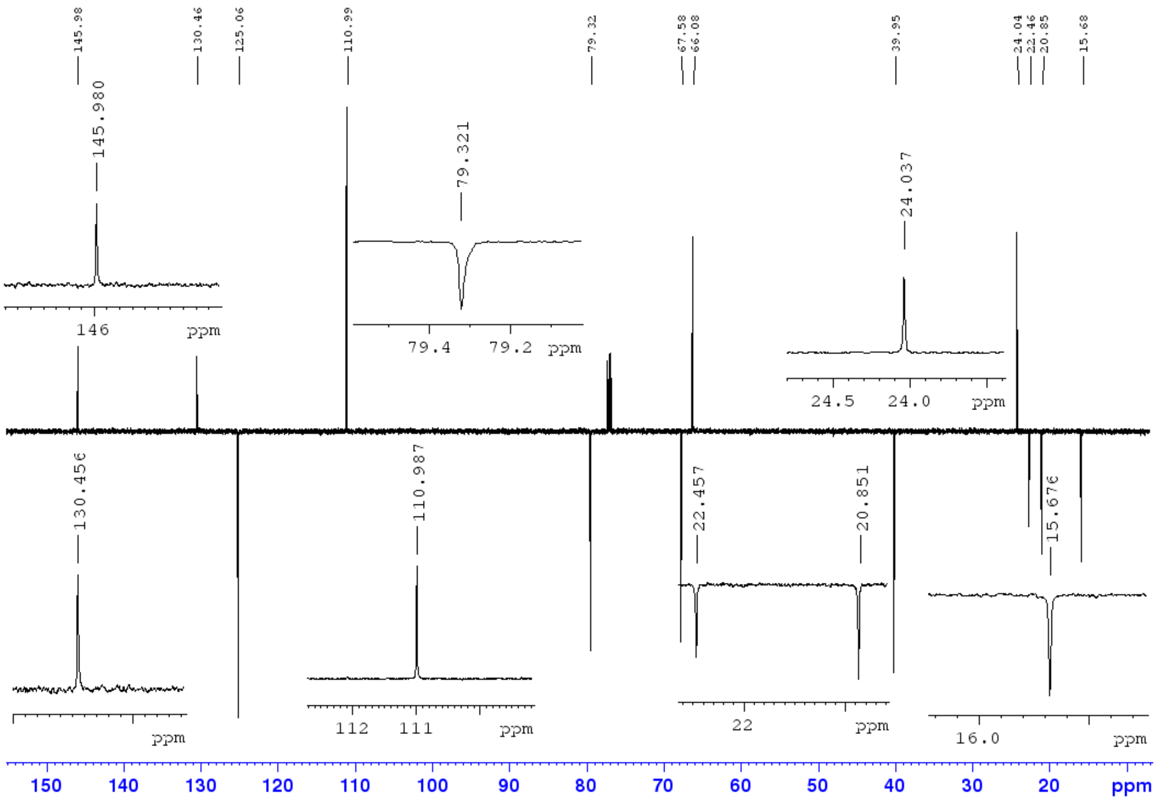

Figure S19: ^{13}C (150 MHz, $CDCl_3$) spectrum of **16**.

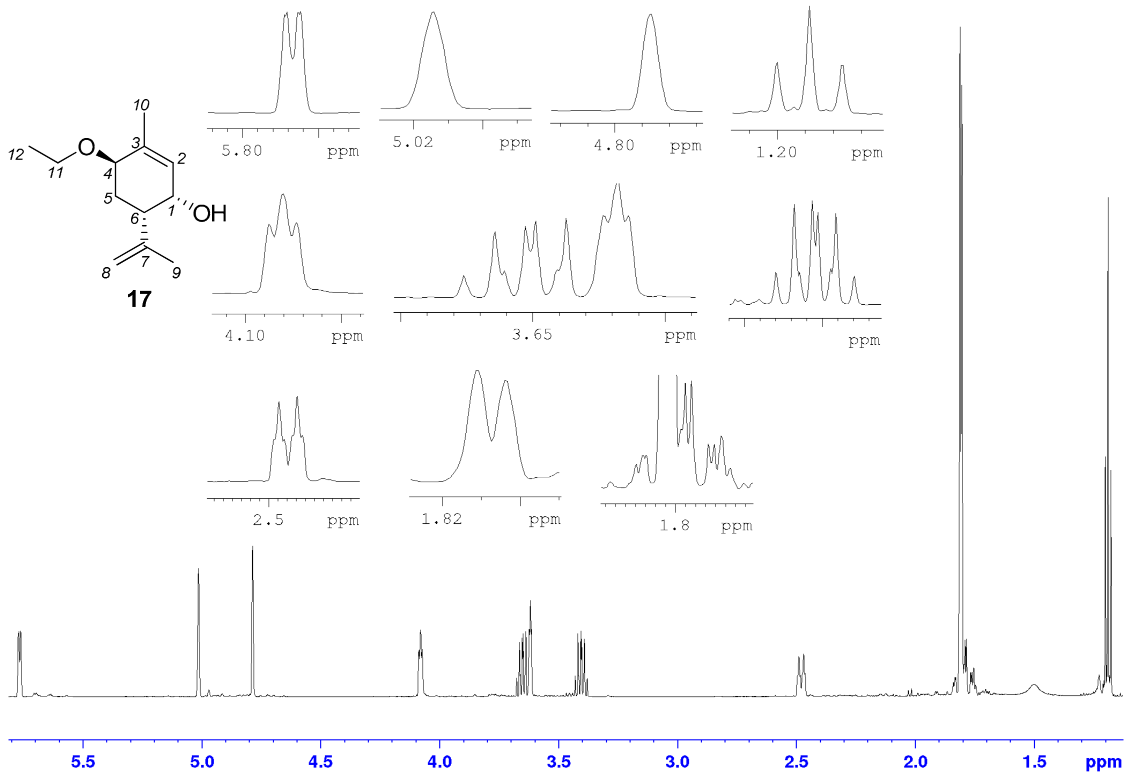

Figure S20: ^{1}H (600 MHz, $CDCl_3$) spectrum of **17**.

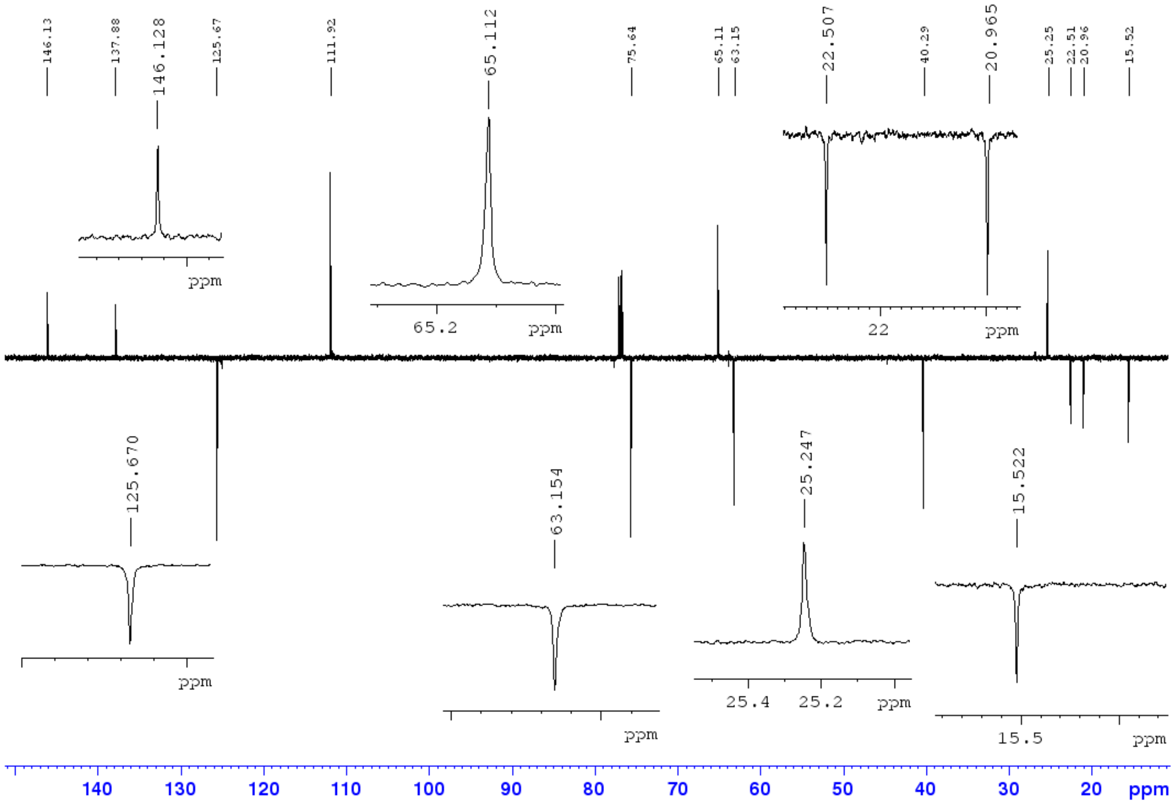

Figure S21: ^{13}C (150 MHz, $CDCl_3$) spectrum of **17**.

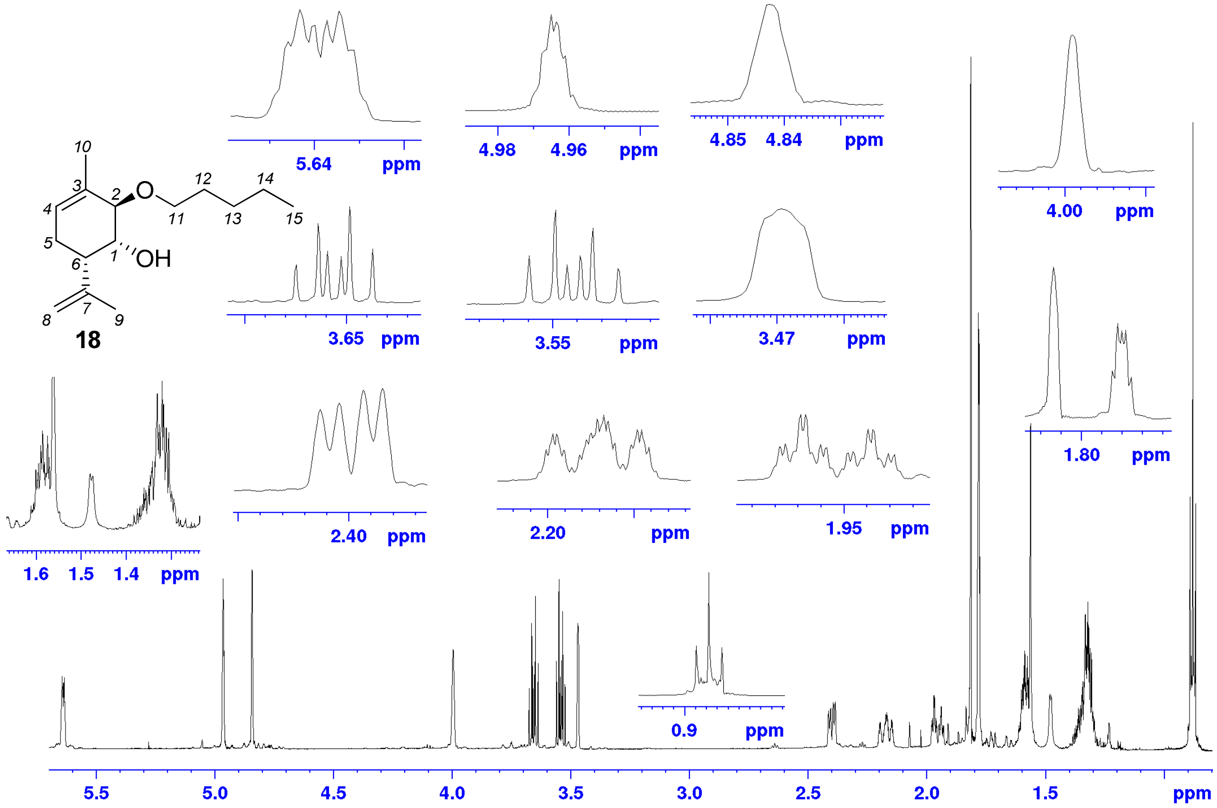

Figure S22: ^{1}H (600 MHz, $CDCl_3$) spectrum of **18**.

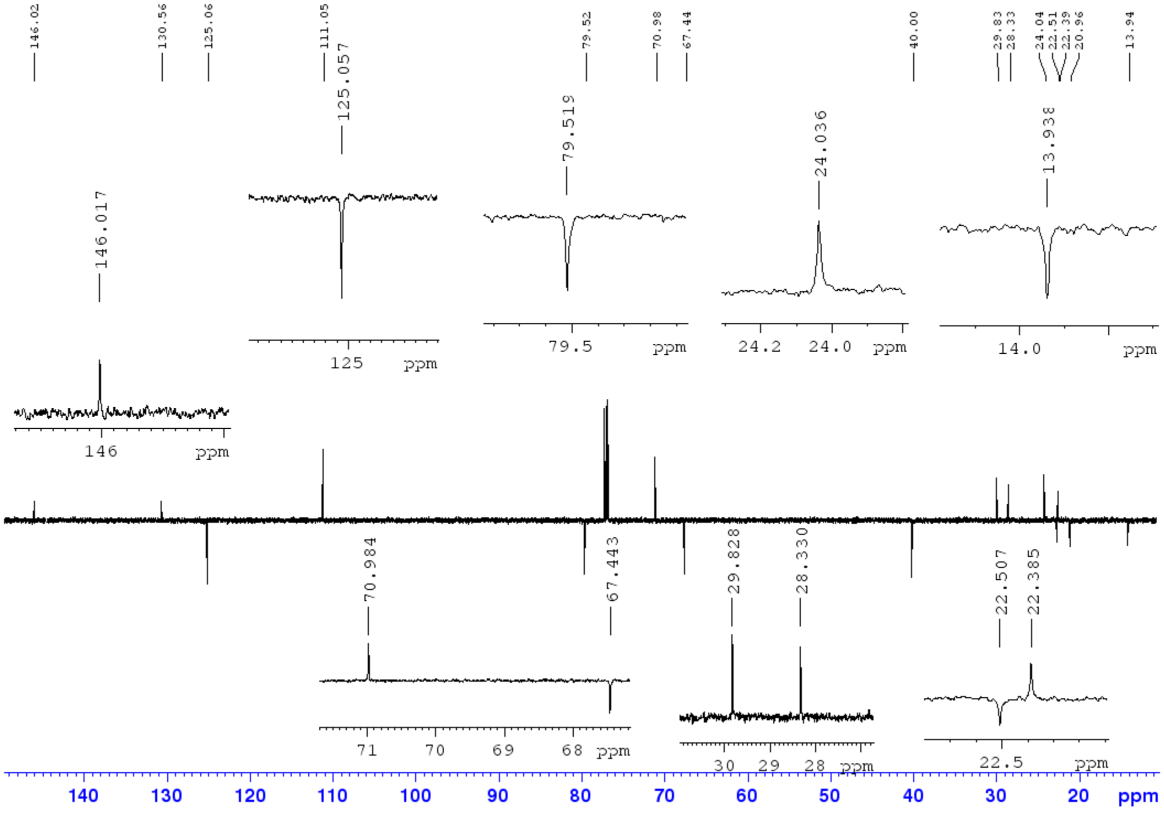

Figure S23: ^{13}C (150 MHz, $CDCl_3$) spectrum of **18**.

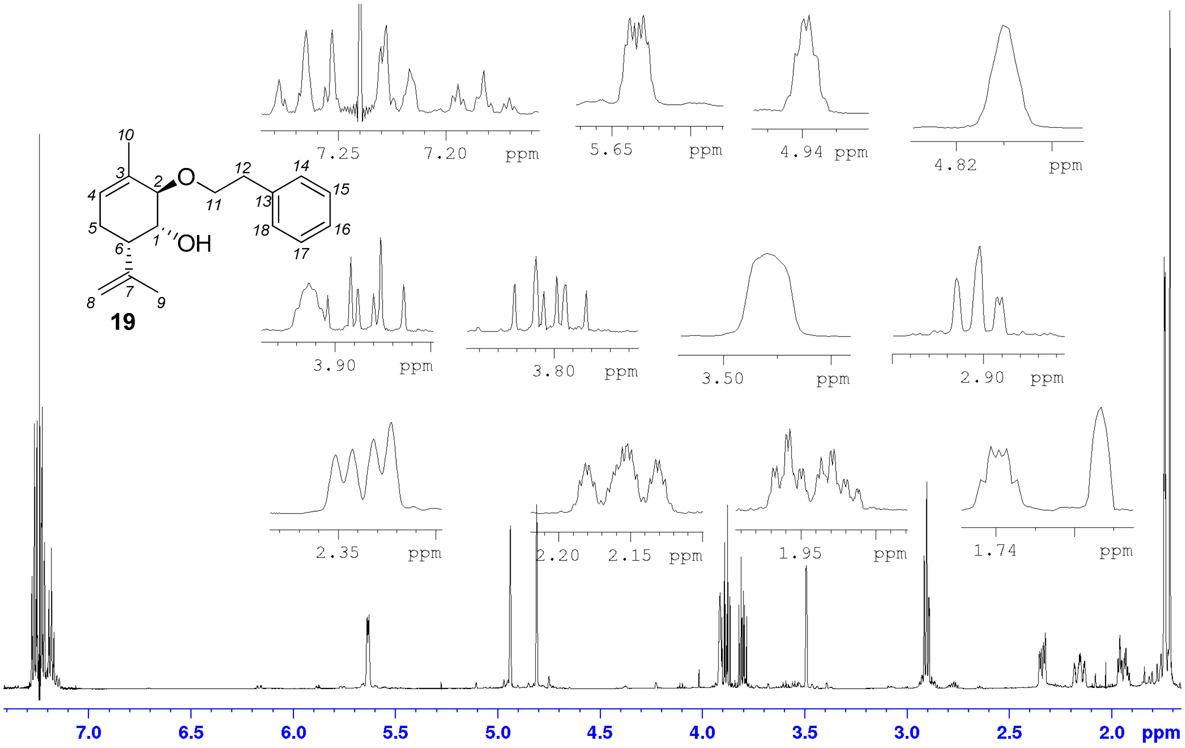

Figure S24: ^{1}H (600 MHz, $CDCl_3$) spectrum of **19**.

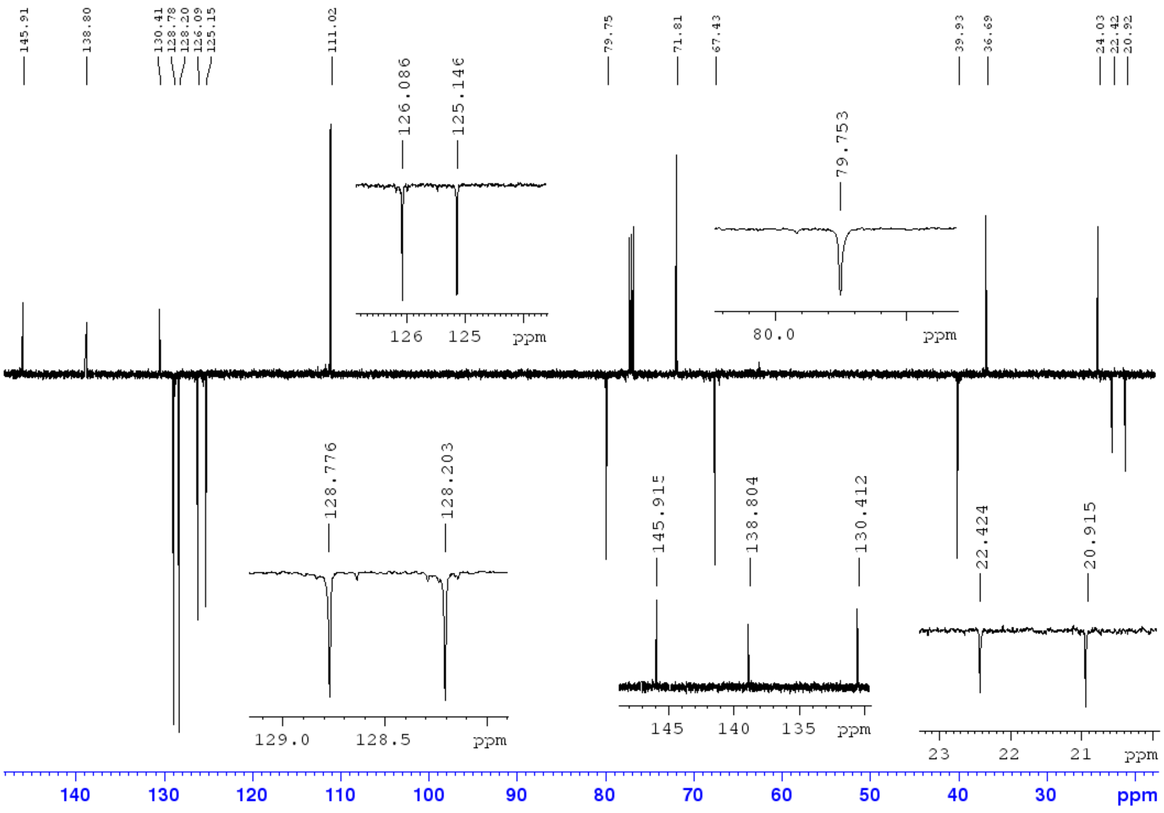

Figure S25: ^{13}C (150 MHz, $CDCl_3$) spectrum of **19**.

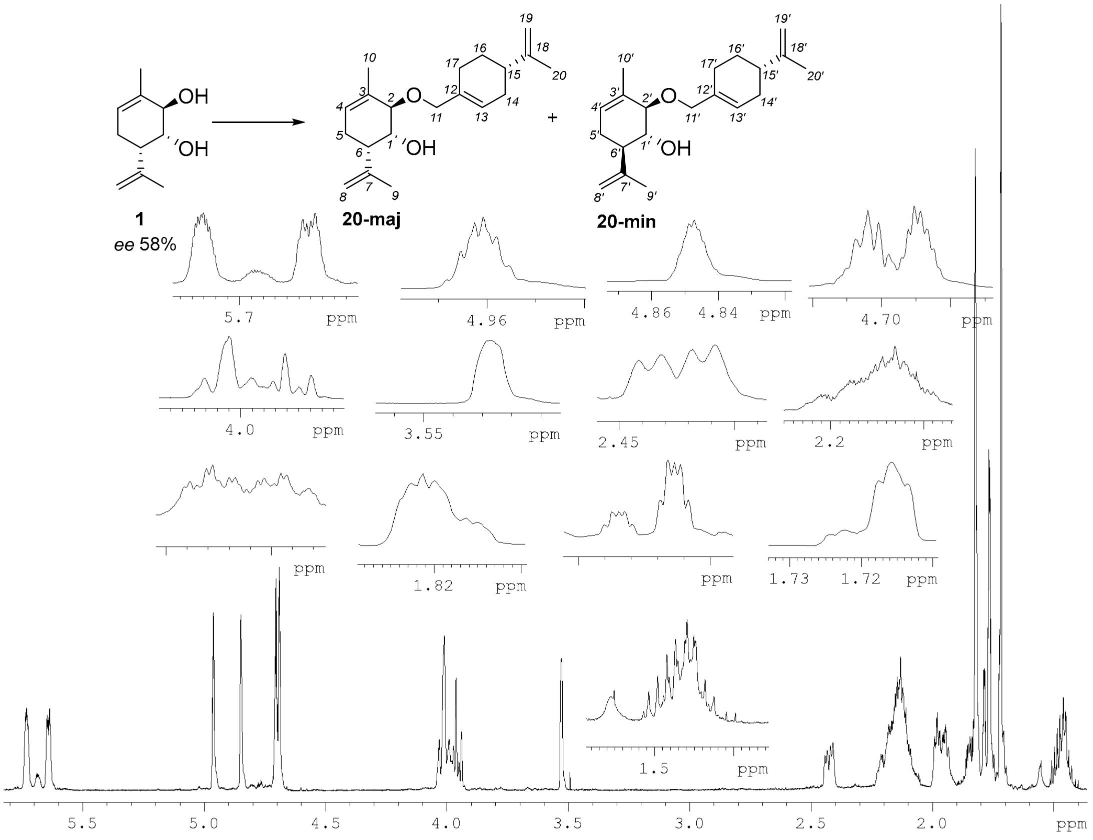

Figure S26: ^{1}H (500 MHz, $CDCl_3$) spectrum of **20**.

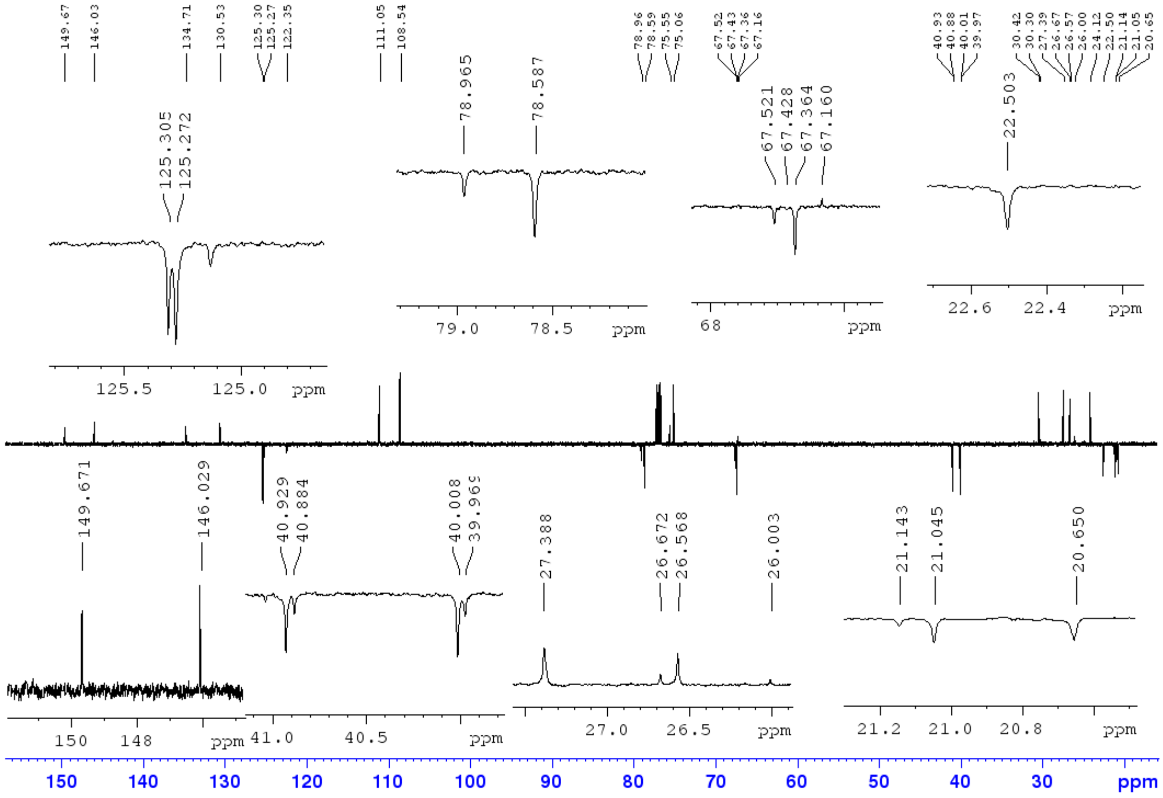

Figure S27: ^{13}C (125 MHz, $CDCl_3$) spectrum of **20**.

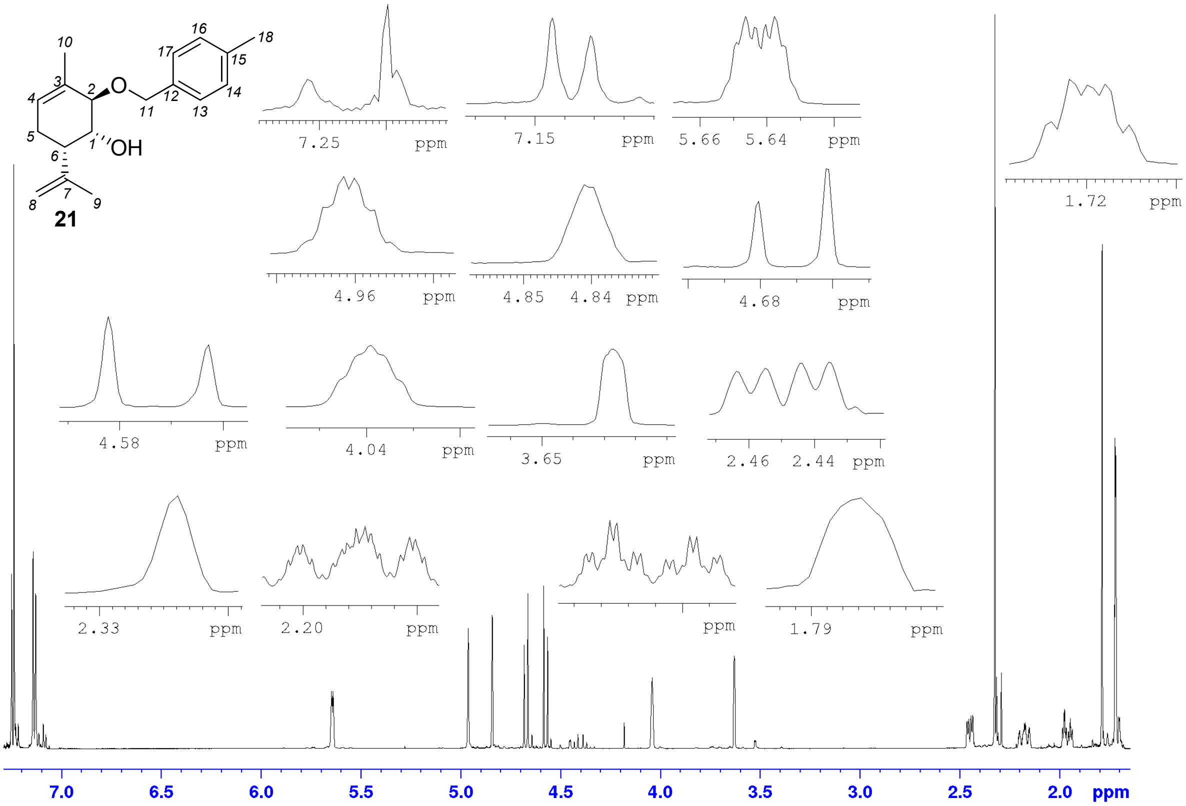

Figure S28: ^{1}H (600 MHz, $CDCl_3$) spectrum of **21**.

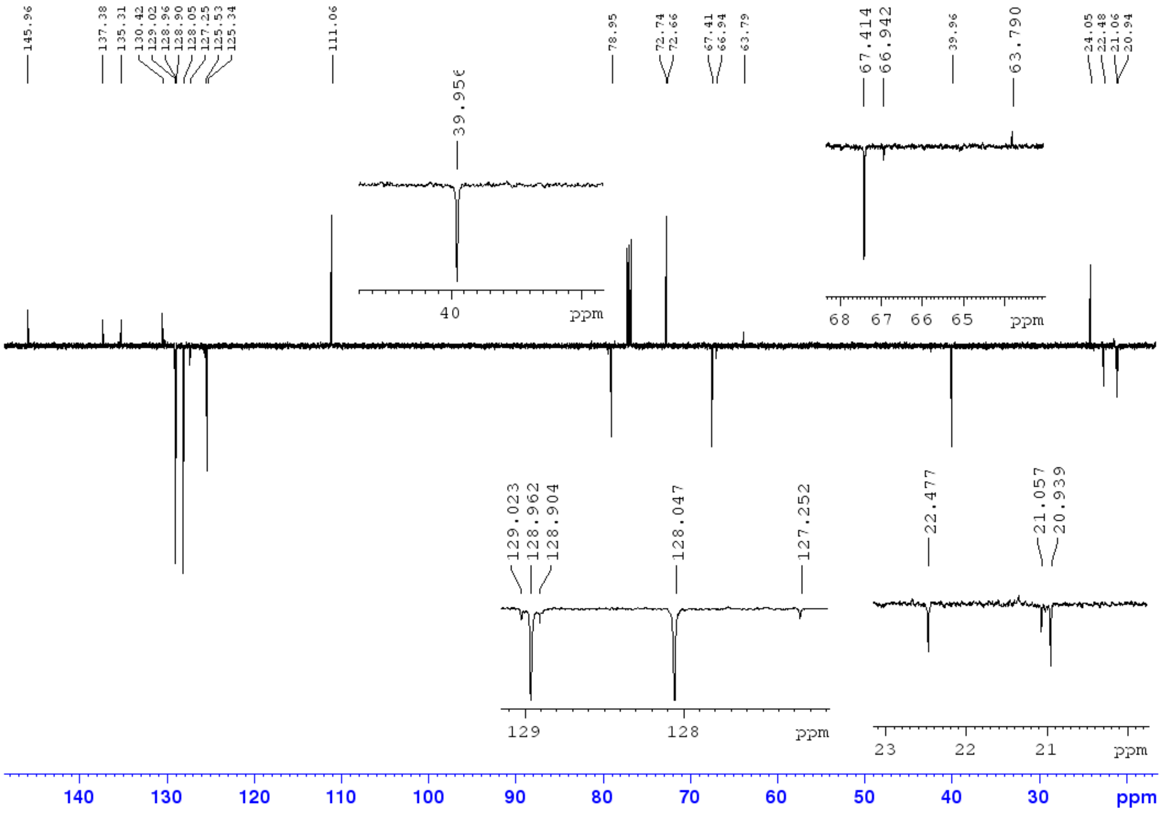

Figure S29: ^{13}C (150 MHz, $CDCl_3$) spectrum of **21**.

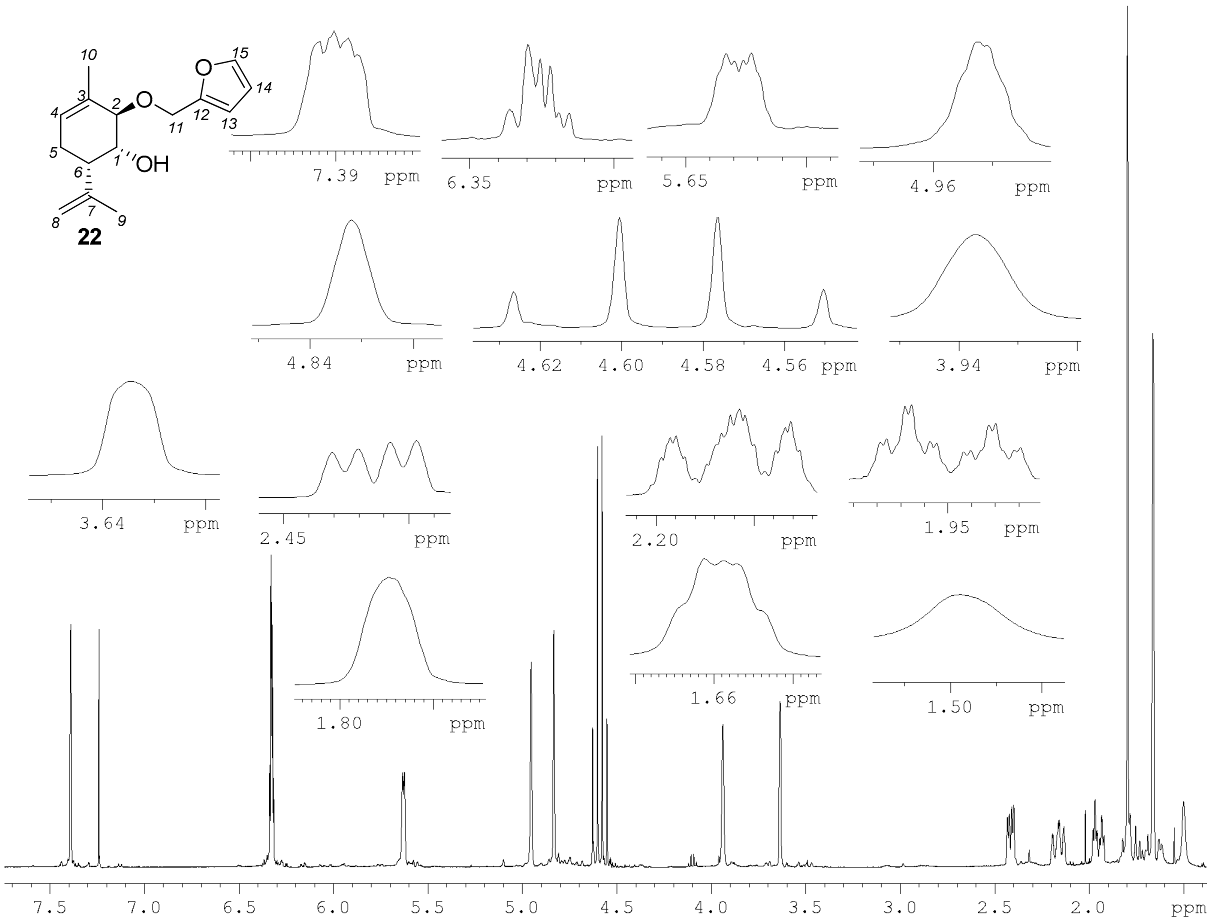

Figure S30: ^{1}H (500 MHz, $CDCl_3$) spectrum of **22**.

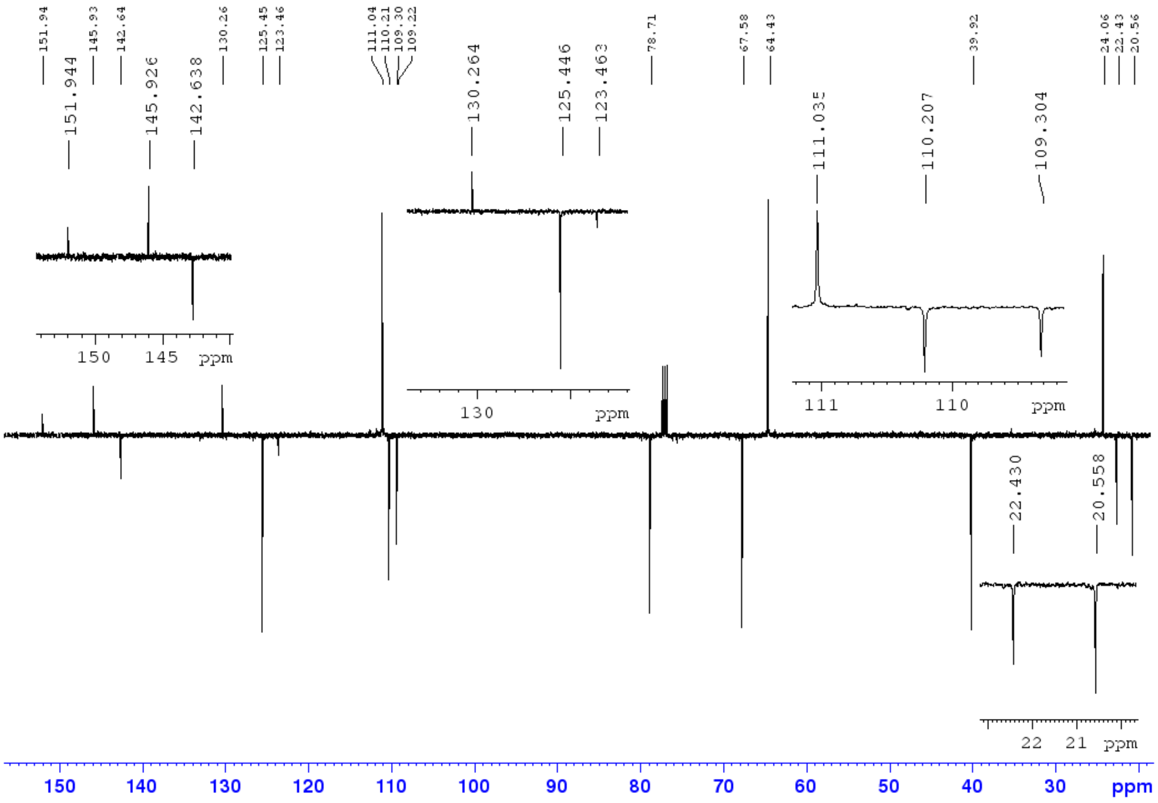

Figure S31: ^{13}C (125 MHz, $CDCl_3$) spectrum of **22**.

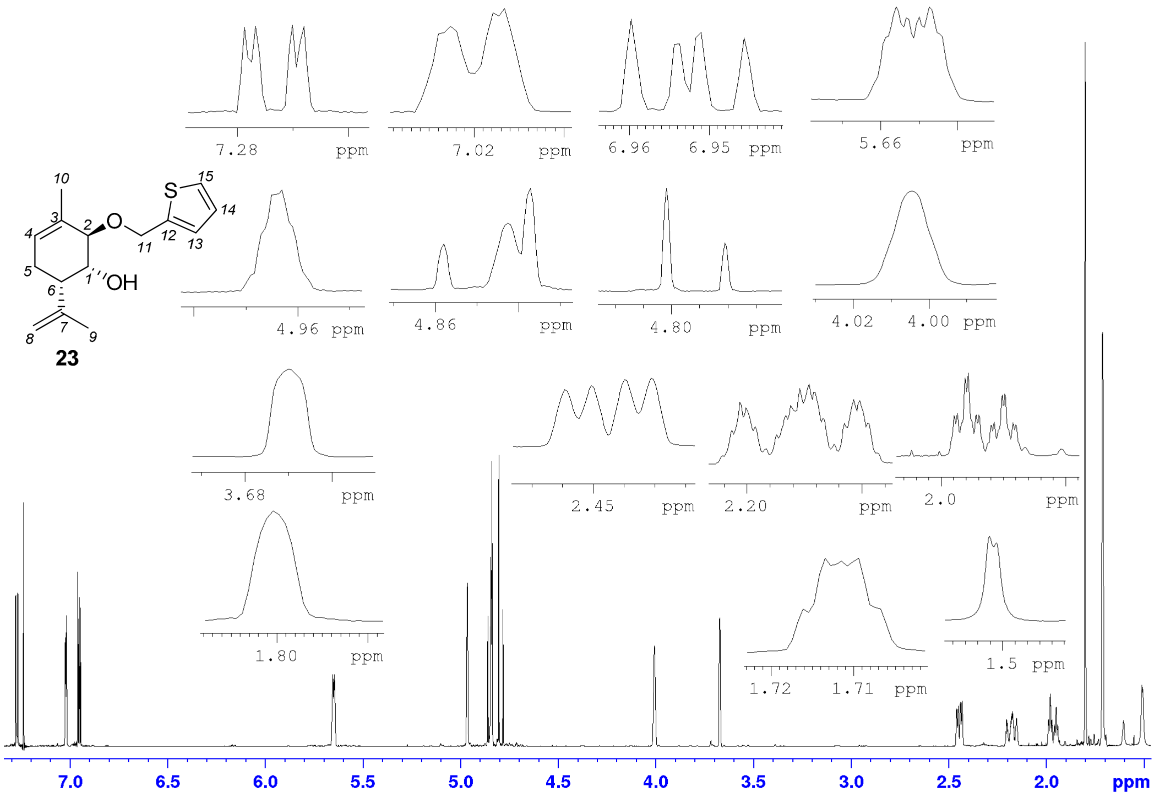

Figure S32: ^{1}H (600 MHz, $CDCl_3$) spectrum of **23**.

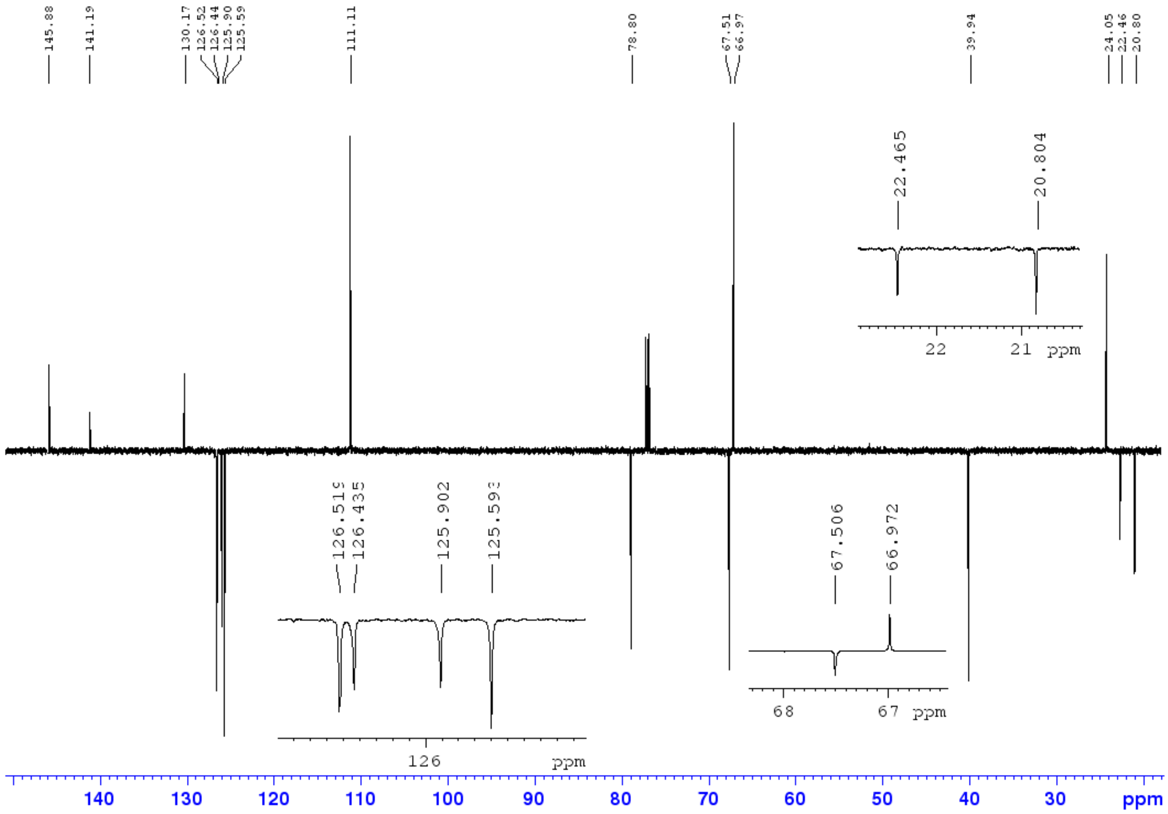

Figure S33: ^{13}C (150 MHz, $CDCl_3$) spectrum of **23**.

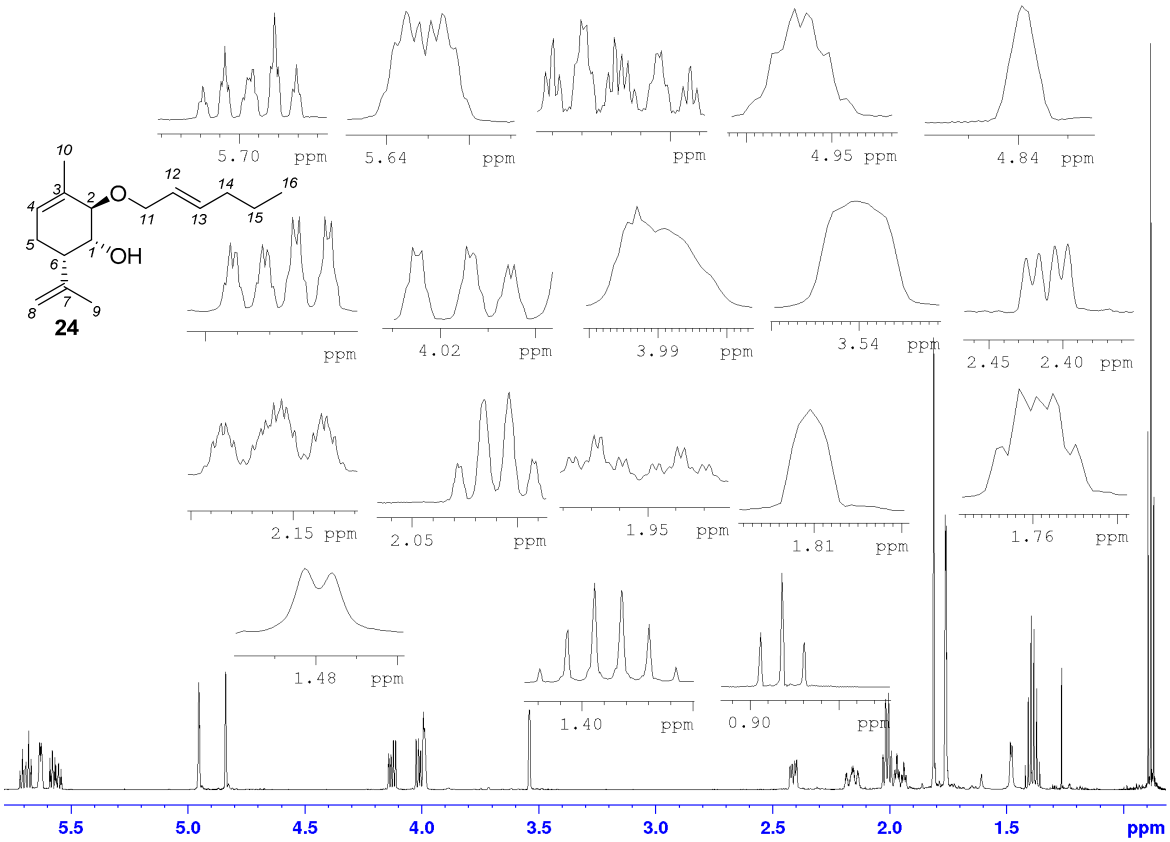

Figure S34: ^{1}H (600 MHz, $CDCl_3$) spectrum of **24**.

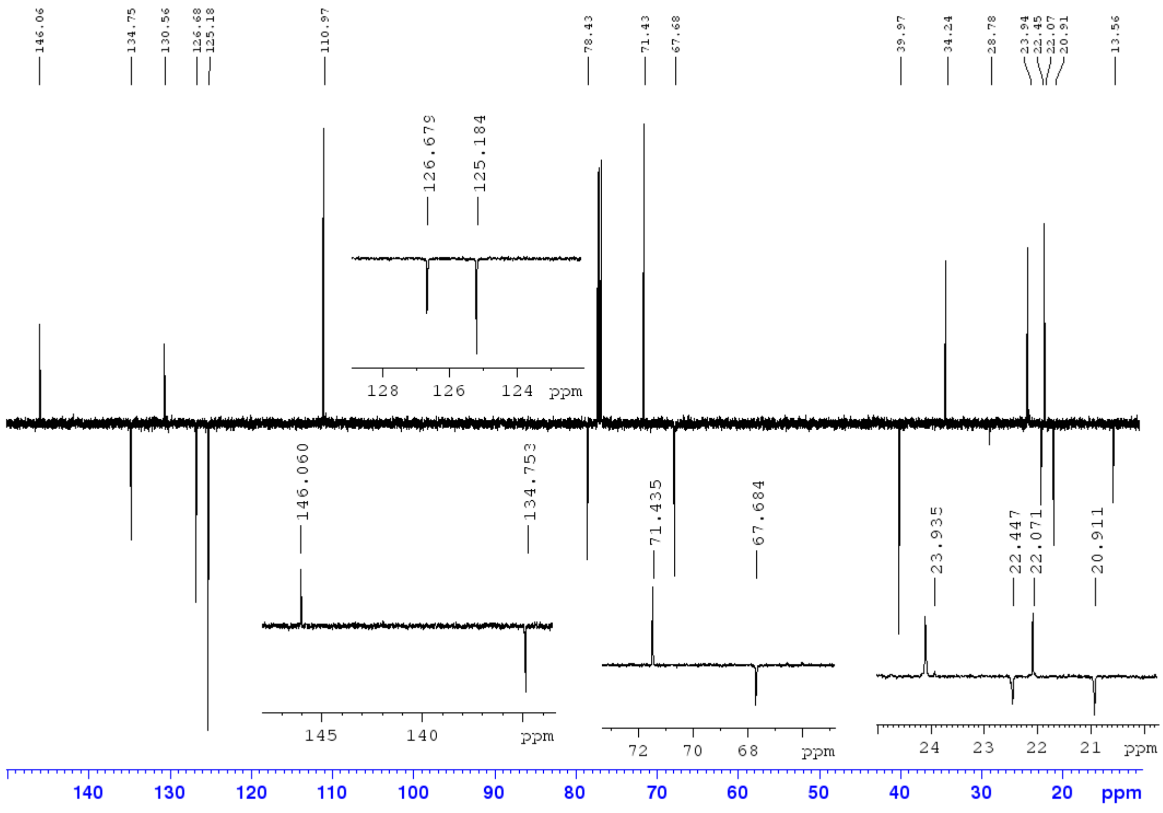

Figure S35: ^{13}C (150 MHz, $CDCl_3$) spectrum of **24**.

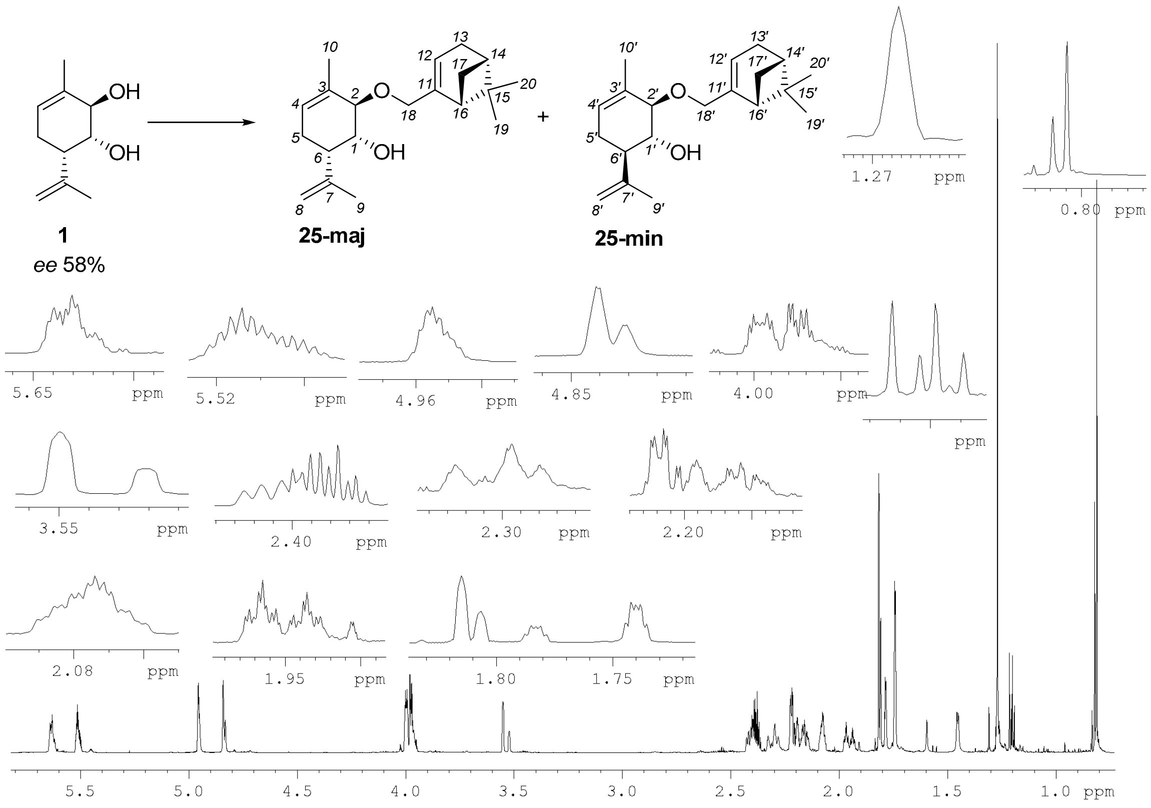

Figure S36: ^{1}H (600 MHz, $CDCl_3$) spectrum of **25-maj** and **25-min**.

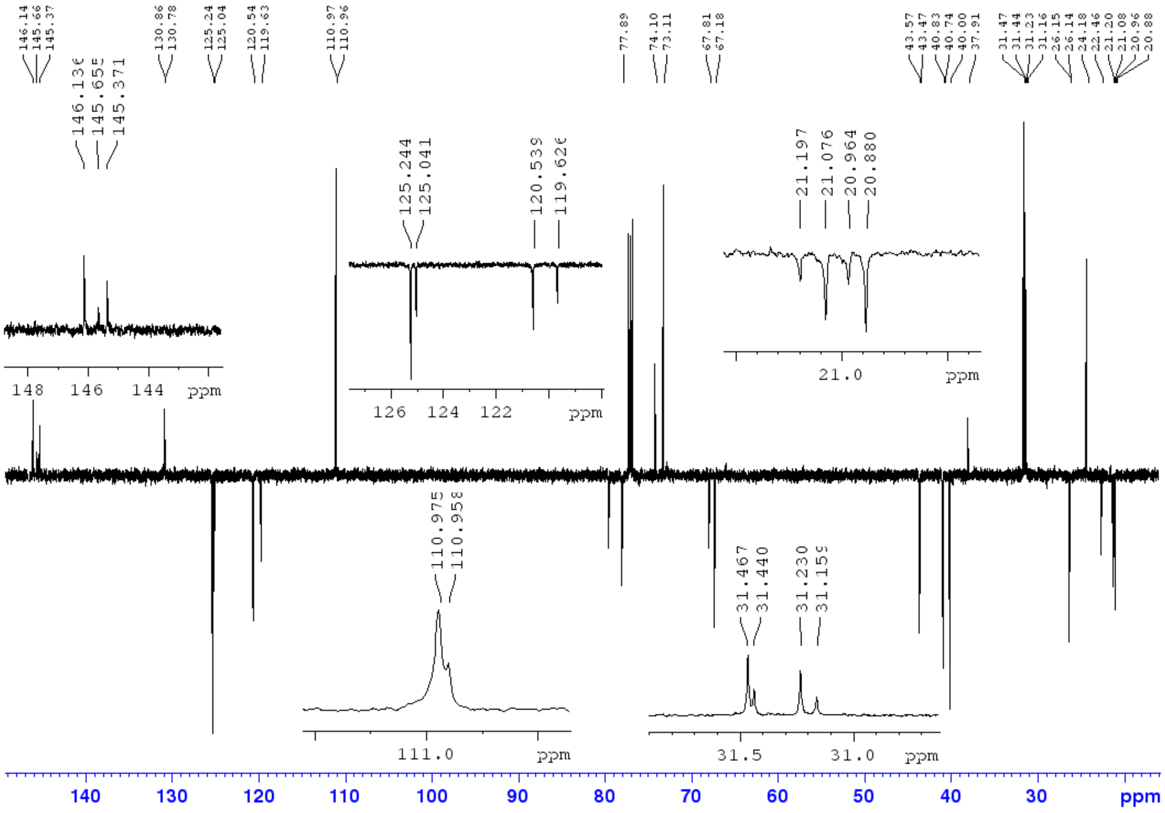

Figure S37: ^{13}C (150 MHz, $CDCl_3$) spectrum of **25-maj** and **25-min**.

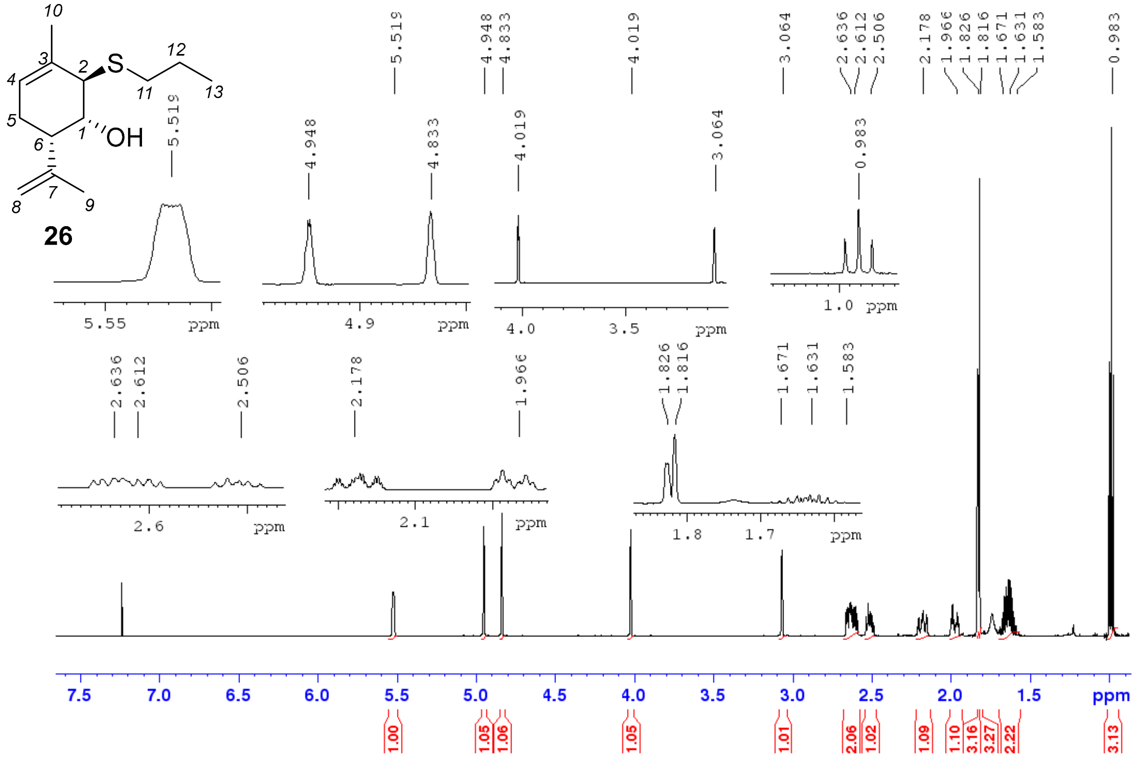

Figure S38: ^{1}H (600 MHz, $CDCl_3$) spectrum of **26**.

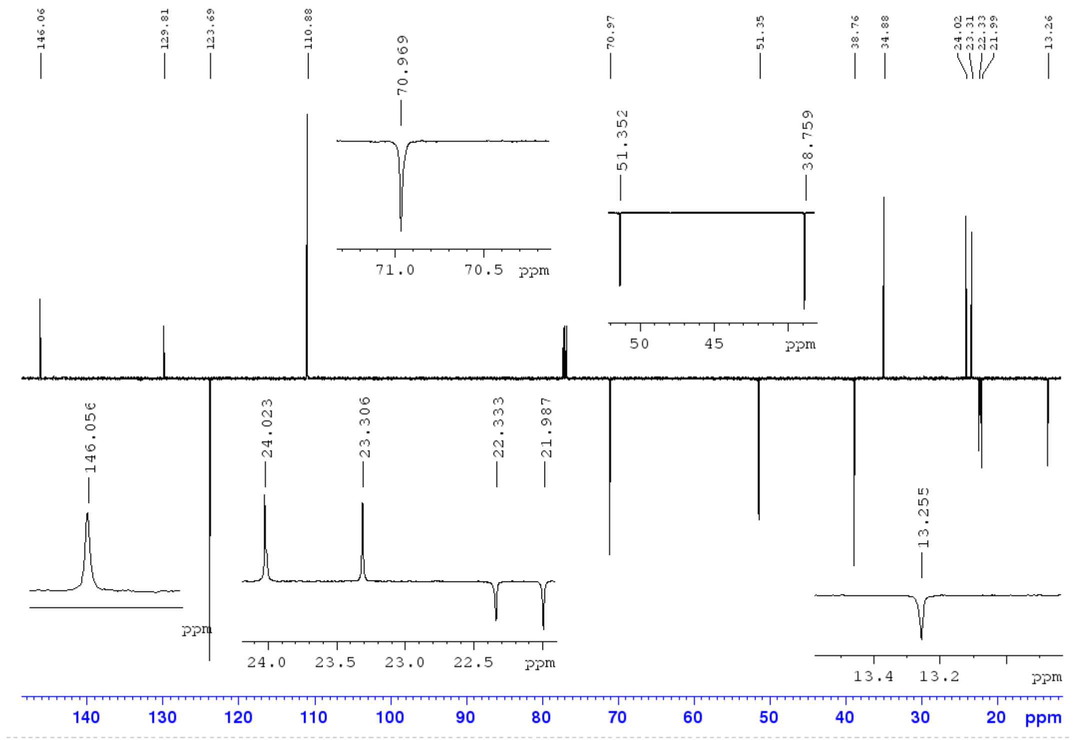

Figure S39: ^{13}C (150 MHz, $CDCl_3$) spectrum of **26**.

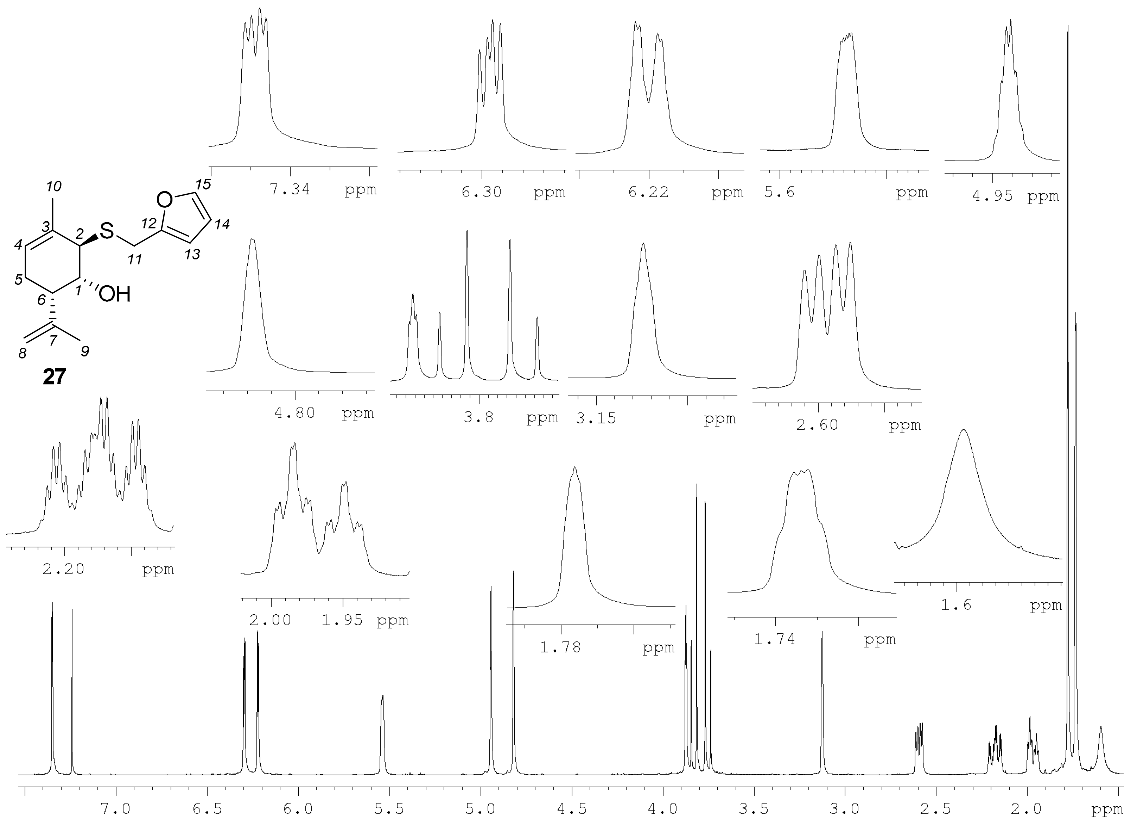

Figure S40: ^{1}H (500 MHz, $CDCl_3$) spectrum of **27**.

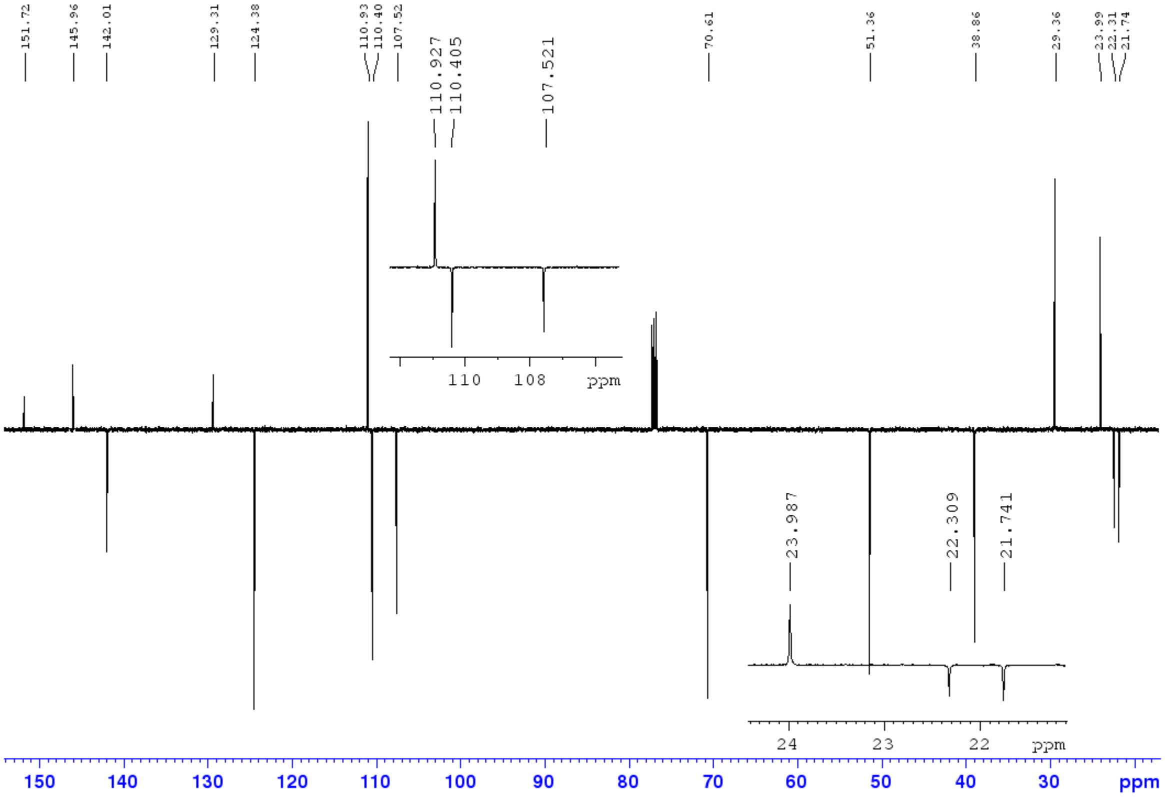

Figure S41: ^{13}C (125 MHz, $CDCl_3$) spectrum of **27**.

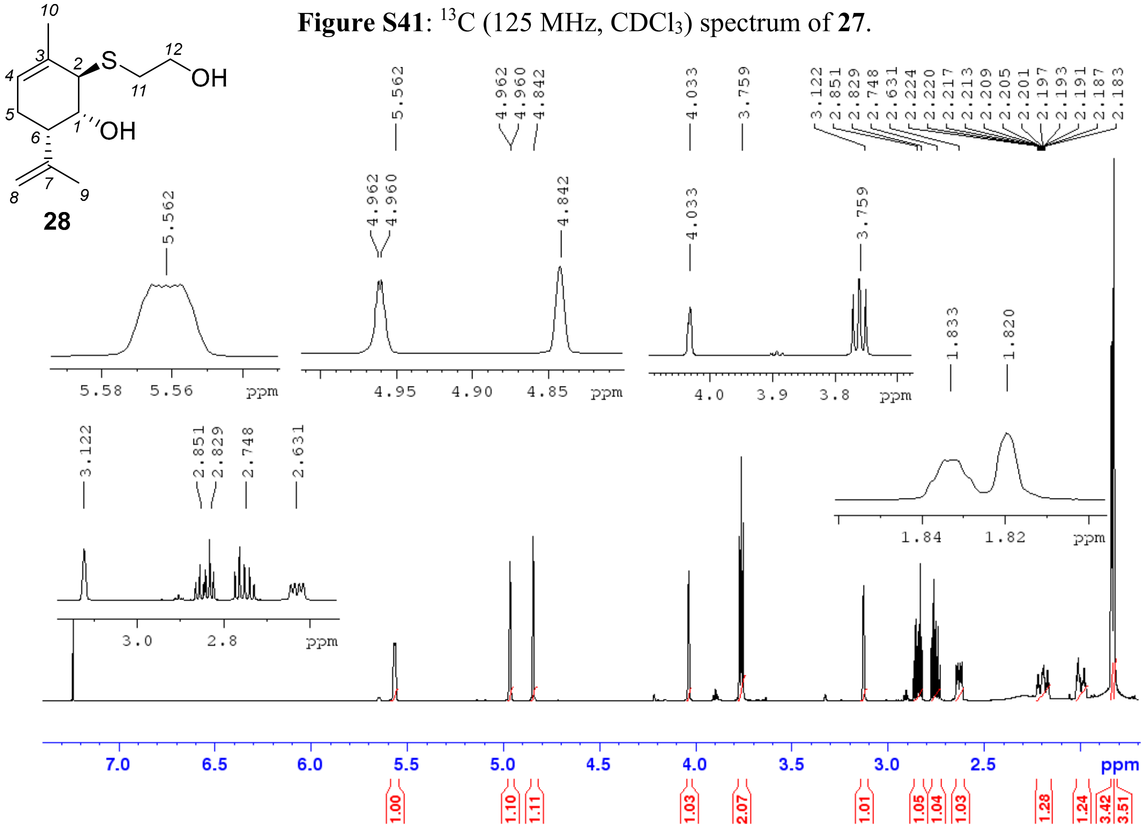

Figure S42: ^{1}H (600 MHz, $CDCl_3$) spectrum of **28**.

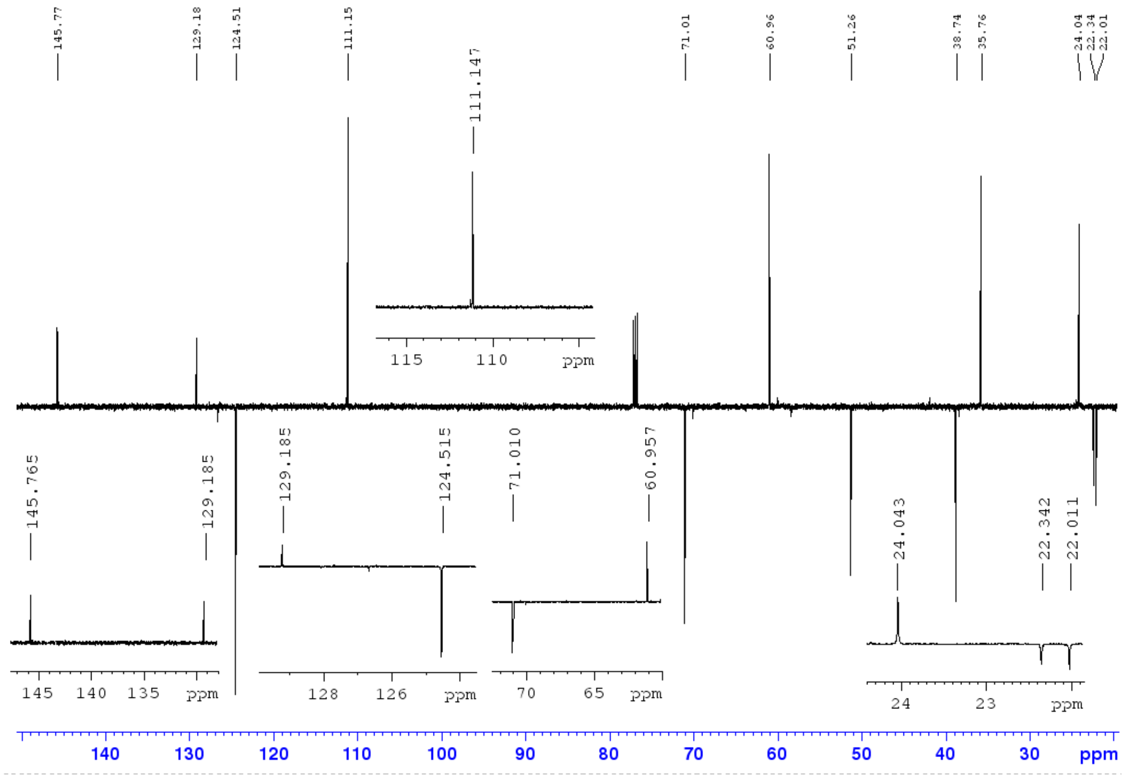

Figure S43: ^{13}C (150 MHz, $CDCl_3$) spectrum of **28**.

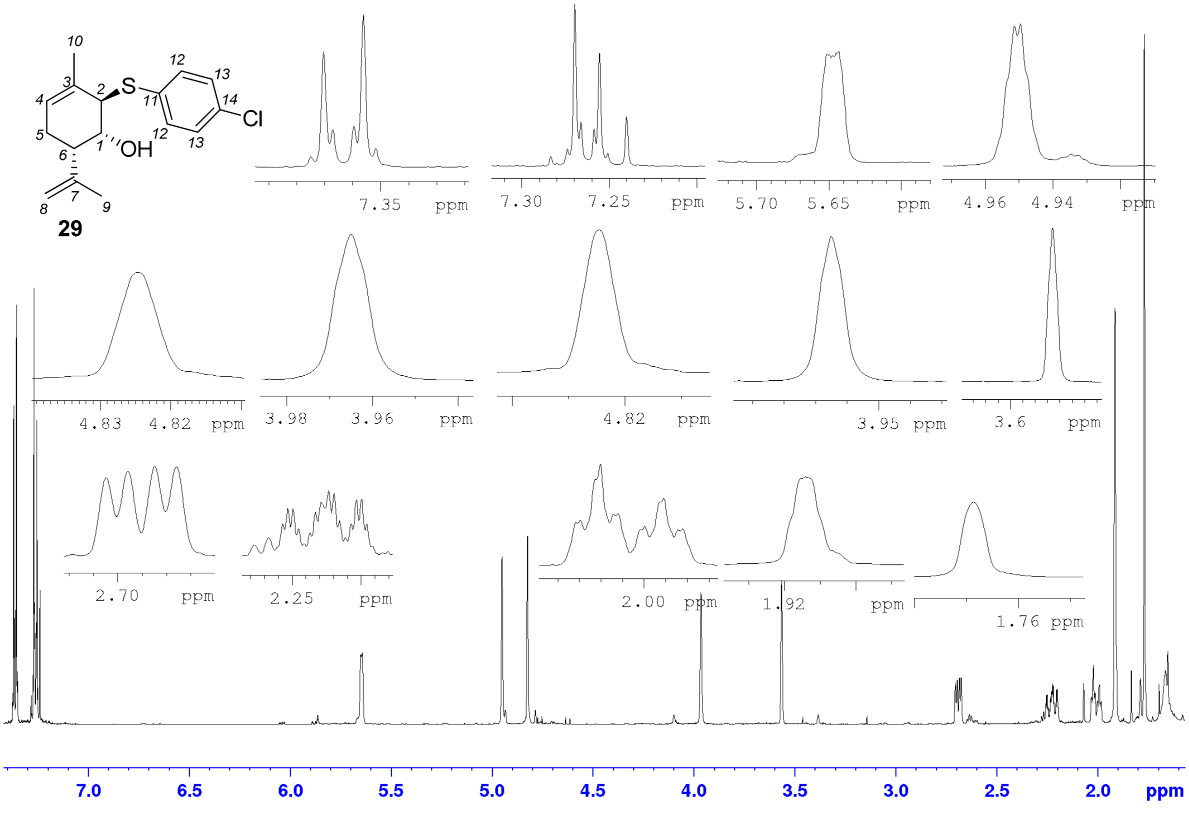

Figure S44: ^{1}H (600 MHz, $CDCl_3$) spectrum of **29**.

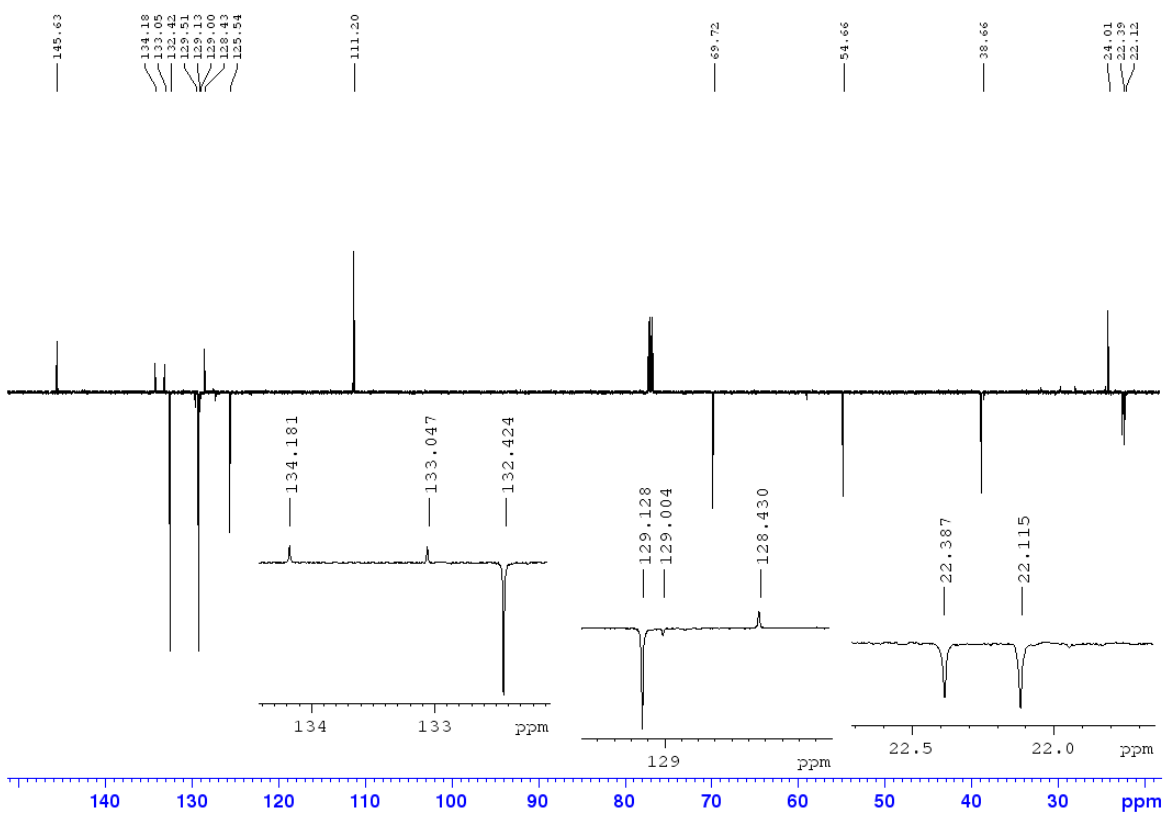

Figure S45: ^{13}C (150 MHz, $CDCl_3$) spectrum of **29**.

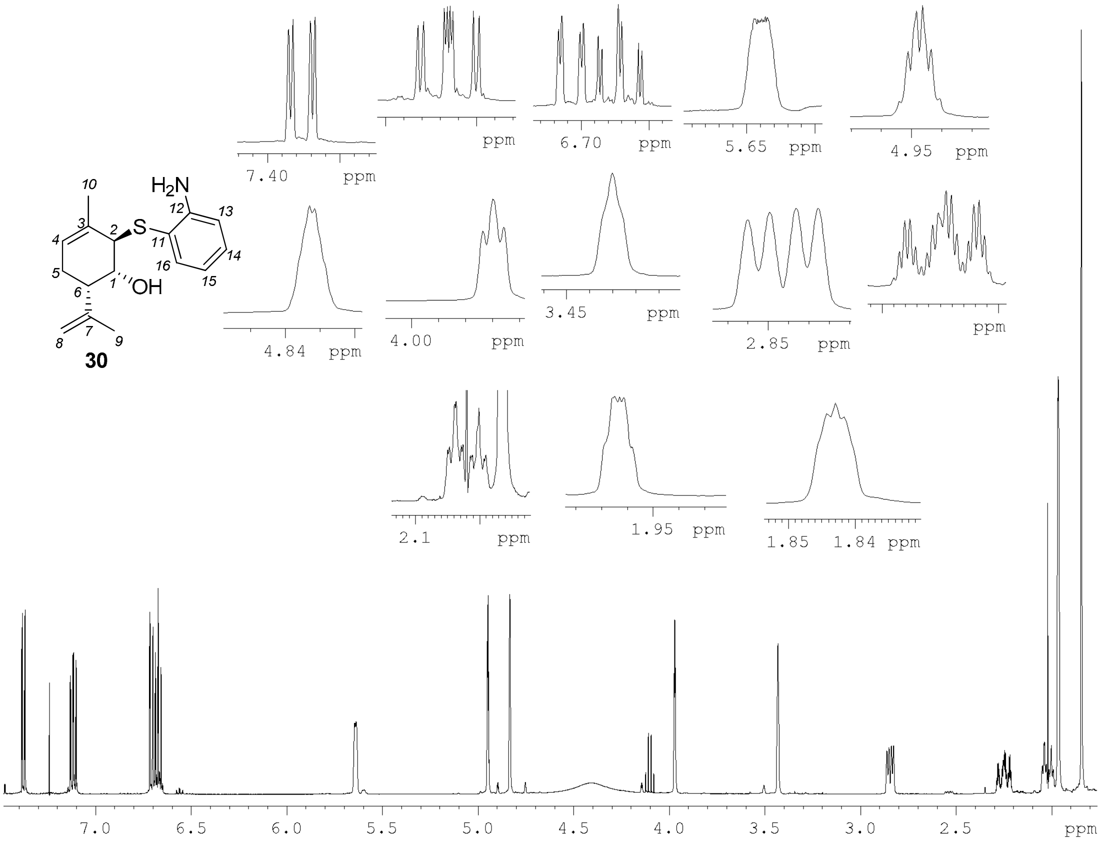

Figure S46: ^{1}H (500 MHz, $CDCl_3$) spectrum of **30**.

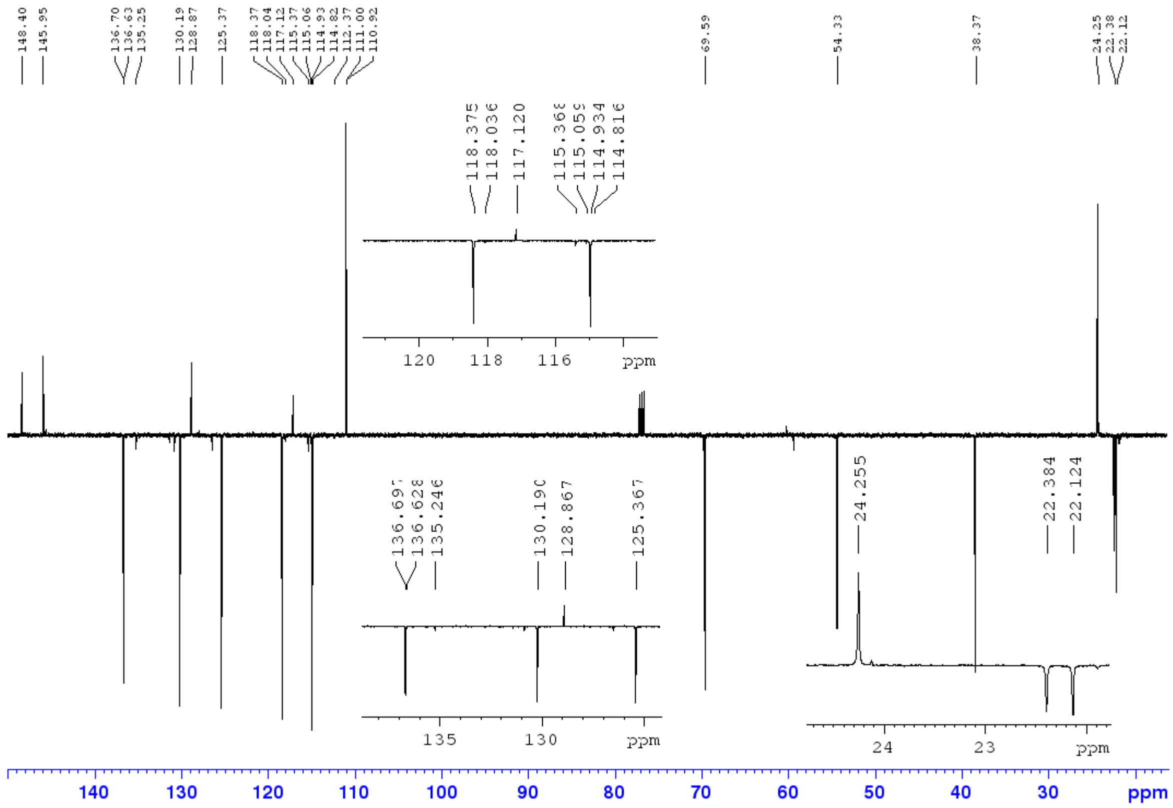

Figure S47: ^{13}C (150 MHz, $CDCl_3$) spectrum of **30**.

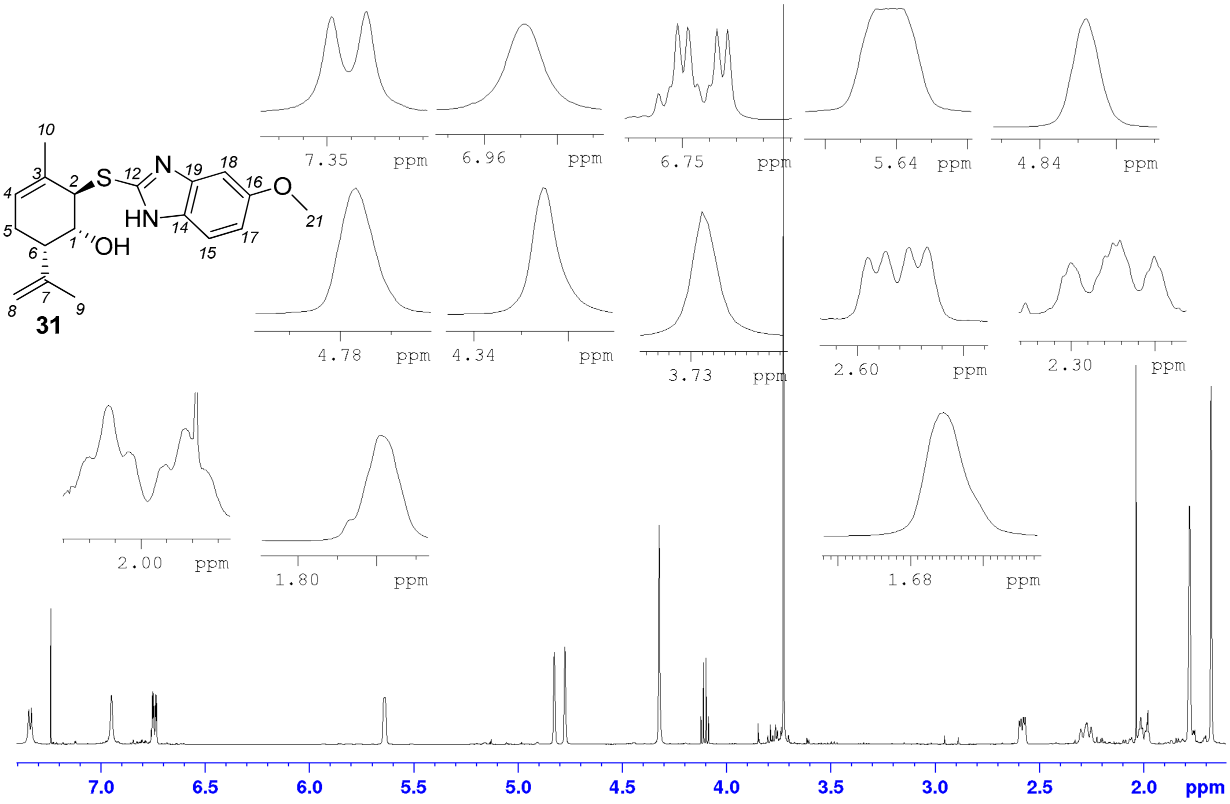

Figure S48: ^{1}H (600 MHz, $CD_3OD/CDCl_3$) spectrum of **31**.

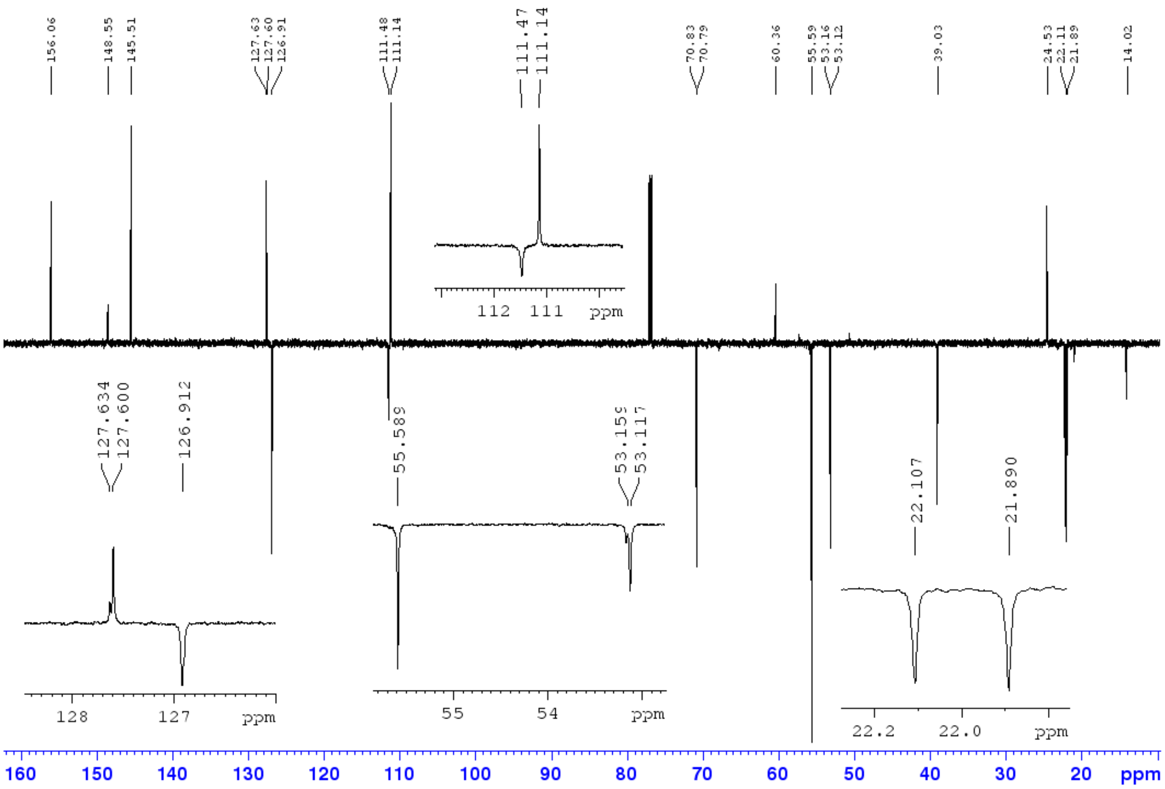

Figure S49: ^{13}C (150 MHz, $CD_3OD/CDCl_3$) spectrum of **31**.

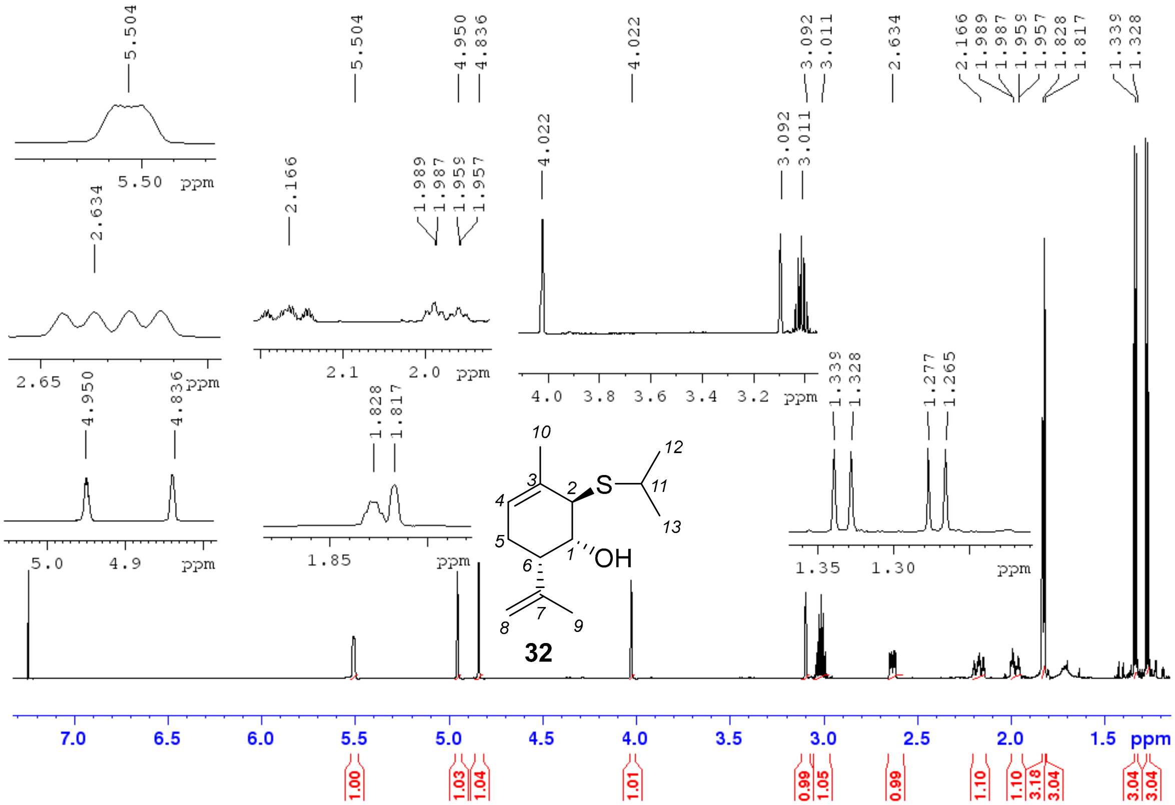

Figure S50: ^{1}H (600 MHz, $CDCl_3$) spectrum of **32**.

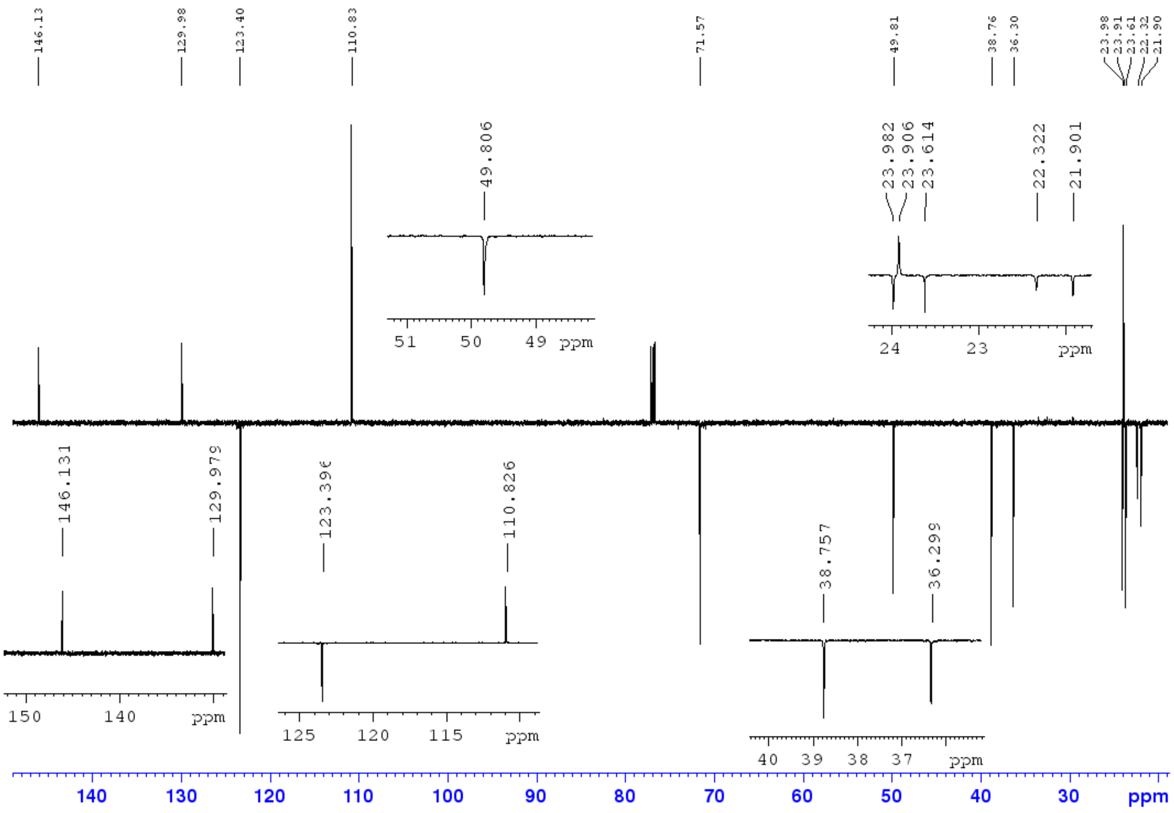

Figure S51: ^{13}C (150 MHz, $CDCl_3$) spectrum of **32**.

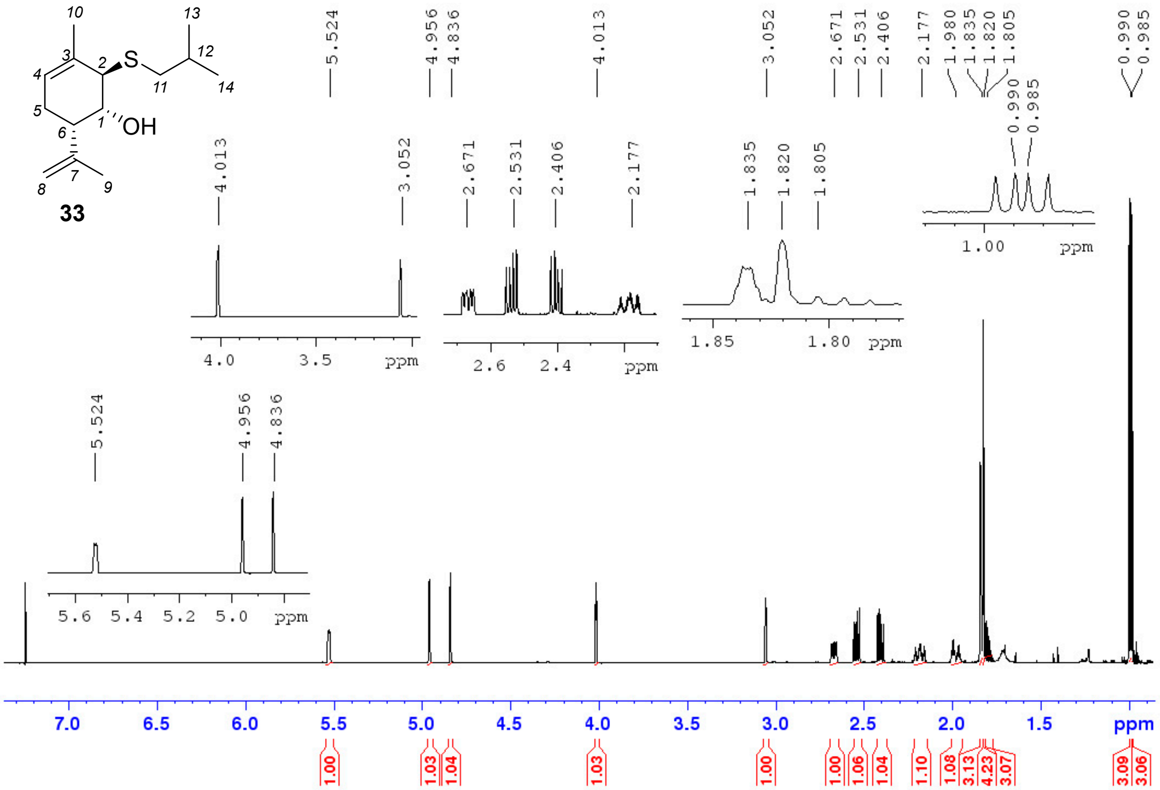

Figure S52: ^{1}H (600 MHz, $CDCl_3$) spectrum of **33**.

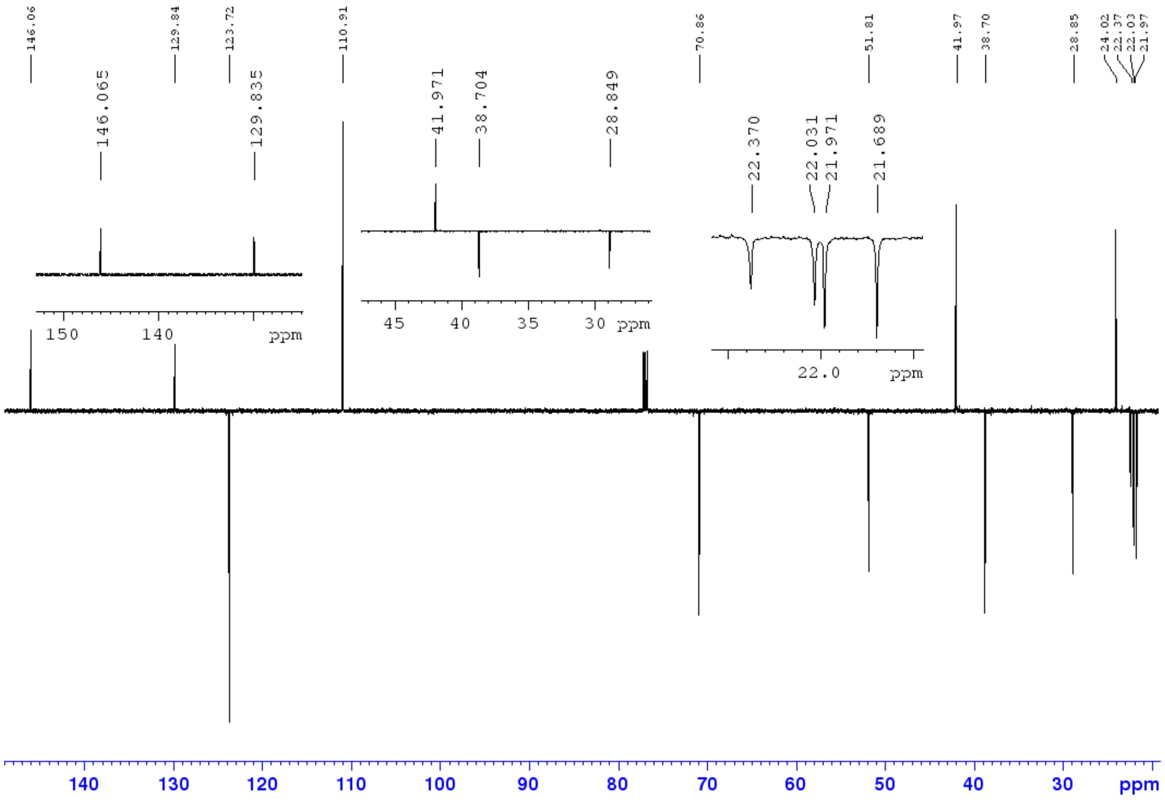

Figure S53: ^{13}C (150 MHz, $CDCl_3$) spectrum of **33**.

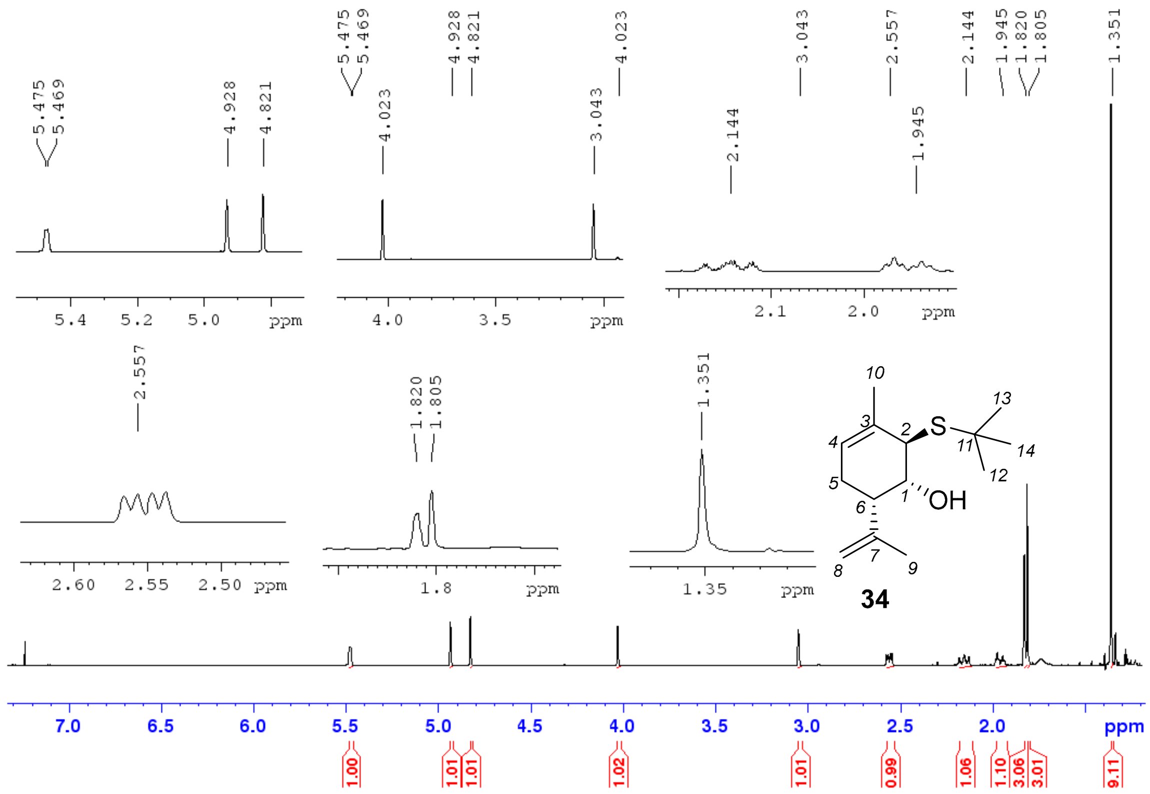

Figure S54: ^{1}H (600 MHz, $CDCl_3$) spectrum of **34**.

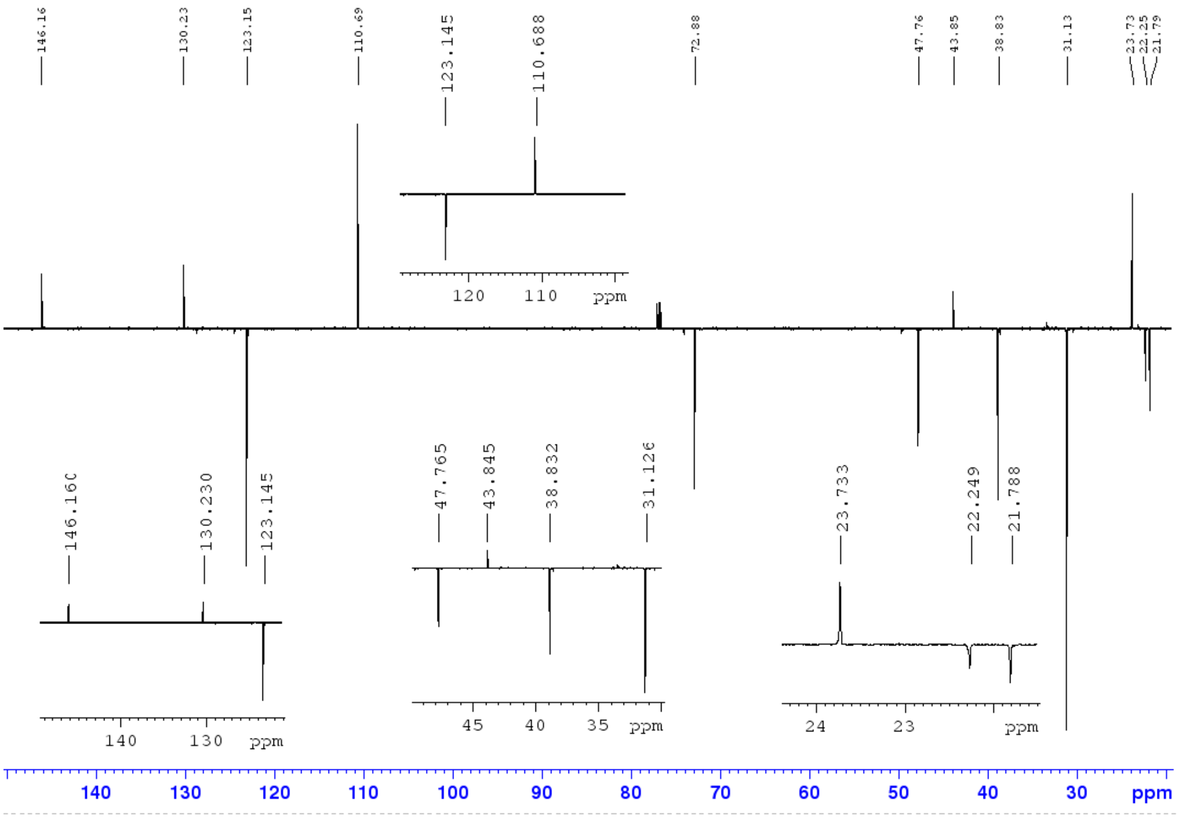

Figure S55: ^{13}C (150 MHz, $CDCl_3$) spectrum of **34**.

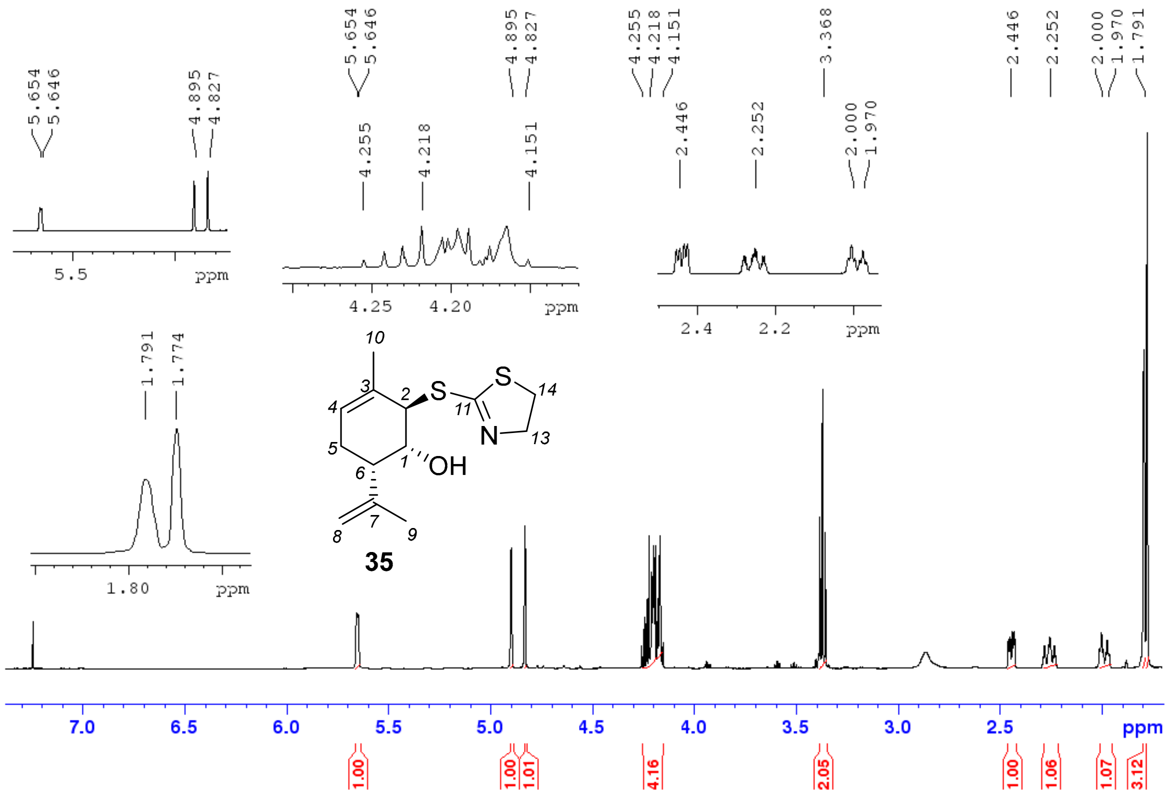

Figure S56: ^{1}H (600 MHz, $CDCl_3$) spectrum of **35**.

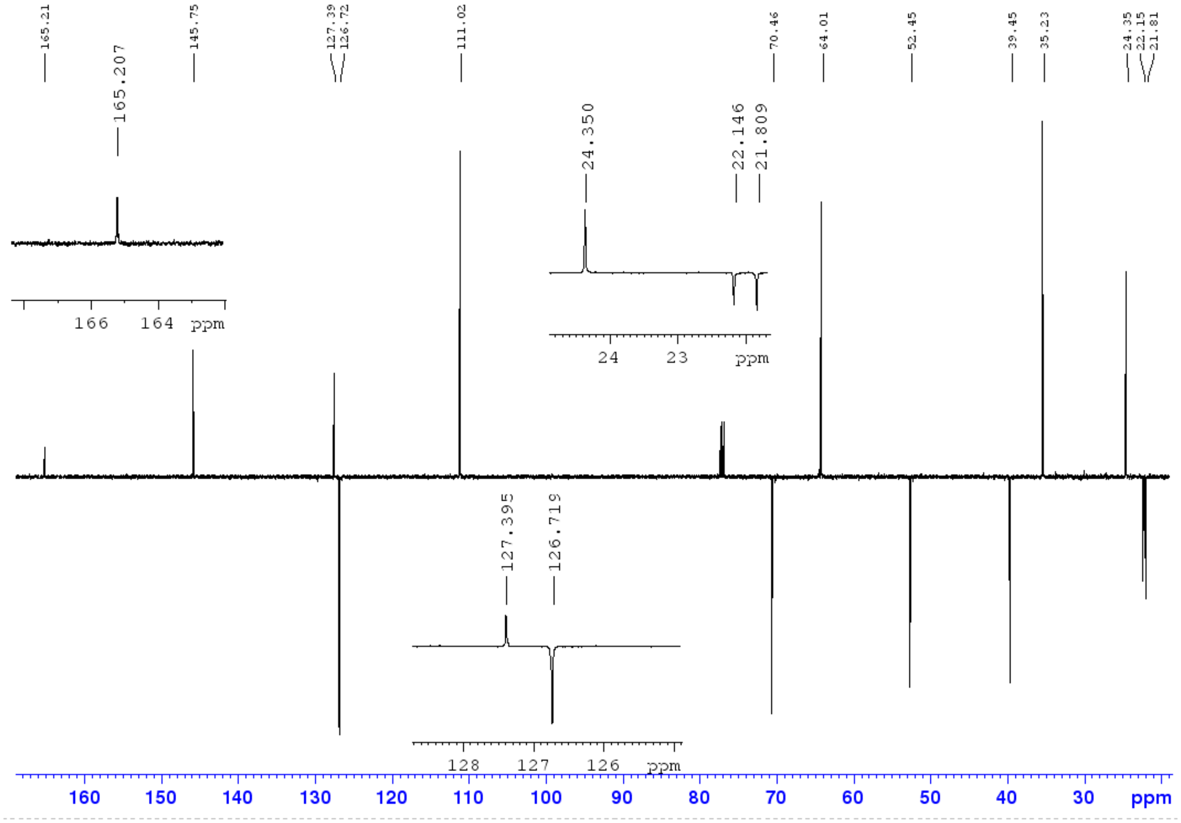

Figure S57: ^{13}C (150 MHz, $CDCl_3$) spectrum of **35**.

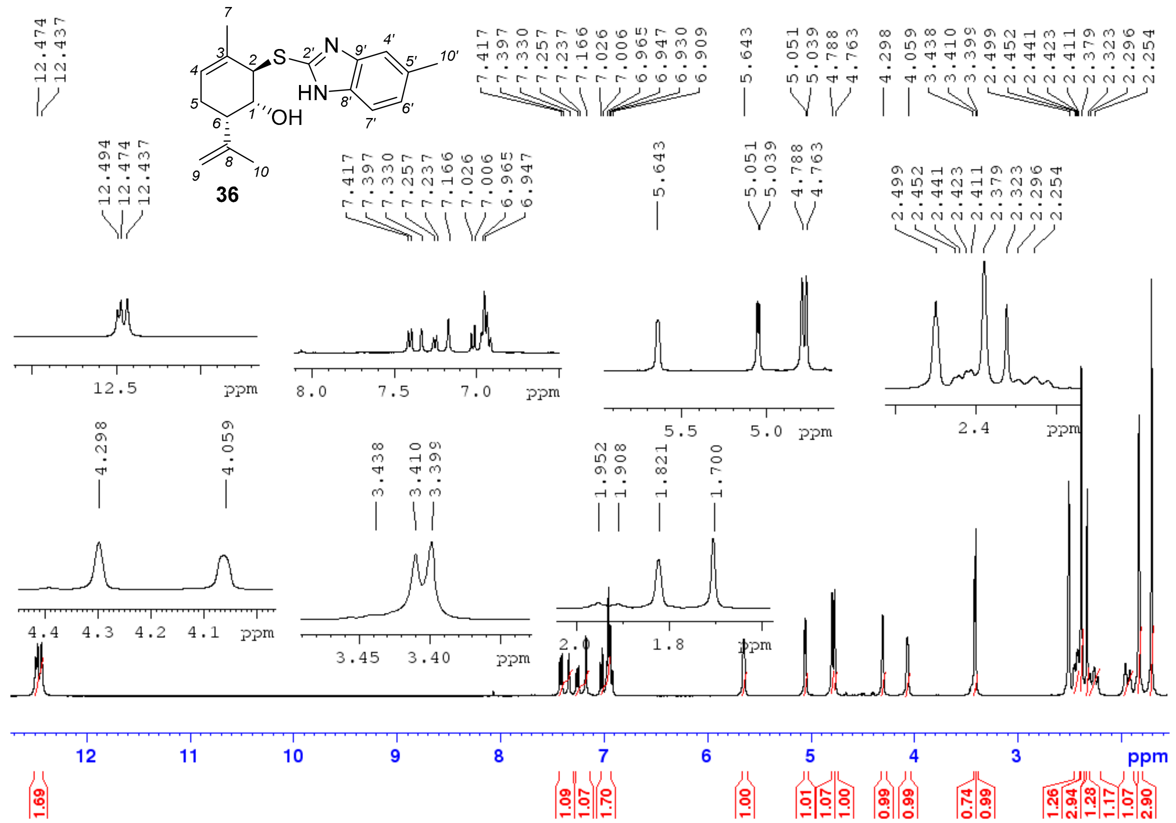

Figure S58: ^{1}H (600 MHz, d^6-DMSO) spectrum of **36**.

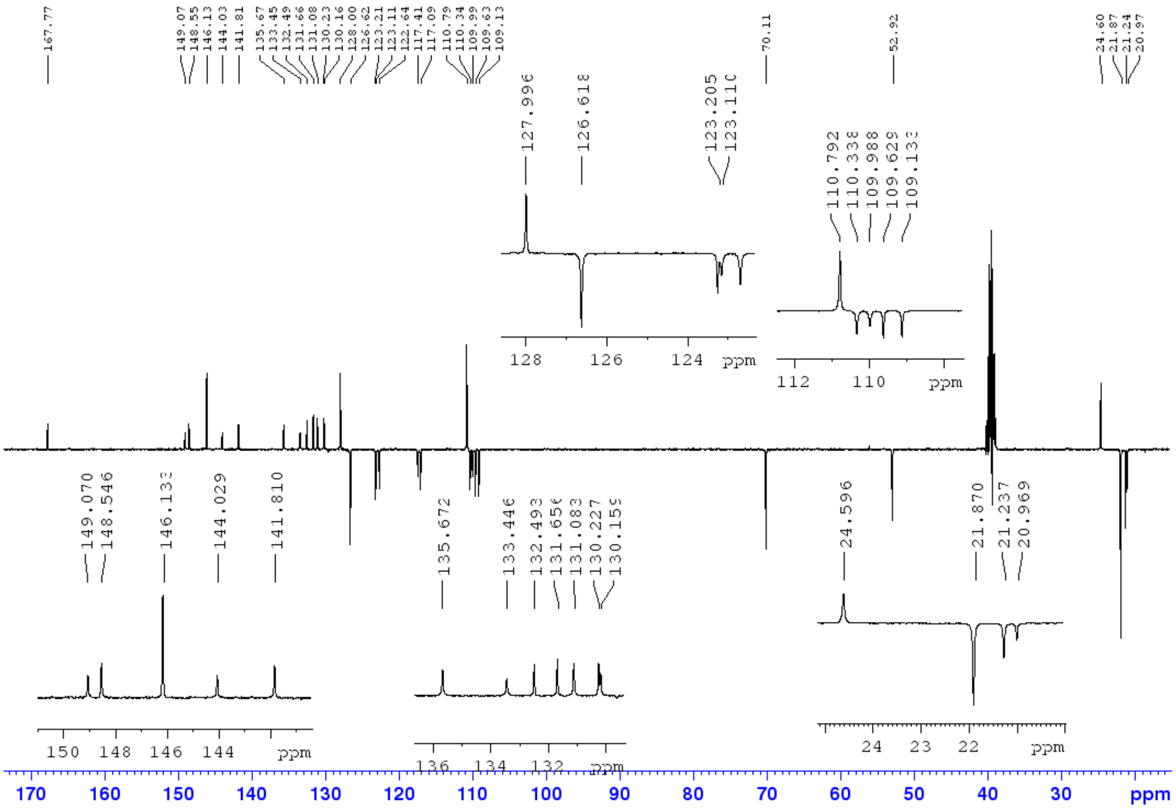

Figure S59: ^{13}C (150 MHz, d^6-DMSO) spectrum of **36**.

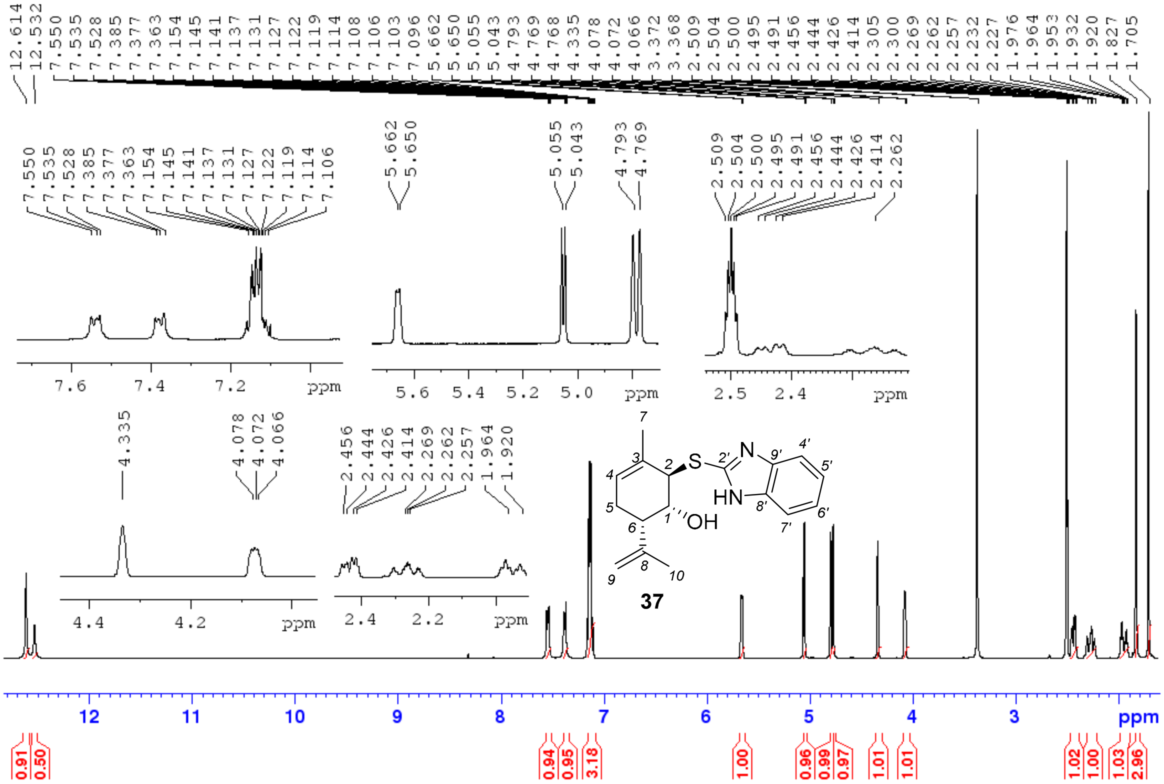

Figure S60: ^{1}H (600 MHz, d^6-DMSO) spectrum of **37**.

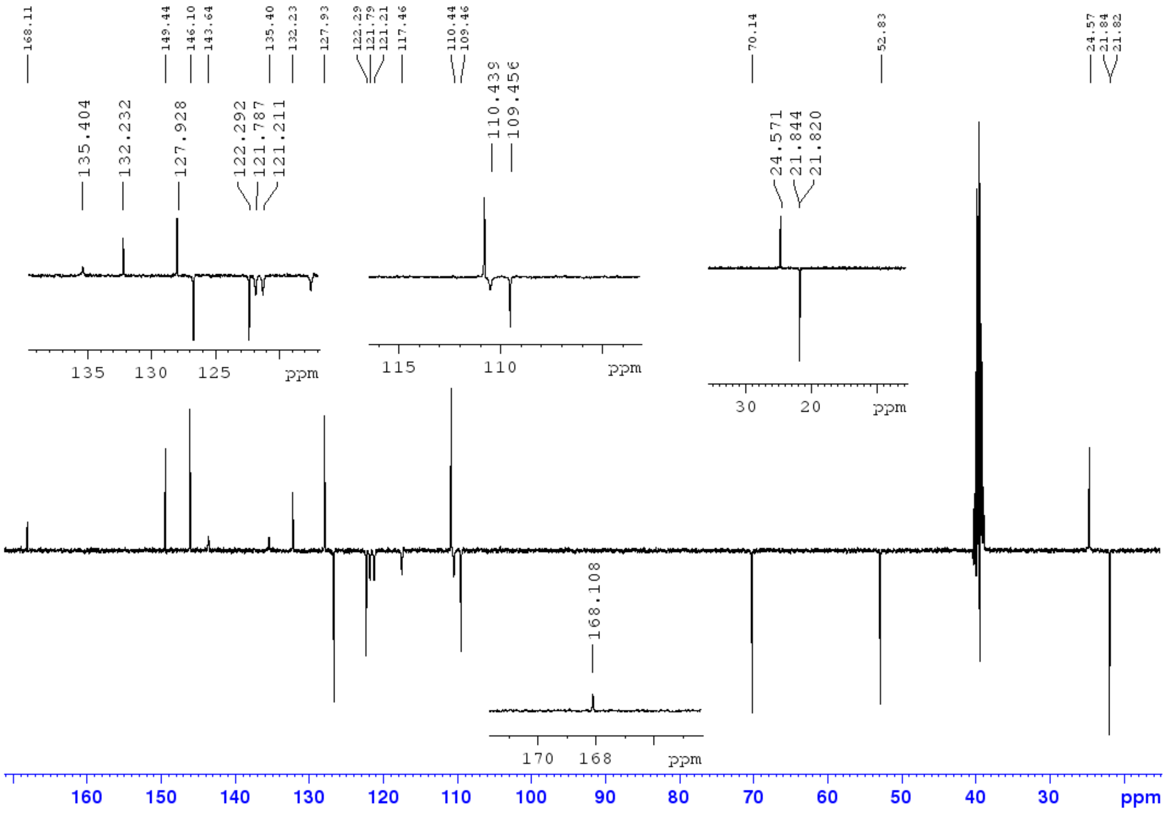

Figure S61: ^{13}C (150 MHz, d^6-DMSO) spectrum of **37**.

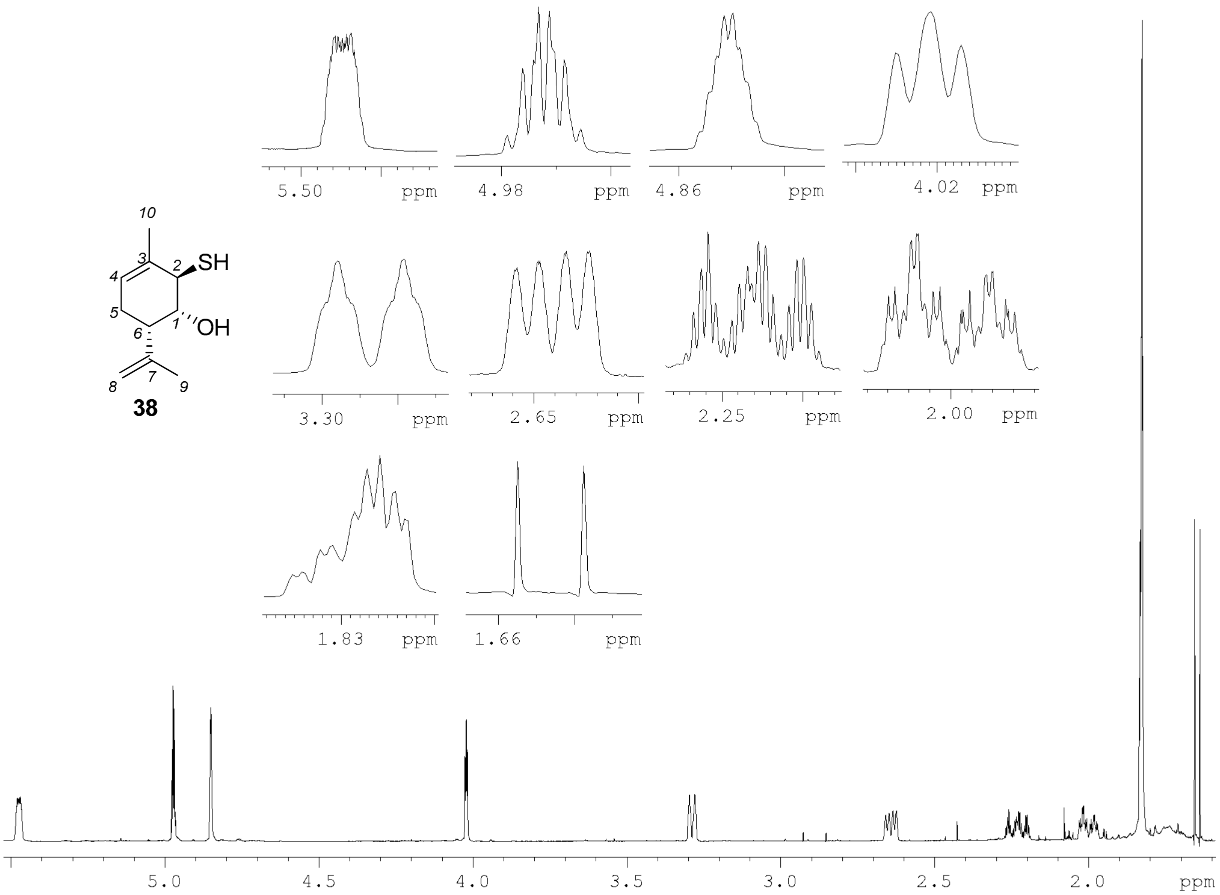

Figure S62: ^{1}H (500 MHz, $CDCl_3$) spectrum of **38**.

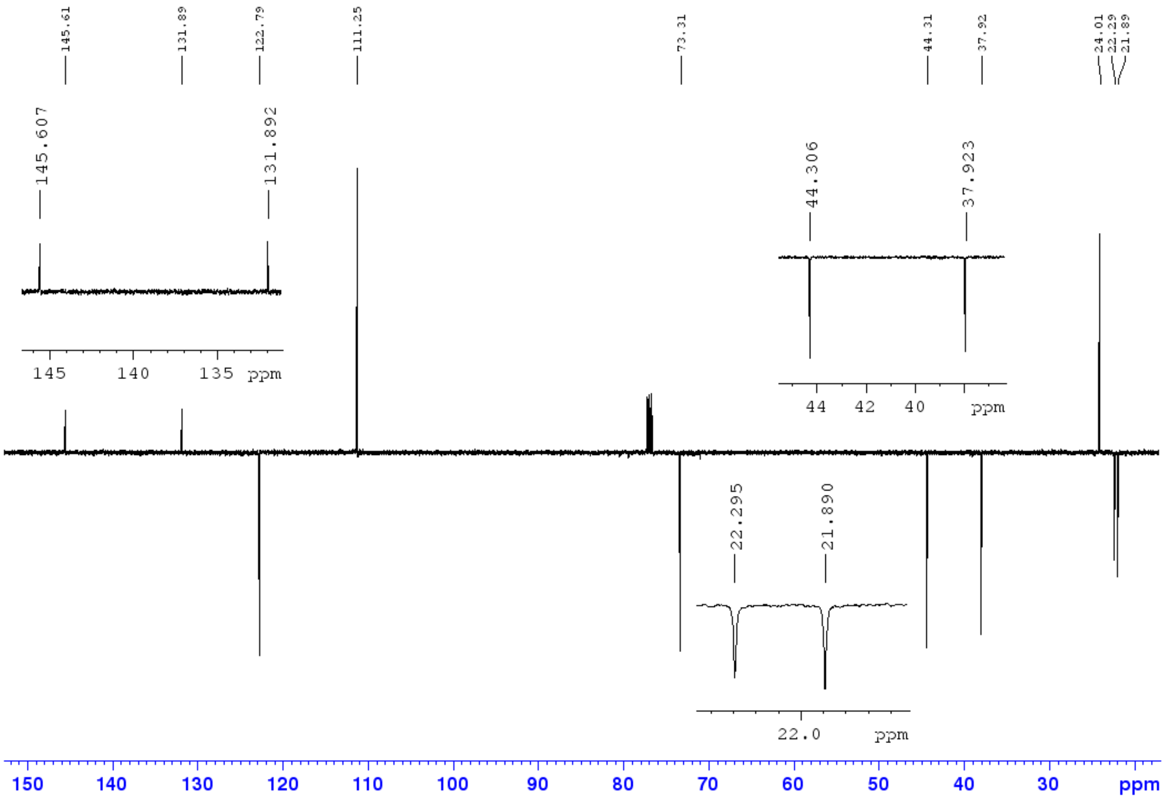

Figure S63: ^{13}C (125 MHz, $CDCl_3$) spectrum of **38**.
